# Sustainable Ammonia Electrosynthesis Coupled With Glycerol Valorization via an Adaptive Tri‐Component Catalyst

**DOI:** 10.1002/anie.202522014

**Published:** 2026-02-01

**Authors:** Christean Nickel, David Leander Troglauer, Chia‐Yu Chang, Tiansheng Bai, Tobias Rios‐Studer, Ingo Lieberwirth, Kevin Sowa, Boris Mashtakov, Bahareh Feizi Mohazzab, Lijie Ci, Deping Li, Xiaohang Lin, Bing Joe Hwang, Rongji Liu, Dandan Gao

**Affiliations:** ^1^ Department of Chemistry Johannes Gutenberg University Mainz Mainz Germany; ^2^ Sustainable Electrochemical Energy Development (SEED) Center National Taiwan University of Science and Technology Taipei Taiwan; ^3^ State Key Laboratory of Advanced Welding and Joining School of Materials Science and Engineering Harbin Institute of Technology (Shenzhen) Shenzhen People's Republic of China; ^4^ Department of Physical Chemistry of Polymers Max Planck Institute For Polymer Research Mainz Germany; ^5^ Key Laboratory for Liquid‐Solid Structural Evolution and Processing of Materials Ministry of Education, School of Materials Science and Engineering, Shandong University Jinan People's Republic of China

**Keywords:** ammonia electrosynthesis, electrochemical coupled system, glycerol valorization, pulsed electrolysis, tandem catalysts

## Abstract

Electrochemical nitrate reduction represents a promising route for sustainable ammonia (NH_3_) production, yet its practical deployment is constrained by the limited efficiency of state‐of‐the‐art electrocatalysts and immature system architectures. Here, we report a generalist copper–nickel–tungsten tri‐component tandem electrocatalyst via a sequential microwave‐hydrothermal deposition route. Under pulsed electrolysis conditions, the catalyst delivers a remarkable Faradaic efficiency of 97.1% and a record‐high ammonia yield rate of 43.87 mg h^−1^ cm^−2^. Online differential electrochemical mass spectrometry (DEMS) identifies key intermediates and associated pathways, while density functional theory (DFT) calculations elucidate the cooperative roles of each component: the copper component facilitates nitrate adsorption and deoxygenation, the nickel component promotes water dissociation for steady *H supply, and the tungsten component serves as a dynamic *H reservoir. This synergy efficiently suppresses hydrogen evolution and enhances ammonia selectivity. Furthermore, coupling with glycerol valorization (to formic acid) as the anodic reaction demonstrates the potential for energy‐efficient ammonia electrosynthesis. Collectively, this work offers both design strategies and mechanistic understanding for next‐generation multi‐component tandem electrocatalysts targeting advanced nitrogen‐based chemical synthesis.

## Graphical abstract

A Cu‐Ni‐W tri‐component catalyst enables efficient nitrate‐to‐ammonia conversion under pulsed electrolysis. Combined experimental and theoretical studies attribute its performance to component synergism and regulated intermediates. Its facile adaptation for glycerol valorization to formic acid underscores a versatile system design concept, advancing electrochemical coupling strategies for a broad spectrum of industrially relevant reactions.

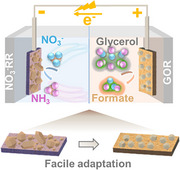

## 1 Introduction

Ammonia (NH_3_) serves as an indispensable component across the agriculture, healthcare, and chemical industries [1, 2]. Beyond these, ammonia is highly valued for its attributes as a promising carbon‐free and hydrogen‐rich fuel for future energy systems [3, 4]. Presently, industrial‐scale NH_3_ production is predominantly reliant on the conversion‐inefficient and energy‐intensive fossil‐fuel‐driven Haber–Bosch process [5, 6], which necessitates the thermal cleavage of inert N≡N bonds (dissociation energy: 941 kJ mol^−1^) under stringent conditions and has a significant carbon footprint due to its reliance on H_2_ derived from natural gas steam reforming [7, 8]. In light of this, advancing sustainable NH_3_ synthesis technologies is imperative to alleviate energy consumption and mitigate associated environmental impact.

In this context, the electrochemical nitrate reduction reaction (NO_3_^−^RR) powered by renewably “green” electricity has emerged as a promising approach for sustainable NH_3_ production under ambient conditions [9], owing to the relatively low N═O bond dissociation energy (204 kJ mol^−1^), superior water solubility, and widespread availability of nitrate [10]. Unlike the carbon‐emitting Haber–Bosch process, NO_3_^−^RR utilizes water as the proton source, providing a direct pathway for decarbonization [11]. Moreover, adopting ubiquitous nitrate‐bearing water bodies (e.g., industrial wastewater, polluted groundwater) as the nitrogen feedstock enables NO_3_^−^RR to serve a dual purpose, unambiguously facilitating NH_3_ synthesis and mitigating nitrogen pollution. Despite its considerable potential, several challenges impede its practical implementation, including the intricate proton‐coupled eight‐electron transfer mechanism [12], the formation of multiple N‐containing intermediates during the sequential deoxygenation and hydrogenation steps [13], and the sluggish kinetics of the accompanying oxygen evolution reaction (OER) on the anode [14]. Overcoming these hurdles through advanced catalyst design and strategic system engineering will be crucial for enhancing NH_3_ synthesis performance and minimizing energy input.

Thus far, Cu‐based electrocatalysts [15, 16, 17, 18, 19, 20] have been profoundly employed for NO_3_^−^RR due to the alignment of Cu d‐orbital energy levels with the lowest unoccupied molecular orbital π* of NO_3_^−^, endowing strong NO_3_^−^ adsorption and its initial conversion to NO_2_^−^ [21, 22]. However, due to the high energy barrier, Cu showcases limited water dissociation activity for generating and accommodating active hydrogen species (*H), which hinders the hydrogenation of NO_2_^−^ converted from NO_3_^−^, resulting in undesired NO_2_^−^ accumulation and suboptimal NH_3_ yield [23, 24]. Meanwhile, Ni‐based electrocatalysts are widely recognized as prominent catalysts for water dissociation and *H generation [25, 26, 27], making them effective catalytic sites for the deep hydrogenation of N‐containing intermediates [28, 29, 30]. Thereinto, Cu‐Ni dual‐component tandem electrocatalysts have garnered considerable attention for NO_3_^−^RR, such as Cu nanoclusters decorated Ni_3_N [31], Cu‐Ni alloys [32, 33, 34, 35], Ni‐doped CuO [36], Ni‐modified Cu_2_O single‐atom alloy oxide [37] and Ni(OH)*_x_*/Cu nanowire arrays [38]. Despite these advancements, NO_3_^−^RR enhancement is still plagued by mismatched tandem reaction rates, particularly among deoxygenation, *H supply via water dissociation and sequential hydrogenation processes [39, 40]. This imbalance can lead to either the accumulation of N‐containing intermediates due to insufficient *H availability or a competing HER caused by excessive *H production, both adversely affecting NH_3_ yield [41]. Notably, WO_3_ has emerged as an effective *H storage medium owing to its hydrogen intercalation capability, enabling a well‐balanced *H generation, transfer, and consumption [42, 43]. Pioneering studies, such as those on Cu/Mo‐WO_3_ nanorods [44], Ru/WO_3‐_ *_x_* nanoarrays [45], WO*_x_* N*_y_*/WO_3_ and Cu_1_/WO_3_ nanosheets [46, 47], have highlighted its potential in NO_3_^−^RR, yet the investigation of Cu/Ni/WO_3_ tri‐component catalysts and their synergism in the context of NO_3_^−^RR remains largely unexplored.

Aside from catalyst development, engineering the technological framework is vital for achieving efficient NO_3_^−^RR. One emerging strategy in this regard is the incorporation of pulsed electrolysis, benefiting from enhanced mass transfer, optimized intermediate adsorption/desorption, and improved selectivity [48, 49, 50]. Additionally, replacing the typically sluggish anodic OER with a thermodynamically favorable biomass electro‐oxidation reaction represents a significant opportunity to reduce energy consumption while simultaneously generating a second value‐added chemical at the anode [51, 52]. To date, only a limited number of materials have demonstrated the versatility required for this dual‐function approach. Most pioneering studies have focused on identical electrocatalysts to drive NO_3_^−^RR‐coupled biomass electro‐oxidation systems. Examples include Cu@CoCu LDH/CC [53] for coupling with ethylene glycol oxidation reaction, Cu_2_O [54] and Ag_1_@Cu_2_O [55] for coupling with formaldehyde oxidation reaction, Co@CF [56] for coupling with methanol oxidation reaction, Cu_2_O/Cu@PdCu [57] for coupling with ethanol oxidation, Ru‐nanocluster [58] for coupling with glucose oxidation reaction, Cu_2_NCN [59] and Co_3_Mo_3_N [60] for coupling with glycerol oxidation reaction (GOR). However, these systems often encounter potential challenges arising from active site incompatibility between the cathodic and anodic reactions, which can limit the overall performance of NO_3_^−^‐RR‐to‐NH_3_. To address this, adaptive electrocatalyst systems specifically designed for coupled electrocatalysis present a promising solution. Such systems not only improve electrode utilization but also optimize the overall reaction efficiency, thus offering a potential route for enhancing NH_3_ production while reducing energy input.

**SCHEME 1:**
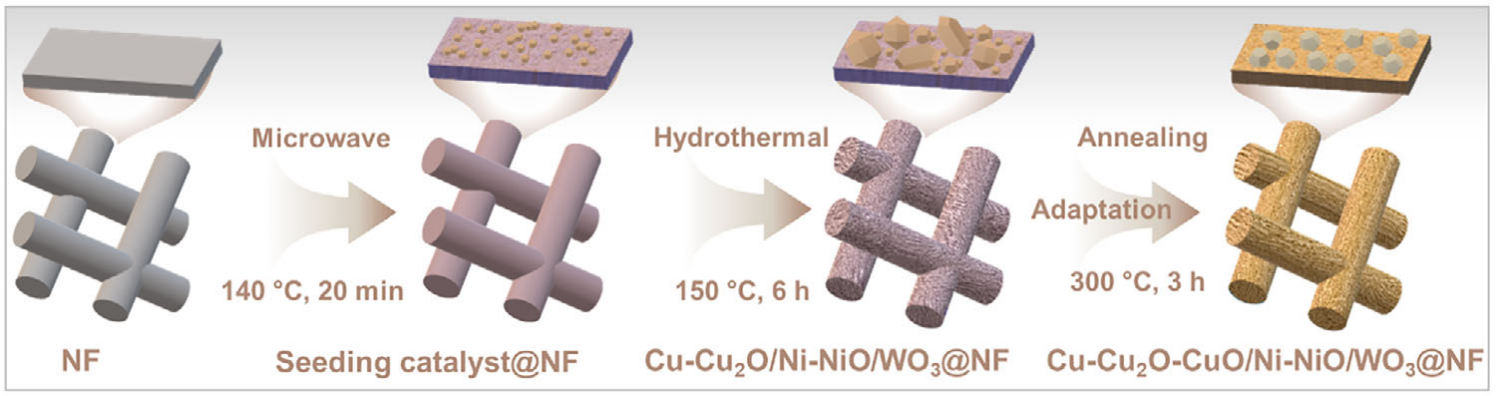
Schematic representation of the materials design and fabrication strategy for the development of a tri‐component tandem electrocatalyst. Sequential microwave‐assisted and hydrothermal deposition of Cu, Ni, and W components onto Ni foam (**NF**) electrode delivers an adaptive electrode system tailored for coupled NO_3_^−^RR (by Cu‐Cu_2_O/Ni‐NiO/WO_3_@NF, **E3**) and GOR (by Cu‐Cu_2_O‐CuO/Ni‐NiO/WO_3_@NF, **E6**). For an illustration of the coupled system, see Figure 6f.

Herein, we elaborately propose a generalist tri‐component electrocatalyst model (designated Cu‐Cu_2_O/Ni‐NiO/WO_3_@NF), featuring Cu‐Cu_2_O as a high‐performing NO_3_^−^‐to‐NO_2_^−^ catalyst, Ni‐NiO for enhanced *H supply, as well as WO_3_ as a tandem reaction balancer/regulator for the deep hydrogenation of generated N‐containing intermediates. Under pulsed electrolysis conditions, high Faradaic efficiency (FE, 97.2%), outstanding NH_3_ yield rate (43.87 mg h^−1^ cm^−2^) and competitive operational durability (>14 cycles) are collectively observed. Online differential electrochemical mass spectrometry (DEMS) analysis elucidates the key intermediates and catalytic reaction paths under operation. Complementary density functional theory (DFT) calculations further illuminate the reaction mechanisms mediated by the tri‐component tandem catalyst, aligning with and rationalizing the catalytic performance. Furthermore, we also provide a contributive anodic reaction for the NO_3_^−^RR‐to‐NH_3_, specifically through the GOR, validated using a Cu‐Cu_2_O‐CuO/Ni‐NiO/WO_3_@NF electrode thermally adapted from the Cu‐Cu_2_O/Ni‐NiO/WO_3_@NF. This work formulates new guidelines and reliable strategies encompassing adaptive catalyst design, technological engineering, and system integration, thereby advancing sustainable NH_3_ production and biomass‐derived electrochemical refining (Scheme 1).

## 2 Results and Discussion

### 2.1 Electrocatalyst Preparation and Characterization

Here, we present a bottom‐up materials design strategy utilizing a sequential microwave‐assisted and hydrothermal deposition approach of the molecular precursors Cu(NO_3_)_2_·3H_2_O (0.026 mM) and K_8_[SiW_11_O_39_]·13H_2_O (0.027 mM) with a bare Ni foam (**NF**), which serves dual roles as both an electrode support and a source of Ni ions (Figure S1). Of note, the microwave‐assisted deposition method alone leads to the formation of a thin seeding catalyst layer on the **NF** (Figure S2), resulting in Electrode 1 (**E1**). In contrast, exclusive use of the hydrothermal method induces microparticle aggregation across the electrode surface (Figure S3), giving Electrode 2 (**E2**). By sequentially integrating both methods, we achieve a hierarchically structured composite electrode after an electrochemical pre‐reduction process, designated Electrode 3 (**E3**, Cu‐Cu_2_O/Ni‐NiO/WO_3_@NF). This synergistic two‐step wet‐chemical approach is deemed to enhance structural integration. Detailed preparation procedures are provided in the Supporting Information (SI: Sections 3.1–3.3 and Table S1).

**FIGURE 1:**
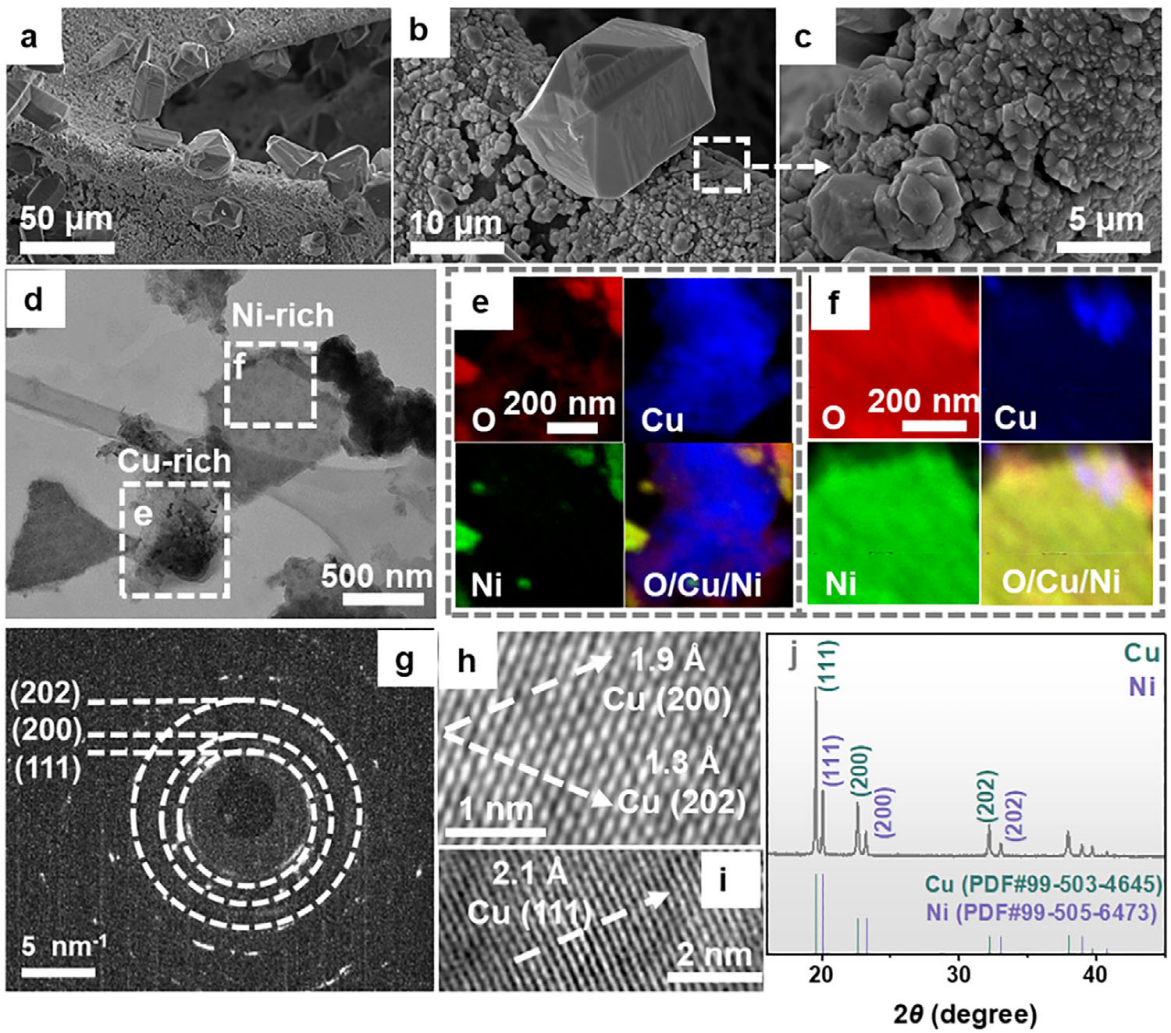
Structural and chemical characterization of **E3**. (a–c) SEM images of **E3** bulk electrode. (d) TEM image of pure catalyst peeled off from **E3**. (e and f) EDX elemental mappings of Cu‐rich and Ni‐rich areas. (g) Selected area electron diffraction of the Cu‐rich particle (Figure S8), showing diffraction spots matching reflections of polycrystalline Cu in (111), (200), and (202) planes. (h and i) Corresponding HRTEM images. (j) pXRD data of catalyst recovered from **E3**. Recovery of the pure catalysts from the **NF** electrode was achieved by prolonged sonication in ethanol.

As illustrated in scanning electron microscopy (SEM, Figure 1a–c and S4) and transmission electron microscopy (TEM, Figures 1d and S5), the hierarchical surface of **E3** comprises sparsely distributed large polyhedrons (diameter: 20–40 µm) supported on a base layer of densely interconnected small polygonal particles (diameter: 500 nm–3 µm). This gradient microstructure has been reported to enhance reactant adsorption and optimize the confinement of reaction intermediates [61, 62], thus contributing to the NO_3_^−^RR performance in this work. Furthermore, energy‐dispersive X‐ray spectroscopy (EDX) elemental mapping of bulk **E3** from SEM (Figure S6) confirms the presence of O, Cu, Ni, and W within the catalyst layer (atomic ratio O:Cu:Ni:W = 3.3:80.4:15.3:1.0), exhibiting a hierarchical architecture characterized by large Cu‐rich polyhedrons decorated with very sparsely dispersed W oxides on the Ni support. Complementary EDX elemental mapping of detached pure catalyst from scanning TEM (STEM) reveals the coexistence of individual Cu‐rich and Ni‐rich small polygonal particles (Figures 1d–f and S7), while W was undetectable due to its low loading, consistent with the SEM‐EDX result (Figure S6). Correspondingly, TEM analysis, including local selected area electron diffraction (SAED: Figures 1h and S8–S10) and high‐resolution TEM (HRTEM; Figures 1h,i and S10), confirms that **E3** primarily comprises polycrystalline Cu and Ni phases, aligning with the powder X‐ray diffraction (pXRD) pattern (Figure 1k). Specifically, the SAED pattern presents concentric diffraction rings corresponding to the (111), (200), and (202) planes of Cu (Figure 1g). These diffraction features are corroborated by the associated lattice fringe spacings of 2.1, 1.9, and 1.3 Å, respectively (Figures 1h,i and S9). Notably, the (111) plane of Ni, characterized by a lattice fringe spacing of 2.1 Å, is more prominently observed in the measured region (Figure S10).

**FIGURE 2:**
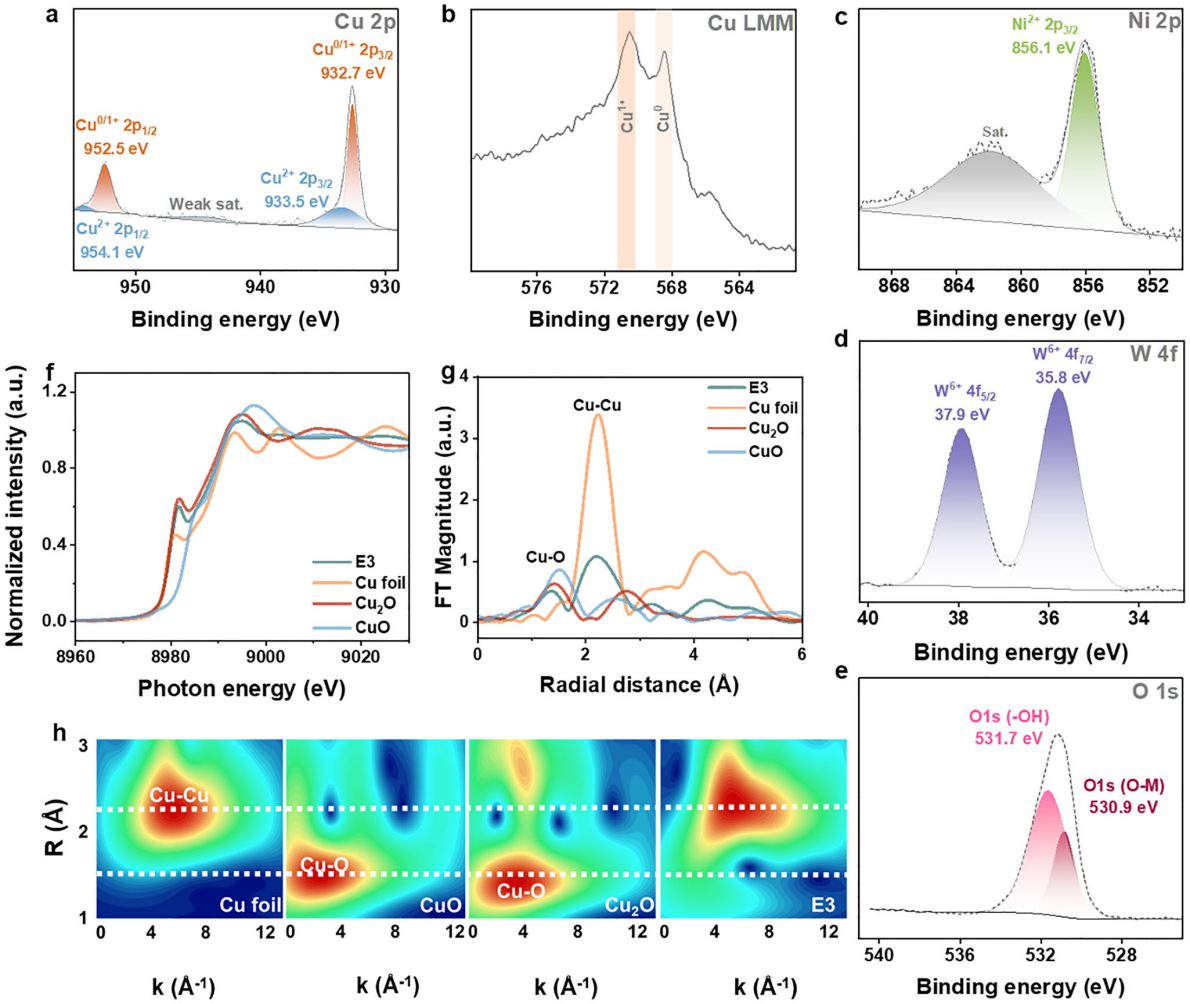
Deconvoluted XPS spectra for (a) Cu 2p, (b) Cu LMM, (c) Ni 2p, (d) W 4f, and (e) O 1s of **E3**. Cu K‐edge (f) XANES and (g) EXAFS spectra of bulk **E3** and reference compounds. (h) Wavelet transform analysis of EXAFS spectra of Cu K‐edge for Cu foil, CuO, Cu_2_O, and **E3** catalyst. Recovery of the pure catalysts from the **NF** electrode was achieved by prolonged sonication in ethanol.

Further, X‐ray photoelectron spectroscopy (XPS) was conducted to investigate the chemical composition and the surface valence states of **E3**. The XPS survey spectrum verifies the presence of all anticipated elements in the catalyst, namely Cu, Ni, W, and O (Figure S11). Specifically, detailed analysis of the Cu 2p region (Figure 2a) and Cu LMM Auger spectrum (Figure 2b) reveals the co‐existence of metallic Cu and Cu^1+^, along with a minor contribution from Cu^2+^‐containing phases, likely originating from the formation of a thin surface oxide layer upon air exposure [63, 64]. The Ni 2p region (Figure 2c) indicates the presence of sole Ni^2+^ [65], attributed to the commonly observed surface oxidation of **NF** support, which likely explains the absence of metallic Ni species, as previously corroborated by pXRD (Figure 1j) and TEM analyses (Figure S10). The W 4f spectrum unambiguously identifies W^6+^ species (Figure 2d) [63]. Additionally, the deconvoluted O 1s spectrum further validates the presence of metal─oxygen bonds (M─O, including Cu─O, Ni─O, and W─O), alongside hydroxyl groups (O─H, Figure 2e) [14]. The presence of the aforementioned metal oxides was further corroborated by ATR‐FTIR spectroscopy (Figure S12). Explicitly, the ATR‐FTIR spectrum exhibited peaks at 614 and 508 cm^−1^, corresponding to the Cu─O bond [14]. Additionally, the Ni─O vibrational mode [66] was detected at 690 cm^−1^. The characteristic bond associated with W═O was observed at 954 cm^−1^, while characteristic peaks at 891 and 782 cm^−1^ were assigned to W─O─W bonding [67]. In addition, ATR‐FTIR spectroscopy identified O─H vibrations and thermogravimetric analysis (TGA) indicated approximately 1.05 wt% water of hydration (Figure S13). As measured by inductively coupled plasma optical emission spectroscopy (ICP‐OES), the atomic ratio of Cu:Ni:W in the pure catalyst peeled off from **E3** (by prolonged sonication in ethanol) is determined to be 7.2:8.1:1.0 (Table S2). The deviation between the ICP‐OES and EDX results (Figure S6) is attributed to the bulk‐sensitive nature of ICP‐OES in contrast to the surface‐localized and spatially resolved characteristics of EDX mapping.

Next, X‐ray absorption spectroscopy (XAS), including X‐ray absorption near‐edge spectroscopy (XANES), extended X‐ray absorption fine structure (EXAFS), and wavelet transform (WT) analysis, was carried out to gain insight into the oxidation state and coordination environment of Cu, Ni, and W in **E3**.

Explicitly, the Cu K‐edge XANES spectrum (Figure 2f) shows that the pre‐edge position overlaps with both metallic Cu and Cu_2_O references, indicating the coexistence of Cu^0^ and Cu^1+^ species. Furthermore, analysis of the EXAFS data in both k‐space (Figure S14a) and R‐space (Figure 2g) reveals the presence of Cu‐O and Cu‐Cu coordination, further supporting the assignment of mixed valences in **E3**. Quantitative EXAFS fitting reveals dominant Cu‐Cu coordination (CN ≈ 8.6 at 2.58 Å), accompanied by a minor Cu‐O shell (CN ≈ 0.05 at 1.89 Å) and Cu‐Ni interactions (CN ≈ 1.8 at 2.52 Å) (Figure S15a and Table S3), which may indicate potential atomic‐level interactions between Cu and Ni for partial bimetallic character [68]. This is further corroborated by the WT‐EXAFS analysis (Figure 2h), in which the spectrum shows features of predominant intensity matching metallic Cu, as well as distinct contributions at positions that are characteristic of Cu‐O coordination.

The Ni K‐edge XANES spectrum (Figure S16a) shows a slight red shift in the absorption edge compared to the Ni foil. While this shift might suggest an altered electronic environment. EXAFS data (Figure S14b) and fitting (Figure S15b and Table S4) confirm Ni‐Ni and Ni‐Cu coordination features (Ni‐Ni, CN ≈ 1.7 at 2.51 Å; Ni‐Cu, CN ≈ 8.2 at 2.52 Å). This observation indicates that Ni is predominantly present in its metallic state, with additional evidence of local Ni‐Cu coordination. The WT (Figure S16b,c) further supports this assignment and is consistent with the bulk‐sensitive XRD (Figure 1j) and TEM analyses (Figure S10). In contrast, the Ni^2+^ signal detected by surface‐sensitive XPS (Figure 2d) can be attributed to a thin oxidized surface layer formed upon air exposure, rather than representing the bulk environment. Furthermore, the W L_3_‐edge XANES spectrum (Figure S17a) reveals a strong white line and an absorption edge position consistent with WO_3_, identifying the W^6+^ oxidation state. EXAFS and WT analyses (Figures S14c and S17b,c) show exclusively W‐O coordination pathways with no evidence of W‐W contributions, ruling out metallic or sub‐stoichiometric W species.

To investigate the influence of the wet‐chemical deposition step on the composition of the resulting electrodes obtained, we conducted SEM‐EDX mapping and XPS analyses on **E1**, prepared solely via microwave deposition (Figures S18, S19), and **E2**, prepared solely via hydrothermal deposition (Figures S20, S21). As shown, both **E1** and **E2** exhibit identical elemental compositions and chemical valence states, consistent with those observed for **E3**, indicating that both deposition methods effectively deliver the intended functional composition but with varying catalyst growth and morphology (Figures S2, S3). To further elucidate the individual roles of the Cu(NO_3_)_2_·3H_2_O and K_8_[SiW_11_O_39_]·13H_2_O precursors in the synthesis, we performed identical deposition in the respective single precursor component (Table S1), leading to Electrode 4 (**E4**) and Electrode 5 (**E5**). Note that, in the absence of either precursor, no microparticles were deposited on the **NF** support (Figures S22–S25), strongly suggesting that a synergistic interaction between both precursors is essential for the successful formation of the hierarchically structured surface characteristic of **E3**. XPS analysis of **E4** revealed the surface presence of Cu^2+^, Ni^2+^ (from NiO, Ni(OH)_2_) and Ni^0^ species, along with oxygen─metal bonds and hydroxyl groups (Figure S26). These findings highlight a distinct chemical composition of **E4** compared to **E3**, resulting from the absence of K_8_[SiW_11_O_39_]·13H_2_O precursors during synthesis. In contrast, **E5** exhibits an elemental composition similar to that of **E3** in terms of Ni, W, and O (Figure S27). Furthermore, relative to **E3** prepared with a Cu^2+^:[SiW_11_O_39_]^8−^ ratio of 1:1, the atomic composition of the resulting electrodes can be effectively tuned by varying the Cu^2+^:[SiW_11_O_39_]^8−^ ratio in the precursor solution (e.g., 2:1 and 1:2), leading to systematic compositional and morphological modulation (Table S5 and Figures S28, S29).

Overall, these results suggest that each single precursor component exerts a unique influence on the final composition of the deposited catalyst. All the resulting controls were used as references for comparing the NO_3_^−^RR performance of **E3**.

### 2.2 Electrocatalytic NO_3_^−^RR‐to‐NH_3_ Performance in Model Electrolytes

In the next step, we evaluated the electrocatalytic performance of the composite electrodes for NO_3_^−^RR‐to‐NH_3_ utilizing a standard H‐cell with a three‐electrode configuration. The measurements were conducted under ambient conditions in an Ar‐saturated model electrolyte of 1 M NaOH aqueous solution containing 0.1 M NaNO_3_ (pH 13.8). All potentials reported in this study were referenced to the reversible hydrogen electrode (RHE).

**FIGURE 3:**
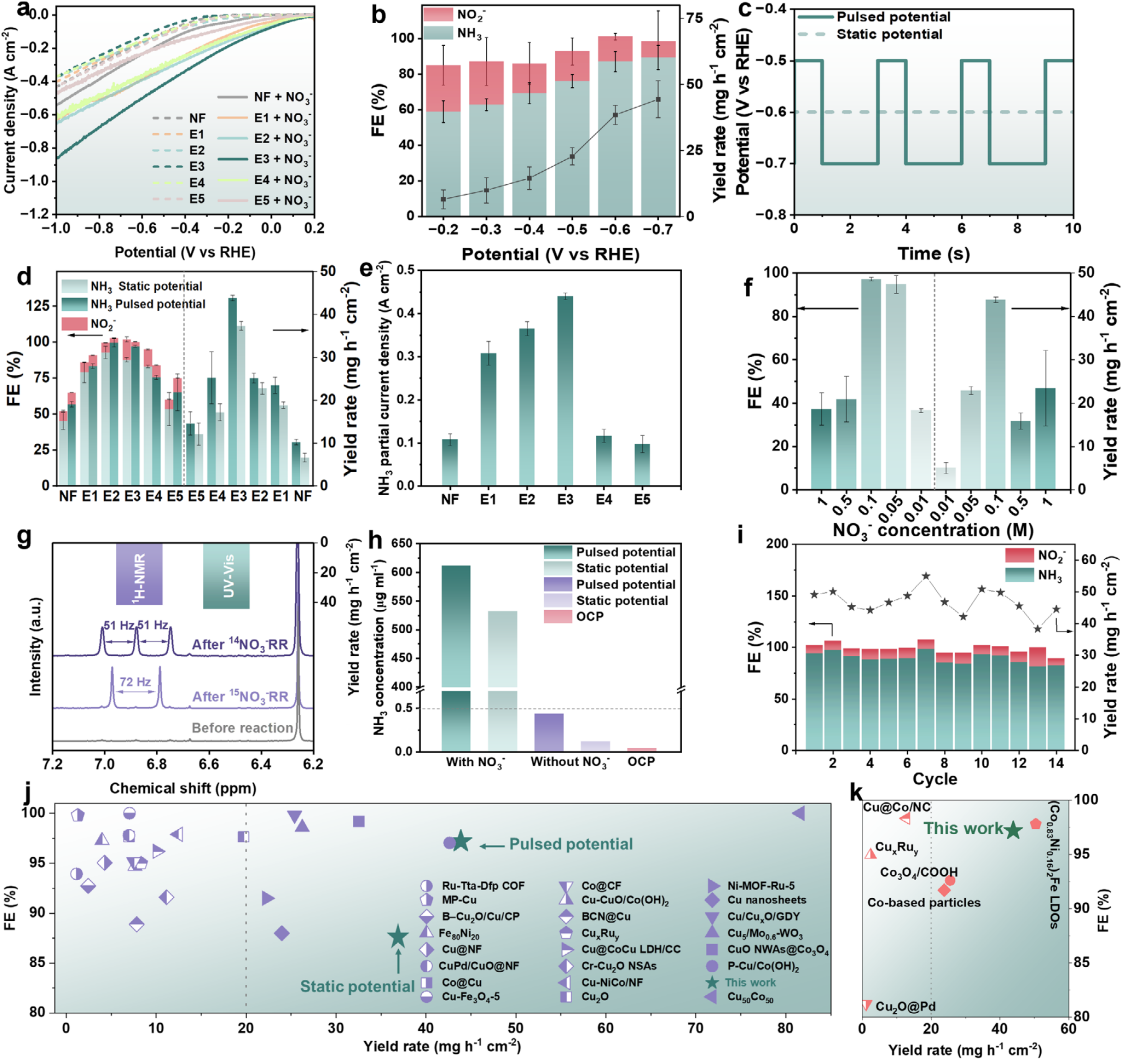
NO_3_^−^RR‐to‐NH_3_ electroreduction performance evaluation. (a) LSV curves of **NF**, **E1–E5** in 1 M NaOH with (solid line) and without 0.1 M NaNO_3_ (dashed line), scan rate: 5 mV s^−1^, without iR correction. (b) Bar diagram representing NH_3_ yield rate, NH_3_ FE, and NO_2_^−^ FE of **E3** at different static potentials. (c) Comparison of the static potential and the pulse potential pattern employed. (d) NH_3_ yield rate, NH_3_ FE and NO_2_^−^ FE of **NF**, **E1–E5** under static (−0.6 V vs. RHE) and pulsed (−0.5 V vs. RHE for 1 s followed by −0.7 V vs. RHE for 2 s) electrolysis. (e) NH_3_ partial current density over **NF**, **E1–E5** at static −0.6 V vs. RHE potential. (f) NH_3_ yield rate and NH_3_ FE of **E3** at different NO_3_^−^ concentrations. (g) ^1^H NMR spectra of the electrolyte after NO_3_^−^RR over **E3** under pulsed electrolysis using ^15^NO_3_^−^ and ^14^NO_3_^−^ as the nitrogen source. Inset: NH_3_ yield rate determined from UV–vis and ^1^H NMR methods. (h) NH_3_ concentration of **E3** under pulsed electrolysis with/without NO_3_^−^, and at OCP with NO_3_^−^, respectively. (i) Stability test of **E3** in 1 M NaOH + 0.1 M NaNO_3_ under pulsed electrolysis. Comparison of NH_3_ yield rate and FE of **E3** with recently (j) developed Ni, Cu, and WO_3_‐based NO_3_^−^RR catalysts under potentiostatic conditions and (k) reported NO_3_^−^RR catalysts under pulsed electrolysis conditions in alkaline media.

The electrocatalytic activity of the **NF**, **E1**–**E5** composite electrodes was initially assessed through linear sweep voltammetry (LSV) across a potential range from 0.2 to −1.0 V versus RHE. As shown in Figure 3a, in the absence of NO_3_^−^, the HER activity of **NF**, **E1**, **E2**, **E4**, and **E5** was nearly identical, whereas **E3** exhibited the lowest performance metric. Of note, the introduction of 0.1 M NO_3_^−^ resulted in a considerable enhancement in the current density for **E1**, **E2**, and **E4** (Figure S30), attributed to the NO_3_^−^RR facilitated by the presence of Cu and Ni components in these electrodes. In contrast, bare **NF** displayed only a moderate increase, while the Ni‐ and W‐containing **E5** exhibited minimal NO_3_^−^RR activity, particularly at more negative potentials (Figure S30). This limited response is likely due to inadequate NO_3_^−^ adsorption and deoxygenation for both bare Ni foam and Cu‐component‐free **E5**. Meanwhile, the presence of W component in **E5** appears to dynamically modulate the *H utilization, resulting in an electrocatalytic profile that more closely resembles HER behavior.

Remarkably, the tri‐component **E3** showed the most pronounced separation between NO_3_^−^RR and HER curves, along with consistently higher current densities across the entire potential range compared to **NF**, the single‐deposition‐step controls (**E1** and **E2**), as well as the single‐precursor‐deposition controls (**E4** and **E5**). Notably, **E3** achieved a peak current density of −0.86 A cm^−2^ at −1.0 V versus RHE, significantly outperforming **E1** (−0.63 A cm^−2^), **E2** (−0.65 A cm^−2^), **E4** (−0.61 A cm^−2^), and **E5** (−0.46 A cm^−2^), as well as the screened Cu^2+^:[SiW_11_O_39_]^8−^ ratio counterparts (−0.64 A cm^−2^ for 2:1, −0.73 A cm^−2^ for 1:2, Figure S31). This superior performance underscores the advantage of the sequential deposition strategy using an optimized precursor recipe combined with a strategic pre‐reduction process (Figure S32a), which integrates Cu, Ni, and W components into well‐defined initial catalytic conditions [69] (e.g., modified surface oxidation state and crystal structure, Figure S32b). Such integration enables a well‐matched tandem reaction rate between NO_3_^−^ deoxygenation and *H generation/utilization for deep hydrogenation, thereby promoting efficient NO_3_^−^RR‐to‐NH_3_ conversion. In addition, the electrochemically active surface area (ECSA) is a key parameter that represents the effective surface area of the electrode capable of participating in electrochemical reactions, which can be determined by evaluating the double‐layer capacitance (*C*_dl_) of the electrode (Figure S33). As shown (Figure S34), **E3** demonstrates the highest *C*_dl_ and ECSA when compared to both **NF** and control electrodes (**E1**, **E2**, **E4**, and **E5**), providing further evidence of the superior intrinsic catalytic activity of **E3** toward NO_3_^−^RR.

Next, triplicate chronoamperometry (CA) measurements were systematically performed on **E3** at various applied potentials for 1 h each (Figure S35), and the UV–vis indophenol blue method was employed to quantify the primary product, NH_3_, as well as the potential byproduct, NO_2_^−^ (with the corresponding calibration curves depicted in Figure S36). The obtained FEs for NH_3_ and NO_2_^−^, as well as the yield rates of NH_3,_ were displayed as a function of the applied potential (Figure 3b). Notably, based on the validated NH_3_ quantification protocol (Figure S37), the error bars primarily reflect minor electrode‐to‐electrode variations in nanostructure and active‐site density, which are commonly observed for nanostructured electrocatalysts [47, 70, 71]. Explicitly, the results revealed that both the FE and the yield rate of NH_3_ increased with more negative potentials, reaching a maximum value of 44 mg h^−1^ cm^−2^ at −0.7 V versus RHE. Notably, the highest combined FE for NH_3_ (87.0%) main product and NO_2_^−^ (14.1%) byproduct was observed at −0.6 V versus RHE, with the sum slightly exceeding 100%. This suggests effective suppression of the competing HER under these conditions. In comparison, the control electrodes (Figures 3d and S38, S39) exhibited lower NH_3_ FEs of 45.2%, 79.1%, 52.6%, and 57.8% for **NF**, **E1**, **E4**, and **E5**, respectively. Interestingly, **E2** achieved a relatively high NH_3_ FE of 92.9%. To further evaluate catalytic performance, the NH_3_ partial current density at −0.6 V versus RHE was derived (Figure 3e), confirming that **E3** exhibits the highest NO_3_^−^RR‐to‐NH_3_ activity among all tested electrodes.

Subsequently, a pulsed potential strategy (−0.5 V vs. RHE for 1 s followed by −0.7 V vs. RHE for 2 s, Figure 3c) was implemented for all electrodes. For **NF**, **E1**, **E2**, **E3**, and **E5**, both the NH_3_ FEs and yield rates were enhanced (Figure 3d), reflecting the trends consistent with those observed under static potential conditions (with the corresponding triplicate CA data presented in Figures S38, S39). In particular, for **E3**, NH_3_ FE increased from 87.0% to 97.1%, accompanied by a decrease in NO_2_^−^ FE reduced from 14.1% to 3.1%, and a concurrent enhancement in the yield rate from 37.36 mg h^−1^ cm^−2^ to 43.87 mg h^−1^ cm^−2^. Although the NH_3_ FE of **E3** under pulsed potentials remained slightly lower than that of **E2**, it delivered the highest NH_3_ yield rate among all tested electrodes. Specifically, **E3** outperformed **NF**, **E1**, **E2**, **E4**, and **E5** by factors of 4.3, 1.9, 1.7, 1.7, and 3.0, respectively. Moreover, the detailed analysis on **E3** over the triplicate CA tests (Run 1–Run 3, Figure S40) reveals an initial activation process emerging in Run 2, followed by a marginal performance decline in Run 3. This behavior indicates minor, electrode‐associated performance fluctuations, likely arising from subtle structural changes (Figure S41). Interestingly, under pulsed conditions, **E4** exhibited a decrease in FE despite an increased NH_3_ yield rate. This behavior is likely due to the competitive interplay between NO_3_^−^RR and HER, resulting from the imbalance in the reaction kinetics of NO_3_^−^ deoxygenation and *H regulation in the W‐component‐free system.

Next, to verify the contribution of *H species generated via H_2_O dissociation, *tert*‐butanol (TBA) was employed as a *H quencher during NO_3_^−^RR‐to‐NH_3_ [72, 73]. As shown, the addition of TBA to the electrolyte led to a notable suppression of the cathodic current density for **E3** (Figure S42). Correspondingly, the NH_3_ yield decreased from 43.87 to 23.93 mg h^−1^ cm^−2^, and the FE dropped from 97.1% to 81.2%, highlighting the critical role of *H in the electrochemical conversion of NO_3_^−^ to NH_3_.

To assess the applicability of **E3** for treating wastewater from diverse sources, the NO_3_^−^RR performance under pulsed potential conditions was systematically evaluated across a suite of NO_3_^−^ concentrations. Notably, **E3** exhibits a high peak current density of 0.49 A cm^−2^ even at a low NO_3_^−^ concentration of 0.01 M. This current density progressively increases with rising NO_3_^−^ concentration, reaching an industrially relevant level of 1.26 A cm^−2^ at 1 M NO_3_^−^ (Figure S43). These results highlight the strong potential of **E3** for efficient NO_3_^−^ removal from wastewater with varying nitrate loadings. In addition, increasing the NO_3_^−^ concentration up to 0.1 M enhances both the NH_3_ yield rate and FE (Figure 3f). However, further increases in NO_3_^−^ concentration result in a noticeable decline in performance metrics (Figures 3f and S44). This decline can be attributed to insufficient *H supply and the blocking of catalytic sites due to the undesired accumulation of N‐containing intermediates.

Moreover, ^1^H nuclear magnetic resonance (NMR) spectroscopy was employed as an additional verification method for NH_3_ product quantification (with the calibration curves shown in Figure S45). The yield rate obtained from this is found to be mostly consistent with that from UV–vis indophenol blue method, thereby demonstrating the high reliability of the quantitative approaches utilized (Figures 3g and S37). Furthermore, an isotope labeling experiment was conducted to confirm the origin of the N element in the NH_3_ product. As illustrated in Figure 3g, no ammonium peak was detected prior to the reaction. Upon feeding ^15^NO_3_^−^ as the N source, a characteristic doublet with equal intensity and a coupling constant of 72 Hz appeared for ^15^NH_4_^+^ following the ^15^NO_3_^−^RR [74]. In contrast, when ^14^NO_3_^−^ was used, a triplet of equal distance and intensity for ^14^NH_4_^+^ with a coupling constant of 51 Hz was observed after ^14^NO_3_^−^RR [74]. These results unequivocally confirm that the NH_3_ produced originates from the reduction of NO_3_^−^ in the electrolyte instead of other nitrogen sources. Aside from that, only trace amounts (<0.5 µg mL^−1^) of NH_3_ were detected either in the absence of electrolysis or electrolysis in the absence of NO_3_^−^ (Figures 3h and S46), further corroborating that NH_3_ is exclusively derived from NO_3_^−^RR in the electrolyte employed. Furthermore, 14 consecutive cycles of 1 h each under pulsed potential conditions were performed to investigate the electrochemical stability of **E3**. As displayed in Figures 3i and S47, **E3** demonstrates a sustained current density, NH_3_ yield rate and FE over 14 cycles without noticeable degradation, indicative of robust stability for NO_3_^−^RR‐to‐NH_3_.

Post‐catalytic XPS data after 1 h CA (Figure S48) indicate identical chemical nature and unchanged valence states of Cu, W, and Ni in comparison to the as‐prepared **E3**, highlighting its robustness. Furthermore, the post‐catalytic XRD pattern after 14 cycles (Figure S49) shows no detectable phase change or peak broadening, indicating preserved crystallographic integrity. SEM images acquired after 14 cycles (Figure S50) reveal a possible restructuring of the sparsely distributed large polyhedrons, leading to a comparable morphology and microstructure of the base layer of densely interconnected small polygonal particles of fresh **E3** (Figure 1c). In addition, ICP‐OES analysis of the electrolyte collected after cycles 1, 7, and 14 (Table S6) was performed to assess possible metal dissolution during prolonged NO_3_^−^RR operation. Only trace amounts of Cu were detected, with the concentration decreasing upon continued cycling, indicating that Cu leaching is minimal and largely confined to the initial stage of electrolysis. Notably, Ni was not detected at any cycle within the detection limits of ICP‐OES. These observations further corroborate the compositional stability of **E3** under long‐term NO_3_^−^RR conditions.

Collectively, these findings highlight the outstanding catalytic performance of the proposed tri‐component Cu‐Cu_2_O/Ni‐NiO/WO_3_@NF electrocatalyst for NO_3_^−^RR‐to‐NH_3_ in alkaline media. As illustrated in Figure 3j and summarized in Table S7, while several recently reported Ni‐, Cu‐, and WO_3_‐based catalysts have achieved FEs exceeding 90% in alkaline media, they generally exhibit modest yield rates, typically below 20 mg h^−1^ cm^−2^ under comparable conditions (0.005–1 M NO_3_^−^ in 0.1–1 M alkaline electrolyte). In sharp contrast, **E3** delivers markedly superior performance, particularly under pulsed potential conditions. Remarkably, it achieves a record‐high FE of 97.1%, accompanied by a substantially enhanced yield rate of 43.87 mg h^−1^ cm^−2^ and competitive cycling durability, surpassing the performance of recently reported electrocatalysts under comparable pulsed electrolysis conditions (Figure 3k and Table S8). These metrics collectively underscore its superb catalytic efficiency for NO_3_^−^RR‐to‐NH_3_ conversion.

### 2.3 Electrocatalytic Mechanistic Studies

**FIGURE 4:**
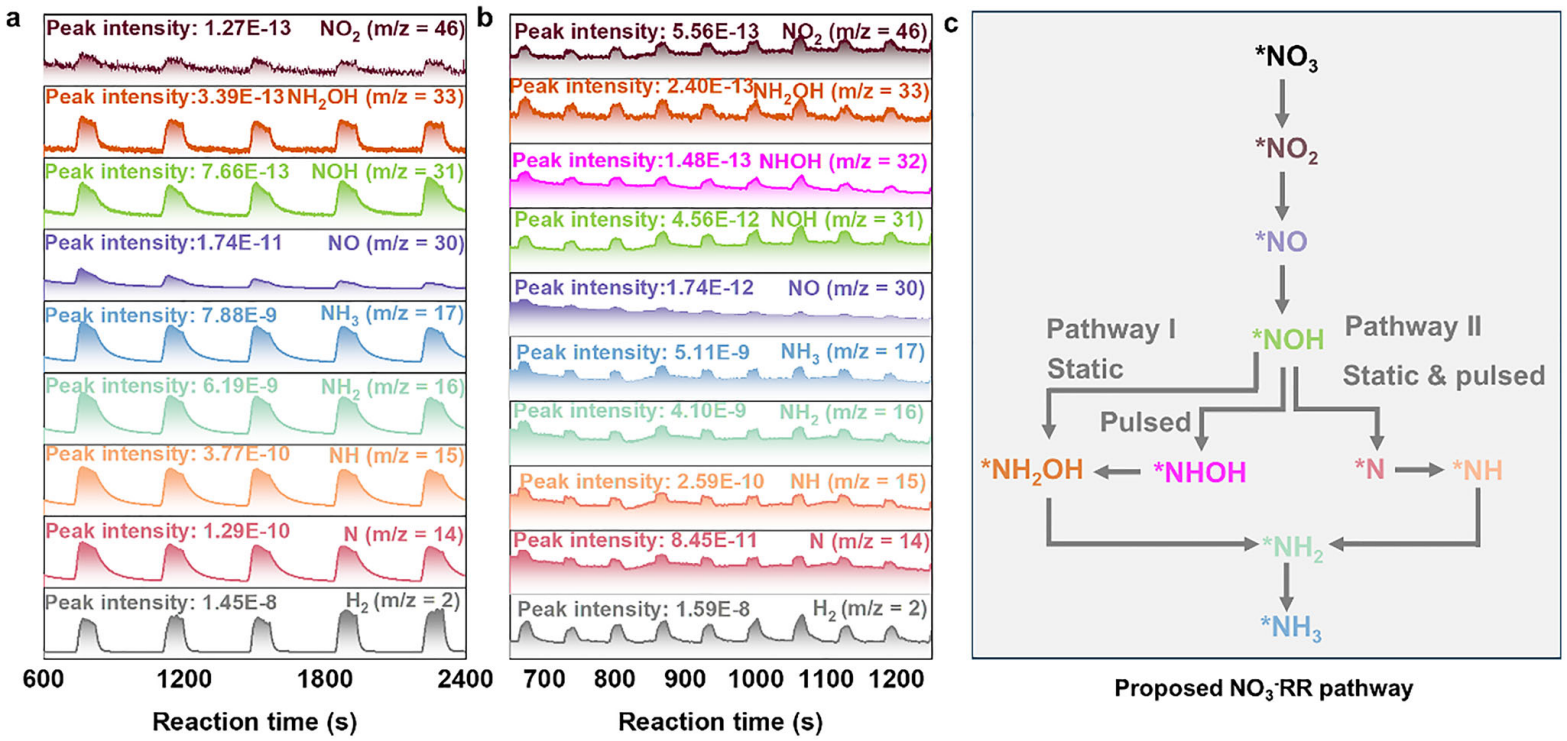
DEMS data of **E3** under (a) static (−0.6 V vs. RHE) and (b) pulsed (−0.5 V vs. RHE for 1 s followed by −0.7 V vs. RHE for 2 s) electrolysis. (c) Proposed NO_3_^−^RR pathways of **E3**.

To elucidate the catalytic mechanism at the molecular level, online DEMS was conducted over multiple cycles to decipher the reaction intermediates and pathways during the NO_3_^−^RR. Notably, any contribution of anodic O_2_ to the detected signal was excluded through a deliberately designed cell configuration (see SI for details). As displayed in Figure 4a, under static electrolysis conditions, the detected signals at mass‐to‐charge ratios (*m*/*z*) of 46, 33, 31, 30, 17, 16, 15, 14, and 2 correspond to NO_2_, NH_2_OH, NOH, NO, NH_3_, NH_2_, NH, N, and H_2_, respectively. It is worth noting that, due to the high applied potential (−0.6 V vs. RHE), HER is likely to occur as a side reaction. Moreover, the absence of an *m*/*z* of 28 signal indicates that nitrogen evolution (NO_3_^−^RR‐to‐N_2_) is effectively suppressed under these conditions.

Among the detected species, signals for NH_3_ and NH_2_ are notably more intense than those of other N‐containing intermediates, suggesting that NH_3_ and NH_2_ are the predominant gaseous products of NO_3_^−^RR during short reaction durations. Based on these observations, a plausible reaction pathway involves sequential deoxygenation and hydrogenation steps (Figure 4c), proceeding via *NO_3_ → *NO_2_ → *NO, followed by *NO → *NOH → *NH_2_OH → *NH_2_ → *NH_3_ (Pathway I), or *NO → *NOH → *N → *NH → *NH_2_ → *NH_3_ (Pathway II). In contrast, under pulsed electrolysis, the intensity of the *NOH signal is approximately one order of magnitude higher than under static conditions, evidencing an accelerated *NO → *NOH step. Concurrently, a new signal at *m*/*z* of 32 emerges, attributable to the *NHOH intermediate (Figure 4b). These features demonstrate that pulsed bias modulates intermediate formation and redirects the reaction pathway, consistent with previous reports [50, 75, 76, 77]. Accordingly, under pulsed conditions, the hydrogenation sequence may proceed via *NO → *NOH → *NHOH → *NH_2_OH → *NH_2_ → *NH_3_ (Pathway I), or *NO → *NOH → *N →*NH → *NH_2_ → *NH_3_ (Pathway II), with the preferred pathway further elucidated by subsequent DFT calculations.

To further clarify the role of individual components, DEMS measurements were conducted for single‐precursor‐derived control catalysts (**E4** and **E5**) under pulsed electrolysis (Figure S51). Although the *NHOH intermediate is detected for both **E4** and **E5**, highlighting the general effectiveness of pulsed electrolysis in regulating key intermediates, distinct concentration differences from **E3** are evident, underscoring pronounced compositional effects. Notably, the appearance of an *m*/*z* = 28 signal evidences the activation of a competing NO_3_^−^RR‐to‐N_2_ pathway on **E4** and **E5**. In parallel, the *NOH signal intensity is approximately one order of magnitude lower than that observed for **E3**, indicating a substantially suppressed *NO → *NOH hydrogenation step. These spectroscopic features collectively rationalize the inferior NO_3_^−^RR‐to‐NH_3_ performance of **E4** and **E5** (Figure 3d). In particular, **E4** exhibits pronounced *NO accumulation, which likely originates from a kinetic mismatch between deoxygenation and hydrogenation processes in the absence of the W component, thereby favoring intermediate buildup and parasitic pathways over sustained hydrogenation toward NH_3_.

### 2.4 Theoretical Calculations

To disclose the synergistic role of the tri‐component tandem electrocatalyst in the NO_3_^−^RR‐to‐NH_3_, DFT calculations were performed to provide atomic‐level insights into the catalytic mechanism. Building on the aforementioned physicochemical characterization data (e.g., TEM, XRD, XPS, and XAS), model structures (Figures S52–S56), including crystalline Cu and Ni, amorphous Cu_2_O supported on Cu (Cu‐Cu_2_O), and WO_3_ supported on Ni (Ni‐WO_3_) were constructed based on ab initio molecular dynamics (AIMD) calculation and the melt‐quenching method. Of note, according to Wulff construction analyses (Figure S56) for the crystalline Cu and Ni, the (111) planes are thermodynamically most favorable, comprising 77% and 71% of the total exposure surface area, respectively [78]. Consequently, Cu (111) and Ni (111) model surfaces are simulated as substrates in this section.

**FIGURE 5:**
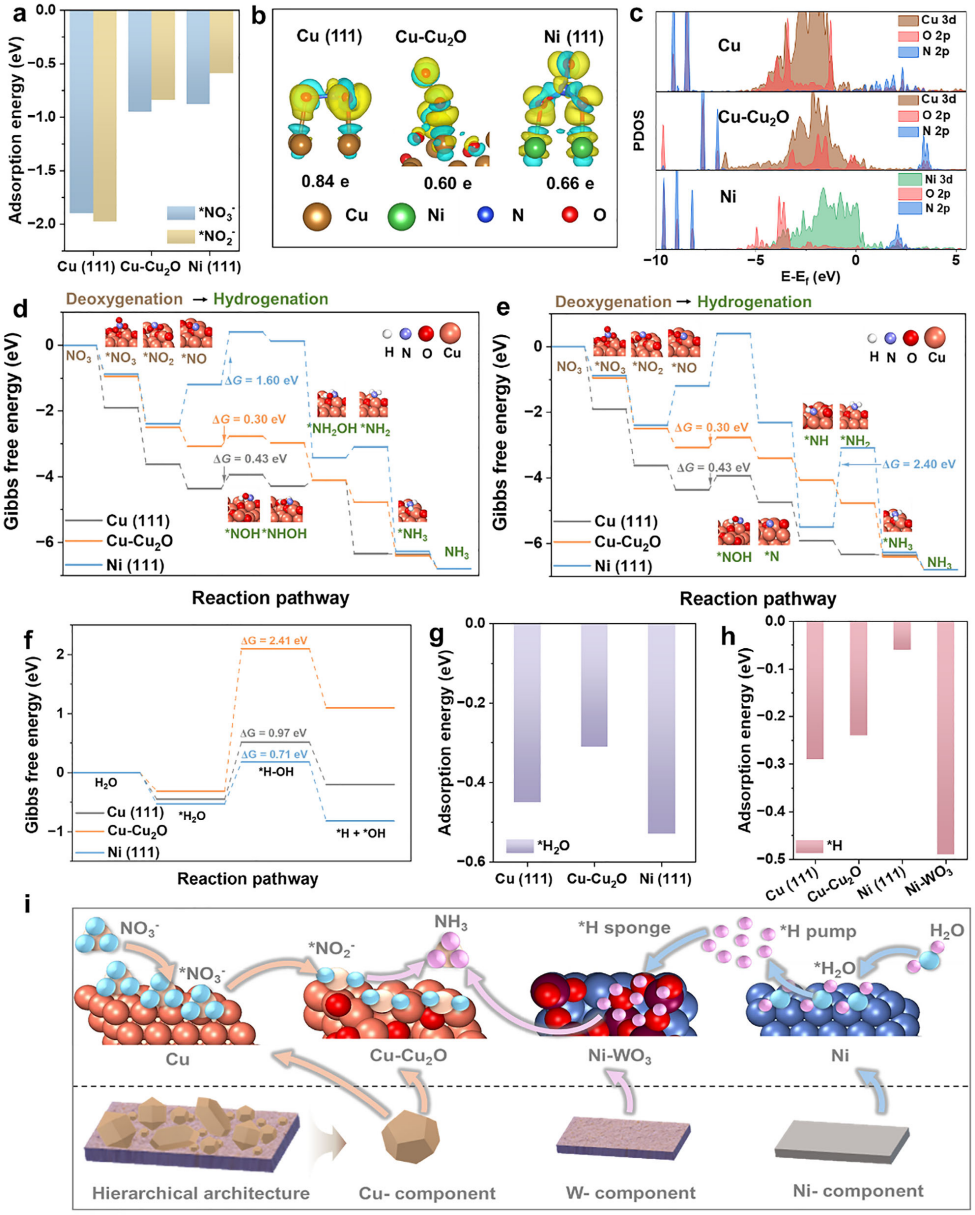
DFT calculations on the intrinsic electrocatalytic mechanism. (a) Adsorption energy of NO_3_^−^ and NO_2_^−^ intermediates on Cu (111), Cu‐Cu_2_O, and Ni (111) surfaces. (b) Charge density difference of Cu (111), Cu‐Cu_2_O, and Ni (111) surfaces after adsorption of NO_3_^−^, yellow and blue regions represent charge accumulation and depletion. (c) Projected density of states (PDOS) of NO_3_^−^ adsorbed on different surfaces: 3d orbitals of Cu and Ni, and 2p orbitals of O and N. (d) The Gibbs free energy diagrams of the NO_3_^−^RR on Cu (111), Cu‐Cu_2_O, and Ni (111) surfaces for Pathway I. (e) The Gibbs free energy diagrams of the NO_3_^−^RR on Cu (111), Cu‐Cu_2_O, and Ni (111) surfaces for Pathway II. (f) The Gibbs free energy diagrams of H_2_O dissociation on Cu (111), Cu‐Cu_2_O, and Ni (111) surfaces. (g) Adsorption energy of *H_2_O on Cu (111), Cu‐Cu_2_O, and Ni (111) surfaces. (h) Adsorption energy of *H on Cu (111), Cu‐Cu_2_O, Ni (111), and Ni‐WO_3_ surfaces. (i) Proposed tri‐functional catalytic mechanism for tandem deoxygenation and hydrogenation processes on the hierarchical architecture.

First, the adsorption configurations of NO_3_^−^ were determined. As shown in Figure 5a, NO_3_^−^ adsorbs more strongly on Cu (111) surface (−1.95 eV) than both the Cu‐Cu_2_O surface (−0.95 eV) and the Ni (111) surface (−0.88 eV). The adsorption geometries (insets in Figure 5d,e) reveal that three Cu─O bonds are formed when *NO_3_^−^ lies parallel to the Cu (111) surface, whereas only two Cu─O bonds are formed on Cu‐Cu_2_O and Ni (111) surfaces. Moreover, the charge density difference analysis (Figure 5b) shows more obvious and local electron accumulation around Cu atom on the Cu (111) surface, both indicating preferential *NO_3_^−^ adsorption on Cu sites, thereby generating NO_3_^−^‐rich microenvironment (Figure 5i). In line with this, the Bader analysis also indicates the largest charge transfer between *NO_3_^−^ and Cu (111) surface (0.84 e) as compared to Cu‐Cu_2_O (0.60 e) and Ni (111) surface (0.66 e). Projected density of states (PDOS) analysis (Figure 5c) further confirms strong Cu─O bonding on Cu (111) surface. Concretely, significant overlap between Cu 3d and O 2p orbitals occurs at −2.5 eV (bonding state) and 3 eV (anti‐bonding state), characteristic of strong covalent interaction on Cu (111) surface. For Ni (111) surface, minimal Ni 3d and O 2p overlap is observed. For the Cu‐Cu_2_O surface, PDOS demonstrates moderate Cu─O bonding, indicative of repulsive interactions from surface oxygen atoms and thereby unfavorable *NO_3_^−^ adsorption. This is further supported by the adsorption configuration of *NO_2_ (Figure 5a). As shown, Cu exhibits the strongest adsorption for *NO_2_, whereas Ni demonstrates the weakest adsorption. In contrast, Cu‐Cu_2_O displays moderate adsorption strength, indicating a favorable balance between adsorption and desorption, which effectively prevents the aforementioned undesired intermediates (e.g., *NO_2_) accumulation [23, 24].

Next, guided by online DEMS data (Figure 4), we simulated the adsorption configurations of experimentally observed reaction intermediates and the Gibbs free energy profiles of NO_3_^−^RR via two possible pathways on Cu (111), Cu‐Cu_2_O, and Ni (111) model surfaces, respectively. As illustrated, for both Pathway I (Figure 5d) and Pathway II (Figure 5e), the deoxygenation process begins with the adsorption of NO_3_^−^ reactant to form *NO_3_ intermediate, followed by stepwise N─O bond cleavage, yielding *NO_2_ and then *NO intermediates. In the hydrogenation process on Cu (111) and Cu‐Cu_2_O surfaces, for both Pathway I and Pathway II, the initial step of converting *NO to *NOH is widely recognized as a pivotal factor and the rate‐determining step (RDS) that overcame the highest energy barrier [79, 80], which coincides well with the experimental results (Figure 4). Notably, the energy barrier for the *NO to *NOH conversion is significantly lower on the Cu‐Cu_2_O surface (0.30 eV) compared to that on Cu (111) surface (0.43 eV), indicating that the Cu‐Cu_2_O surface is more energy‐efficient for NO_3_^−^RR‐to‐NH_3_. Specifically, the Pathway II (Figure 5e) is recognized as a preferential route reflected by a more homogeneous Gibbs free energy drop during *NOH → *N → *NH → *NH_2_ steps, compared to *NOH → *NHOH → *NH_2_OH → *NH_2_ steps. This is also verified by experimentally observed higher concentrations of *N and *NH in comparison to those of *NHOH and *NH_2_OH (Figure 4). Notably, different RDSs were observed for Ni (111) surface, namely *NO to *NOH (1.6 eV) for Pathway I (Figure 5d), while *NH to *NH_2_ (2.4 eV) for Pathway II (Figure 5e).

To gain mechanistic insights into the synergistic roles of participating components in promoting H_2_O dissociation, *H formation, and utilization, we performed DFT calculations along the HER pathway (Figure 5f). The computed energy profile exhibited strong dependence on the catalyst component. Notably, Ni exhibited both the strongest adsorption for H_2_O (Figure 5g) and the lowest energy barrier for its dissociation (0.71 eV), in comparison to Cu (0.97 eV) and Cu‐Cu_2_O surface (2.41 eV). However, *H adsorption on Ni is relatively weak (Figure 5h), serving as *H pump and leading to a rapid turnover that favors HER and limits *H availability for downstream hydrogenation steps in NH_3_ production. By contrast, the Ni‐WO_3_ component exhibits robust *H adsorption capability, enabling its unique role as *H sponge to create a dynamic, *H‐dense microenvironment (Figure 5i) [45, 47].

This complementary adsorption behavior underpins a tri‐functional catalytic mechanism for tandem deoxygenation and hydrogenation processes within the hierarchical architecture (Figure 5i). Specifically, Cu (111) surface creates a *NO_3_^−^‐concentrated local environment through strong adsorption; the Cu‐Cu_2_O domains primarily facilitate the reduction of *NO_3_^−^ to *NO_2_^−^ and enable balanced subsequent desorption; the Ni component thermodynamically promotes H_2_O adsorption and dissociation to generate reactive *H species; while Ni‐WO_3_ functions as both a reservoir and regulator for *H, ensuring its selective utilization toward NO_3_^−^RR‐to‐NH_3_. In this hierarchically structured composite electrode, the spatial and functional differentiation of active sites effectively suppresses the competing HER and enhances the overall efficiency of NO_3_^−^‐to‐NH_3_ conversion.

### 2.5 Adaptive Electrocatalyst Systems for Sustainable Coupled Electrolysis

**FIGURE 6:**
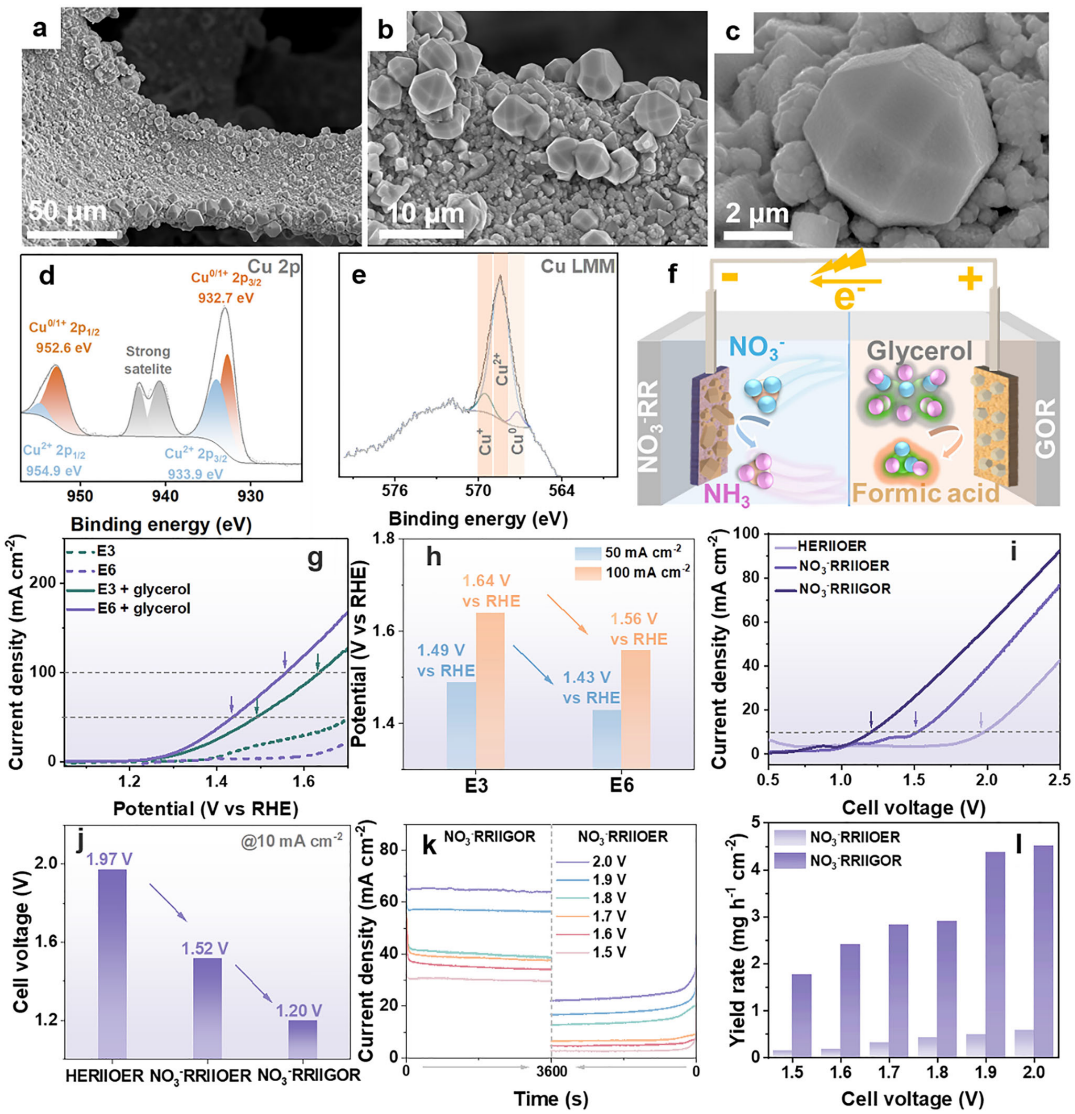
Characterization and electrochemical performance evaluation of E6. (a–c) SEM images of **E6** bulk electrode. Deconvoluted XPS spectra for (d) Cu 2p and (e) Cu LMM of **E6**. (f) Illustration of NO_3_^−^RRIIGOR configuration. (g) LSV curves of **E3** and **E6** in 1 M NaOH with (solid line) and without (dashed line) 0.1 M glycerol, scan rate: 5 mV s^−1^, without *iR* correction. (h) Comparison of potential at 50 and 100 mA cm^−2^ for **E3** and **E6** for GOR. (i) LSV curves of HERIIOER, NO_3_^−^RRIIOER, and NO_3_^−^RRIIGOR configurations and (j) corresponding cell voltages at 10 mA cm^−2^. (k) CA curves comparing NO_3_^−^RRIIOER and NO_3_^−^RRIIGOR over a range of cell voltages (1.5–2.0 V). (l) Comparison of NH_3_ yield rate for NO_3_^−^RRIIOER and NO_3_^−^RRIIGOR configurations.

The adaptation of **E3** was achieved by a facile annealing treatment, resulting in Electrode 6 (**E6**) with considerable structural and compositional transformations. SEM data (Figures 6a–c and S57) revealed the formation of abundant polyhedrons with reduced diameters (diameter: 3–5 µm), in clear contrast to the morphological characteristics observed in **E3** (Figure 1a–c and S4). These polyhedrons are uniformly distributed on the underlying base layer. SEM‐EDX elemental mapping further corroborates the retention of all constituent elements within the catalyst, with a notable surface enrichment of Cu and O across the entire electrode (Figure S58). Quantitative analysis indicates an atomic ratio of O:Cu:Ni:W of 53.1:65.6:5.3:1.0 within the catalyst layer, consistent with a CuO*_x_*‐dominated composition. Moreover, the Cu 2p and Cu LMM XPS spectra (Figure 6d,e) demonstrate a higher relative abundance of CuO in **E6** compared to **E3** (Figure 2a,b), while also confirming the coexistence of Cu_2_O and Cu^0^ species, together with Ni^2+^ and W^6+^ components (Figure S59).

Meanwhile, XRD analysis (Figure S60) supports these observations, showing distinct reflections corresponding to crystalline CuO and Cu_2_O, as well as Cu^0^ and Ni^0^ phases. Additionally, ATR‐FTIR data (Figure S61) confirm the presence of O‐H vibrations, further supporting the identification of hydroxide or hydration species on the surface. TGA analysis (Figure S62) reveals approximately 1.9 wt% water of hydration, indicating a higher content of hydrated phases compared to **E3** prior to annealing (Figure S13). The atomic ratio of Cu:Ni:W in the pure catalyst peeled off from **E6** (by prolonged sonication in ethanol) was determined to be 4.2:17.2:1.0 (Table S9) by ICP‐OES. The decreased Cu ratio compared to **E3** (Table S2) likely arises from partial surface oxidation (partial conversion of Cu_2_O and Cu^0^ to CuO) and reconstruction of Cu species. Together, these analyses confirm the facile adaptation of **E3** through a one‐step annealing process, resulting in a CuO‐rich composition that is proposed to be well‐suited for the subsequent coupled oxidative reaction.

In this study, the GOR was selected as the coupled counter‐reaction due to the thermodynamic feasibility over sluggish OER, the abundance of glycerol as a low‐value byproduct of biodiesel and bioethanol production, and its potential to be valorized into high‐value industrial products (Figure 6f), such as formic acid [81, 82, 83, 84]. Specifically, the GOR activity of **E3** and **E6** was evaluated using LSV in 1 M NaOH containing 0.1 M glycerol solution at a scan rate of 5 mV s^−1^. In the absence of glycerol, **E6** exhibited suppressed OER activity compared to **E3**, which is likely due to its CuO‐rich composition, as CuO is reported to be OER inactive [85]. Upon the introduction of glycerol, both **E3** and **E6** displayed enhanced anodic currents relative to OER, with **E6** demonstrating a considerably lower overpotential than **E3** (Figure 6g). Specifically, the potentials required to reach current densities of 50 and 100 mA cm^−2^ for **E6** were 1.43 V versus RHE and 1.56 V versus RHE, respectively, compared to 1.49 V versus RHE and 1.64 V versus RHE for **E3** (Figure 6h). These results highlight the superior GOR activity of **E6** and validate the strategic adaptation from **E3** to **E6** for anodic reaction optimization. The enhanced GOR performance is attributed to its high CuO content, which has been reported to facilitate the oxidative removal of strongly adsorbed reaction intermediates via dissociative water adsorption, thereby promoting efficient and deep glycerol oxidation toward particularly formic acid [86, 87].

Subsequently, we evaluated the performance of **E6** toward NO_3_^−^RR to ensure the rational selection of compatible cathodic and anodic electrodes for an efficient coupled NO_3_^−^RRIIGOR system. As shown (Figure S63), **E6** delivers an NH_3_ FE of 88.1%, which is inferior to that of **E3** (97.1%) under the identical pulsed electrolysis condition. This performance difference is consistent with the pronounced surface reconstruction of **E6** and the reduced availability of Cu^0^/Cu^+^ active sites, which are widely recognized as crucial for efficient NO_3_^−^RR.

Building on the outstanding individual performance observed, a coupled NO_3_^−^RRIIGOR system was elaborately developed using **E3**II**E6** two‐electrode configuration (Figure 6f), employing 1 M NaOH with 0.1 M NaNO_3_ as a cathodic electrolyte and 1 M NaOH with 0.1 M glycerol as an anodic electrolyte. As illustrated, the NO_3_^−^RRIIGOR system delivers superior overall performance compared to the NO_3_^−^RRIIOER and HERIIOER control systems (Figure 6i). At a current density of 10 mA cm^−2^, the cell voltage of the NO_3_^−^RRIIGOR configuration was only 1.20 V, which is 320 mV and 770 mV lower than those of NO_3_^−^RRIIOER and HERIIOER systems (Figure 6j). The substantial decrease in cell voltage of the **E3**II**E6** configuration signifies lower energy consumption and enhanced energy efficiency, highlighting the advancement in the adaptive electrode design strategy in advancing NO_3_^−^RRIIGOR systems.

Further, the influence of anodic oxidation on NH_3_ production was studied by analyzing the electrolyte after electrolysis in both the NO_3_^−^RRIIOER and the NO_3_^−^RRIIGOR configurations over a suite of cell potentials (1.5–2.0 V). Consistent with the LSV data (Figure 6i), the NO_3_^−^RRIIGOR configuration exhibits a significantly higher current density compared to the NO_3_^−^RRIIOER counterpart (Figure 6k). At a cell voltage of 1.9 V, vigorous O_2_ bubble formation is observed on **E6** in the NO_3_^−^RRIIOER setup, while no such evolution occurs in the NO_3_^−^RRIIGOR configuration (Figure S64). Notably, the yield rate of NH_3_ is markedly enhanced in the NO_3_^−^RRIIGOR configuration as compared to the NO_3_^−^RRIIOER counterpart (Figure 6l). Moreover, post‐electrolysis analysis of the anodic electrolyte under GOR conditions revealed the simultaneous formation of additional value‐added products [88] (Figure S65), with formic acid consistently detected as the predominant product across all tested cell potentials (Figure S66). Notably, post‐catalytic XPS analysis of **E6** (Figure S67) revealed an increased proportion of Cu^2+^ species relative to Cu^0/1+^, accompanied by a decreased surface Ni content compared with the pristine **E6** (Figure S59). These observations further support the pivotal role of the CuO component [86, 87], as discussed above, and indicate dynamic surface compositional restructuring of **E6** during the GOR process.

Furthermore, the GOR stability of the **E6** electrode was evaluated over five consecutive cycles (Figure S68). While reproducible performance is observed during the initial cycles, a gradual decrease in activity becomes apparent from the third cycle onward, indicating insufficient stability of **E6** under repeated GOR operating conditions. The observed performance decay may be related to subtle structural/surface modifications induced during long‐term operation. Importantly, these results suggest that further optimization of the substrate effects (e.g., electrode—support interactions) [89] may be required to improve the long‐term stability of **E6**.

## 3 Conclusion

In summary, we have engineered hierarchically integrated Cu‐Cu_2_O/Ni‐NiO/WO_3_ nanostructures on Ni foam via a sequential microwave‐assisted and hydrothermal deposition strategy, resulting in an adaptive tri‐component electrocatalyst for efficient electrochemical nitrate‐to‐ammonia conversion. Under pulsed electrolysis, the as‐developed Cu‐Cu_2_O/Ni‐NiO/WO_3_@NF delivers outstanding performance, achieving a remarkable FE of 97.1%, a record‐high ammonia yield rate of 43.9 mg h^−1^ cm^−2^, and competitive cycling stability over more than 14 consecutive cycles. Mechanistic insights from online DEMS reveal that pulsed electrolysis dynamically modulates key N‐intermediates, thereby steering the reaction pathway toward selective ammonia formation. Complementary DFT calculations further elucidate the synergistic roles of the Cu, Ni, and W components in establishing a self‐regulating catalytic microenvironment. Moreover, the strategic coupling of cathodic nitrate reduction with anodic glycerol oxidation to formic acid highlights the potential of this system for energy‐efficient co‐electrolysis of value‐added chemicals. Collectively, this work provides guiding principles for the design of adaptive tandem catalysts and integrated electrochemical systems toward sustainable ammonia synthesis and biomass electro‐valorization.

## Conflicts of Interest

The authors declare no conflicts of interest.

## Supporting information




**Supporting File 1**: The authors have cited additional references within the Supporting Information [1–47].

## Data Availability

The data that support the findings of this study are openly available at Zenodo.org at https://doi.org/10.5281/zenodo.17072596.
