# Supplementary material for: Sustainable Ammonia Electrosynthesis Coupled With Glycerol Valorization via an Adaptive Tri‐Component Catalyst
Source: Angew Chem Int Ed Engl. 2026 Feb 1;65(11):e22014. doi: 10.1002/anie.202522014 (PMC12970514; doi:10.1002/anie.202522014)
Supplement: Supplementary file 1 — Supporting File 1: The authors have cited additional references within the Supporting Information [1–47]. [file ANIE-65-e22014-s001.docx]

**Supporting Information**

**Sustainable Ammonia Electrosynthesis Coupled with Glycerol Valorization via an Adaptive Tri-component Catalyst**

Christean Nickel^+^,^[a]^ David Leander Troglauer^+^,^[a]^ Chia-Yu Chang,^[b]^ Tiansheng Bai,^[c]^ Tobias Rios-Studer,^[a]^ Ingo Lieberwirth,^[d]^ Kevin Sowa,^[a]^ Boris Mashtakov,^[a]^ Bahareh Feizi Mohazzab,^[a]^ Lijie Ci,^[c]^ Deping Li,^[c,^ *^]^ Xiaohang Lin,^[e,^ *^]^ Bing Joe Hwang,^[b,^ *^]^ Rongji Liu,^[a,^ *^]^ and Dandan Gao ^[a,^ *^]^

[a] M.Sc. C. Nickel, M.Sc. D. L. Troglauer, M.Sc. T. Tobias Rios-Studer, M.Sc. K. Sowa, M.Sc. B. Mashtakov, Dr. B. Feizi Mohazzab, Dr. R. Liu, Dr. D. Gao

Department of Chemistry, Johannes Gutenberg University Mainz

Duesbergweg 10-14, Mainz 55128, Germany

E-mails: rongji.liu@uni-mainz.de, dandan.gao@uni-mainz.de

[b] M.Sc. C. Chang, Prof. Dr. B. Hwang

Sustainable Electrochemical Energy Development (SEED) Center, National Taiwan University of Science and Technology

Taipei, 106335 Taiwan

E-mail: [bjh@mail.ntust.edu.tw](mailto:bjh@mail.ntust.edu.tw)

[c] M. Sc. T. Bai, Prof. Dr. L. Ci, Prof. Dr. D. Li

State Key Laboratory of Advanced Welding and Joining, School of Materials Science and Engineering

Harbin Institute of Technology (Shenzhen), Shenzhen 518055, People's Republic of China

E-mail: lideping@hit.edu.cn

[d] Dr. I. Lieberwirth,

Department of Physical Chemistry of Polymers, Max Planck Institute for Polymer Research

Ackermannweg 10, Mainz, 55128 Germany

[e] Prof. Dr. X. Lin

Key Laboratory for Liquid-Solid Structural Evolution and Processing of Materials, Ministry of Education, School of Materials Science and Engineering

Shandong University, Jinan 250061, People's Republic of China

E-mail: lxh12345@sdu.edu.cn

+ these authors contributed equally to the manuscript

**Table of Contents**

**1. Instrumentation and experimental methods**

**2. Theoretical calculation methods**

**3. Synthetic section**

**4. Analytical section of electrode for nitrate-to-ammonia**

**5. Electrochemical studies**

**6. Theoretical studies**

**7. Analytical section of adaptive electrode for glycerol valorization**

**8. References**

**9. Author contributions**

**1. Instrumentation and experimental methods**

**Powder X-ray diffraction (XRD)** patterns were recorded on a STADI-P diffractometer (STOE&Cie) equipped with Mo-Kα1 radiation (λ = 0.70930 Å), a Ge (111) monochromator, and a MYTHEN 1K detector (Dectris).

**Scanning electron microscopy (SEM)** images and **energy dispersive X-ray spectroscopy (EDX)** analytical data were obtained using a Hitachi 5200 SEM equipped with an EDX detector.

**Transmission electron microscopy (TEM)** was carried out using a FEI Tecnai F30 S-TWIN at 300 kV equipped with a Gatan US4000 CCD-camera (4096 x 4096 pixels). A 150 µm condenser aperture and standard illumination settings were used for TEM image acquisition. Scanning TEM (STEM) and local EDX measurements were performed with a Philips CM 20 TEM (200 kV acceleration voltage) equipped with an EDAX Si-Li EDX detector. Prior to the TEM investigations, the samples were dispersed in ethanol using an ultrasonic bath and sprayed on a carbon-coated Au grid.

**Attenuated total reflection Fourier transform infrared spectroscopy (ATR-FTIR)** was carried out on a Bruker Alpha II equipped with a PIKE Miracle Diamond ATR unit.

**Thermogravimetric analysis** **(TGA)** was performed by METTLER TOLEDO®. Analysis of the sample was carried out on a TGA 2 STARe system under N_2_ flow with a flow rate of 60 mL min^-1^. A crucible made of polycrystalline aluminium oxide (PCA/Saphir) was used in a temperature range of 25 - 1000 °C. A heating rate of 10 °C min^-1^ was applied.

**X-ray photoelectron spectroscopy (XPS)** measurements were performed with monochromatized Al Kα exciting X-radiation using a PHI Quantera SXM system. The binding energies were calibrated based on C 1s (284.8 eV)

**Inductively coupled plasma optical emission spectrometry (ICP-OES)** was performed on a Perkin Elmer Plasma 400 spectrometer. All measurements were conducted in aqueous HNO_3_ solution.

**Ultraviolet-visible-near infrared (UV-Vis-NIR) absorbance** measurements were performed using a Cary 3500 UV/Vis-NIR Spectrophotometer equipped with a Xenon flash lamp (250 Hz). Measurements were performed in standard 1.0 cm cuvettes.

**Ex-situ X-ray absorption near edge spectroscopy (XANES) measurements** at the Cu K-edge, Ni K-edge and W L_3_-edge were conducted in fluorescence mode using Lytle detectors at beamline TPS 44A of the National Synchrotron Radiation Research Center (NSRRC), Taiwan. **Extended X-ray absorption fine structure (EXAFS)** analysis was performed by Fourier transforming the k^3^-weighted χ(k) oscillations, allowing evaluation of the local bonding environments. Data reduction and fitting were carried out using the Athena and Artemis software packages within the IFEFFIT suite.

**Online differential electrochemical mass spectrometry (DEMS)** tests were carried out on a differential electrochemical mass spectrometer (QAS100). In the DEMS configuration, the detection inlet is positioned directly beneath the cathode, whereas the anode is separated by a defined distance (ca. 8 mm), greatly suppressing the contribution from anodically evolved O_2_. As-prepared electrodes, platinum wire and mercury/mercury oxide electrodes were used as the working electrode, counter electrode and reference electrode for the electrochemical cell, respectively. The electrolyte consists of 1 M NaOH and 0.1 M NaNO_3_, which was purified with high-purity argon for at least 30 min before use. A 100 s chronocurrent determination was performed to detect the corresponding quality signal for static electrolysis (at - 0.6 V vs RHE) and pulsed electrolysis mode (at -0.5 V vs RHE for 1 s, -0.7 V vs RHE for 2 s). After the electrochemical test concluded and the mass signal returned to baseline, the following cycles were performed under the same conditions to minimize accidental errors during the DEMS measurements.

**Ammonia quantification using UV-Vis spectroscopy**

Ammonia concentration was quantified using UV-Vis spectroscopy, employing a calibration curve derived from aqueous NH_4_Cl solutions of varying concentrations. The stock solution was prepared by initially dissolving 0.1 g of NH_4_Cl in 100 mL of 1 M NaOH solution, which was subsequently diluted to prepare a series of standard solutions with NH_4_Cl concentrations of 0.1 µg mL^-1^, 0.2 µg mL^-1^, 0.5 µg mL^-1^, 1.0 µg mL^-1^, 2.0 µg mL^-1^, and 5.0 µg mL^-1^.

For the derivation of the calibration curve, 2 mL of each standard solution was combined with 2 mL of a reagent solution composed of 1 M NaOH containing 5 wt.% salicylic acid and 5 wt.% sodium citrate. This mixture was followed by the addition of 1 mL of 0.05 M NaClO solution and 0.2 mL of water containing 1 wt.% sodium nitroprusside as a coloring agent. The final reaction mixture was allowed to react in the dark for 120 minutes to ensure adequate color development. After the reaction period, UV-Vis spectra of the resulting solutions were recorded in the range of 500 to 800 nm at a scan rate of 0.2 nm s^-1^. The maximum absorption peak at 655 nm was utilized for the calibration line. The ammonia concentration in the unknown samples was determined by measuring their absorbance at this wavelength and by referencing the established calibration curve.

**Ammonia quantification using nuclear magnetic resonance (NMR) spectroscopy**

In addition to the UV-Vis spectroscopy method, ammonia concentration was also quantified using NMR spectroscopy, with the assistance of a calibration curve constructed from standard NH_4_Cl solutions with varying concentrations. Similar to before, the stock solution was prepared by initially dissolving 0.1 g of NH_4_Cl in 100 mL of 1 M NaOH solution, which was subsequently diluted to prepare a series of standard solutions with NH_4_Cl concentrations of 0.2 μg mL^-1^ (ppm), 1.0 μg mL^-1^ (ppm), 2.0 μg mL^-1^ (ppm), 4.0 μg mL^-1^ (ppm), and 5.0 μg mL^-1^ (ppm).

Each of these standard solutions was then treated by adding 2.5 mL of 4 M aqueous H_2_SO_4_, ensuring thorough mixing to stabilize the ammonia in the solution. From the resulting mixture, 540 μL was transferred to an NMR tube, followed by the addition of 60 μL of d6-DMSO containing 0.04 wt.% maleic acid as an internal standard. ^1^H NMR spectra were then recorded for each sample. The calibration curve was derived by analyzing the chemical shift and integration of the ammonia-related signals in the NMR spectra. The ammonia concentration in the unknown samples was subsequently determined by measuring the intensity of the relevant peaks in their NMR spectra and referring to the established calibration line.

**Nitrite quantification using UV-Vis spectroscopy**

Nitrite concentration was quantified using UV-Vis spectroscopy, employing a chromogenic agent to develop a detectable color reaction. To prepare the chromogenic agent, 0.5 g of sulfanilic acid was dissolved in 90 mL of water and 5 mL of acetic acid. Following this, 5 mg of N-(1-naphthyl)-ethylenediamine dihydrochloride was added to the solution, and the mixture was diluted to a final volume of 100 mL with water.

For the measurement, 1 mL of the electrolyte sample was combined with 4 mL of the chromogenic agent. The resulting solution was allowed to react in the dark for 15 minutes to ensure the development of the colorimetric reaction. After this incubation period, the UV-Vis absorption spectrum of the solution was recorded at a wavelength of 540 nm. Nitrite concentration was determined using external calibration with standard solutions of NaNO_2_ in 0.1 M NaOH. The calibration standards covered concentrations of 0.0 μg mL^-1^, 0.1 μg mL^-1^, 0.2 μg mL^-1^, 0.5 μg mL^-1^, 1.0 μg mL^-1^, 2.0 μg mL^-1^, and 5.0 μg mL^-1^. The absorbance at 540 nm from the samples was compared to the calibration curve to quantify the nitrite concentration in the measuring samples.

**Calculation of the NH_3_ yield rate, Faradaic efficiency (FE) and partial current density**

The yield rate of NH_3_ in µg h^-1^ cm^-2^ was calculated as follows:

$$Yield rate= \frac{c_{{NH}_{3}}\cdot V}{t\cdot A}$$

The yield rate of NO_2_^-^ was calculated similarly to the yield rate of NH_3_, with the corresponding concentration of NO_2_^-^ ($c_{{NO}_{2}^{-}}$)

The FE was calculated as follows:

$$FE=\frac{8\cdot F\cdot c_{{NH}_{3}}\cdot V}{M_{{NH}_{3}}\cdot Q} \cdot100\%$$

The FE of NO_2_^-^ was calculated similarly to the FE of NH_3_, with the corresponding concentration and molar mass of NO_2_^-^ ($c_{{NO}_{2}^{-}}$, $M_{{NO}_{2}^{-}}$)

In the equations $c_{{NH}_{3}}$ (µg mL^-1^) is the concentration of ammonia in solution, 𝑉 (mL) is the volume of the electrolyte in the cathode compartment, 𝑡 (h) is the time of the electrolysis, 𝐴 (cm^-2^) is the surface area, 𝐹 is the Faradaic constant (96485 C mol^–1^), $M_{{NH}_{3}}$ is the molar mass of NH_3_ (17.0 g mol^-1^), $M_{{NO}_{2}^{-}}$ is the molar mass of NO_2_^-^ (46.0 g mol^-1^) and 𝑄 is the total charge passing through the system during the electrolysis.

The calculation method for partial current density of NH_3_ (*j*_NH3_):

*j*_NH3_ = *FE*_NH3_ × *I*_it_

In the equation, *I*_it_ is the average current density (mA cm^−2^) at given potentials.^[1,2]^

**GOR product quantification using NMR**

Faradaic efficiency (FE) was evaluated by chronoamperometry at each applied potential in an H-cell separated by a Nafion 117 membrane. After one hour of electrolysis, electrolyte samples (600 µL) were collected and mixed with dimethyl sulfoxide (5.42 mM in D_2_O) as an internal standard. The liquid-phase products were analyzed by nuclear magnetic resonance (NMR) spectroscopy on a Bruker Avance 400 instrument. For product quantification, a one-dimensional solvent suppression sequence employing excitation sculpting with gradients (zgesgp) was applied. The faradaic efficiencies for a product *i*, FE*_i_* were calculated based on the following equation:

$$\mathrm{FE}_{i}=\frac{\alpha_{i}n_{i}F}{Q} x 100\%$$

where αᵢ represents the electron count per molecule for each product, nᵢ is the moles of product, F is the Faraday constant (96485 C/mol), and Q is the total charge.

## Chemicals

Sodium tungstate dihydrate (VWR, CAS No. 10213-10-2), Sodium metasilicate pentahydrate (VWR, CAS No. 10213-79-3), Potassium chloride (99.0%-100.5%, Carl Roth, CAS No. 7447-40-7), Copper (II) nitrate trihydrate (99%, Acros Organics, CAS No. 10031-43-3), Hydrochloric acid (37%, VWR, CAS No. 7647-01-0), Sodium hydroxide (pellets, analytical grade, Fischer scientific, CAS No.1310-73-2), Sodium nitrite (≥ 99%, Carl Roth, CAS No. 7632-00-0), Ammonium chloride (≥ 99.7%, Carl Roth, 12125-02-9), Sodium nitrate (≥ 99.0%, Sigma-Aldrich, CAS No. 7631-99-4), Sodium hypochlorite (5% active chlorine, Thermo Fisher Scientific, CAS No. 7681-52-9), Sodium pentacyanonitrosylferrate (III) dihydrate (> 98%, Alfa Aesar, CAS No. 13755-38-9), Trisodium citrate dihydrate (99%, Alfa Aesar, CAS No. 6132-04-3), Salicylic acid (VWR, CAS No. 69-72-7), Glycerol (ROTIPURAN, ≥ 99.5%, Carl Roth, CAS No. 56-81-5), Ammonium-^15^N-chloride (≥98 atom % ^15^N, ≥99% (CP), Sigma-Aldrich, CAS No. 39466-62-1), Sodium nitrate-^15^N (98 atom % ^15^N, 99% (CP), Sigma-Aldrich, CAS No. 31432-45-81). Commercial Nickel foam was purchased from Alantum Europe GmbH (NF, pore size: 450 µm, areal density: 420 g cm^-2^, dimension: 300 x 200 x 1.6 mm^3^). Nafion 117 membrane was purchased from Frontcell Energy (thickness: 175 μm, specific weight: 345 mg cm^-2^). All chemicals were used as received.

**2. Theoretical calculation methods**

In this work, all density functional theory (DFT) calculations were performed using the periodic electronic structure code VASP^[3]^ within the generalized gradient approximation (GGA) to describe the exchange-correlation effects, employing the Perdew Burke-Ernzerhof (PBE) exchange-correlation functional.^[4,5]^ The electronic one-particle wave functions were expanded in a plane-wave basis set with an energy cutoff of 520 eV. It has been carefully checked that the k points sets used for Brillouin zone integration were sufficiently large to achieve convergence. Structures were relaxed until the interaction force fell below 0.01 eV Å^-1^. Solvation effect was incorporated into the adsorption free energy of intermediates using a polarized continuum model in VASPsol^[6]^ with the solvent dielectric constant set to 80 (corresponding to water at room temperature).

The adsorption energy was determined according to:

$$E_{ads}=E_{tot}-E_{substrate}-E_{adsorbate}$$

where *E*_tot_, *E*_sub_ and *E*_atom_ are the energies of the total adsorption system, the graphene substrate and the isolated atom, respectively.

The amorphous structures of Cu-Cu_2_O and Ni-WO_3_ were obtained via the melt-quenching method based on ab initio molecular dynamics (AIMD) simulations.^[7,8]^ According to the TEM, XRD, XPS and XAS data, the initial structure of amorphous Cu-Cu_2_O was constructed by adsorbing six O_2_ molecules on a 4 × 4 Cu (111) surface to simulate Cu surface oxidation. The system was then heated and equilibrated at 1400 K to accelerate oxidation. After 15 ps, the structure remained stable. Subsequently, it was rapidly cooled to 300 K in 10 ps (cooling rate: 110 K ps^-1^). Bader analysis indicates that the copper valence state in the oxide layer is approximately + 0.96, consistent with experimental data. Amorphous Ni-WO_3_ has a composition of Ni_64_W_8_O_24_ with a Ni (111)-WO_3_ heterostructure. This heterostructure was heated to 1400 K, maintained for 15 ps, and cooled to 300 K in 10 ps. All AIMD simulations used the Nosé-Hoover thermostat. Finally, the final configurations were further optimized by DFT to release atomic forces, obtaining amorphous slabs at 0 K. Additionally, the Wulff constructions of Cu and Ni crystals were built based on surface energies previously reported in a relevant work.^[9]^

**3. Synthetic section**

**3.1 Synthesis of K_8_[α-SiW_11_O_39_]·13H_2_O**

The compound was synthesized according to the published procedure.^[10–12]^ Purity was confirmed by ATR-FTIR (characteristic bands are given as wavenumbers in cm^-1^ using the following abbreviations: s, strong; m, medium; w, weak): 3,387 (s), 161 (s), 995 (m), 954 (s), 864 (s), 788 (m), 710 (s), 503 (m), and 464 cm^-1^ (w).

**3.2 Preparation of the NF electrode**

Before deposition, the NF was cut into 10 x 30 x 1.6 mm^3^ size and sonication-washed with acetone, isopropanol, 1.0 M HCl and deionized water, respectively, to remove surface impurities (immersion time: 15 min per solvent).

**3.3 Working electrode preparation**

The composites are prepared by microwave (**MW**) assisted and / or hydrothermal (**HT**) deposition process in a precursor solution of Cu(NO_3_)_2_·3H_2_O (0.11 mmol) and K_8_[SiW_11_O_39_]·13H_2_O (1.0 mmol). For mono- **MW** deposition, the cleaned **NF** was placed inside a tube vial containing 4.5 mL of the precursor solution, which was heated by microwave (Biotage Initiator+, Absorption: VH) to 140°C for 20 min with constant stirring. After cooling to room temperature, the obtained composite electrode was collected, rinsed with deionized H_2_O and ethanol, and subsequently air-dried, leading to Electrode 1 (**E1**). For mono- **HT** deposition, the cleaned **NF** was placed in a 100 mLTeflon-lined stainless-steel autoclave containing 40 mL of the precursor solution, which was heated at 150 °C for 6 h under autogenous pressure. After cooling to room temperature, the obtained composite electrode was collected, rinsed with deionized H_2_O and ethanol, and subsequently air-dried, leading to Electrode 2 (**E2**). For adjacent **MW** and **HT** deposition, the mono-step aforementioned is combined, leading to Electrode 3 (**E3**).

To elucidate the role of the individual components in the precursor solution, the adjacent MW and HT deposition process was carried out identically but in the presence of only Cu^2+^ (resulting in Electrode 4, **E4**) or [SiW_11_O_39_]^8-^ (resulting in Electrode 5, **E5**).

To investigate the influence of the concentration of the precursor solution, two more ratios, Cu^2+^ : [SiW_11_O_39_]^8-^ = **2 : 1** and **1: 2**, were employed in the synthetic process.

**Table S1**. Components of precursor solutions for the **MW** and / or **HT** deposition.

|  |  | **E1** | **E2** | **E3** | **E4** | **E5** | **2:1** | **1:2** |
| --- | --- | --- | --- | --- | --- | --- | --- | --- |
| **Deposition method** | | **MW** | **HT** | **MW + HT** | **MW + HT** | **MW + HT** | **MW + HT** | **MW + HT** |
| **Precursor concentration**  **(mmol mL^-1^)** | Cu^2+^ | 0.026 | 0.026 | 0.026 | 0.026 | - | 0.052 | 0.026 |
|  | [SiW_11_O_39_]^8-^ | 0.027 | 0.027 | 0.027 | - | 0.027 | 0.027 | 0.054 |

**Electrochemical pre-reduction**

Electrochemical reduction of the **E3** was conducted at a constant -0.6 V vs RHE in a single electrochemical cell with a three-electrode configuration (working electrode: obtained composite electrode, reference electrode: saturated calomel electrode, counter electrode: platinum wire) in Ar-purged 0.1 M KHCO_3_ electrolyte (pH 8.3, 30 mL). The reduction process was conducted for ca. 15 min until a stable current was achieved, to convert the catalyst components into well-defined initial catalytic conditions^[13]^ (e.g., modified surface oxidation state and crystal structure) prior to nitrate reduction.

**Adaptation of E3 for glycerol oxidation reaction**

The adaptation of **E3** was achieved by a facile one-step annealing process at 300 °C for 3 h under air. After cooling to room temperature, Electrode 6 (**E6**) was used as obtained.

**3.4 Electrochemical measurements**

Electrochemical measurements were performed on an AMETEK Scientific Instruments workstation (PMC 2000A) in a two-compartment electrochemical cell separated by a proton exchange membrane (Nafion 117) with a three-electrode configuration (working electrode: as-prepared electrodes based on **NF**, reference electrode: mercury / mercury oxide electrode, counter electrode: platinum wire) in Ar-saturated electrolyte (70 mL for both compartments). 1 M aqueous NaOH containing 0.1 M NaNO_3_ (pH 13.8) and 1 M aqueous NaOH containing 0.1 M glycerol (pH 13.8) were used for NO_3_^-^RR and GOR, respectively. Before use, the Nafion 117 membrane was pretreated by slightly boiling (at 80 °C) in 5 wt.% H_2_O_2_ solution, distilled water, 0.1 M H_2_SO_4_ solution and distilled water for 1 h, respectively (treatment time: 1 h per solution). All potentials were converted to the reversible hydrogen electrode (RHE) according to the Nernst equation (E_RHE_ = E_Hg/HgO_ + E_Hg/HgO0_ + 0.059 V × pH).

## Calculation of ECSA:

The electrochemically active surface area (ECSA) was examined by the electrochemical double layer capacitance ($C_{dl})$ analysis of the catalyst, which was determined from the linear change of current density from CV curves under an incremental scanning rate from 5 to 30 mV s^‑1^, in the potential window from 0.45 to 0.51 V vs RHE.

The ECSA was then calculated based on the following equations:

| $\Delta J = 2v*C_{dl}$ |
| --- |
| $ECSA =C_{dl}/Cs$ |

Where $v$ is the scanning rate, $\Delta J$= $(J_{A}$ - $J_{C}$ ) is charging current density differences at 0.48 V vs RHE, $C_{dl}$ is double-layer capacitance and $Cs$ is specific capacitance of the catalyst (40 μF cm^-2^ per cm^2^).^[14]^

**4. Analytical section of electrode for nitrate-to-ammonia**


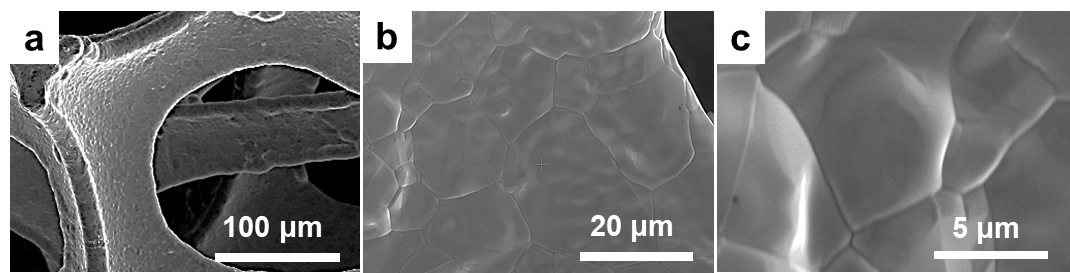


**Fig. S1**. SEM images of **NF** at different magnifications.


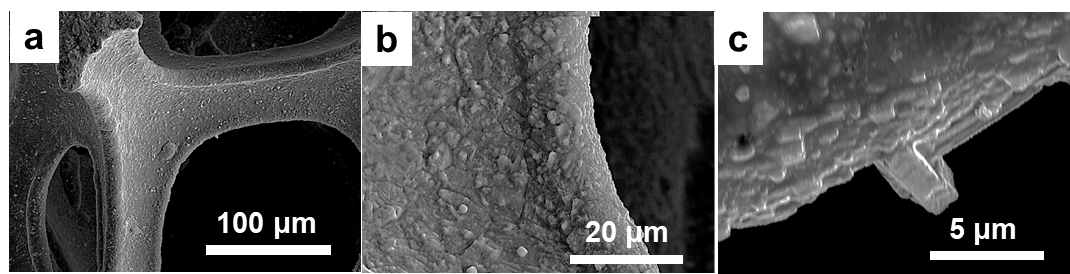


**Fig. S2**. SEM images of bulk **E1** at different magnifications.


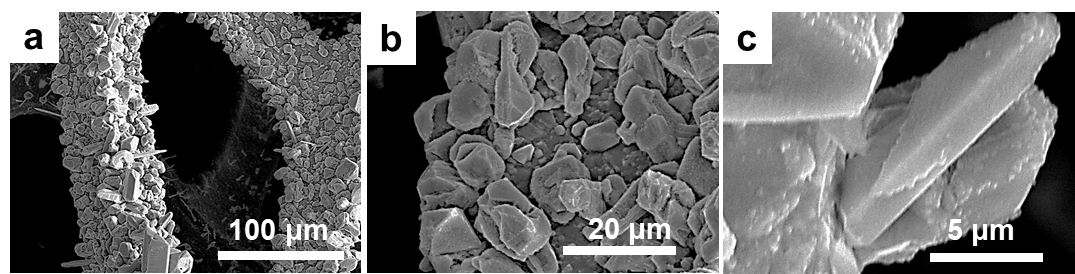


**Fig. S3.** SEM images of bulk **E2** at different magnifications.


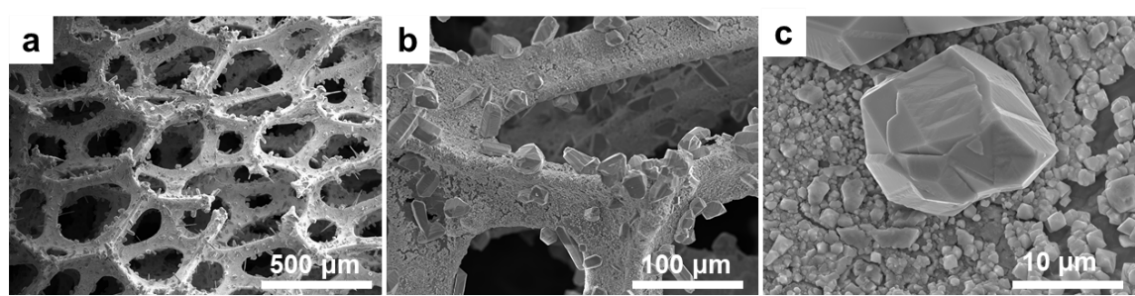


**Fig. S4.** SEM images of bulk **E3** at different magnifications.


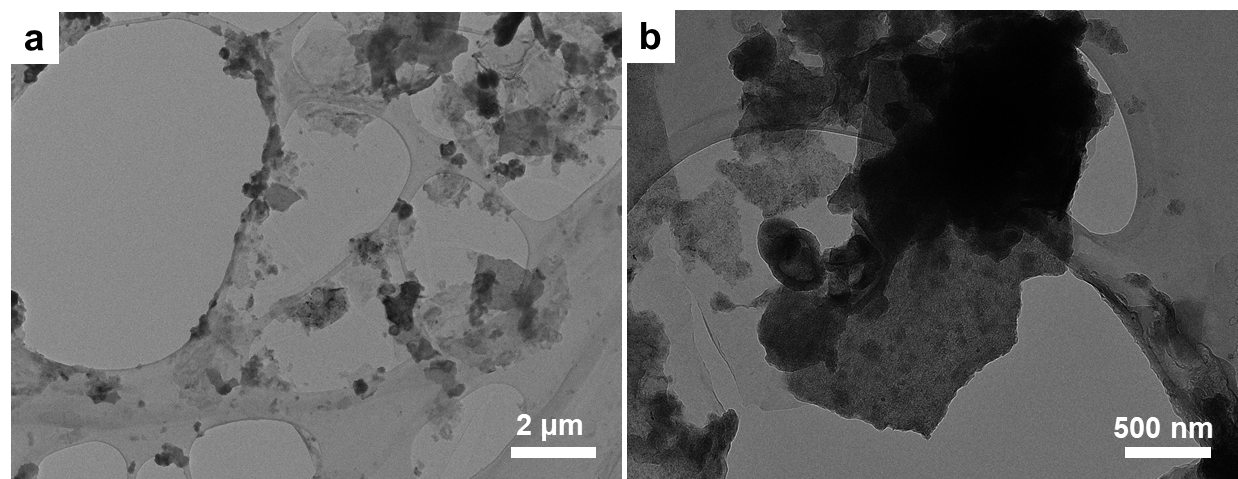


**Fig. S5**. TEM images of pure catalyst removed from **E3**.

**
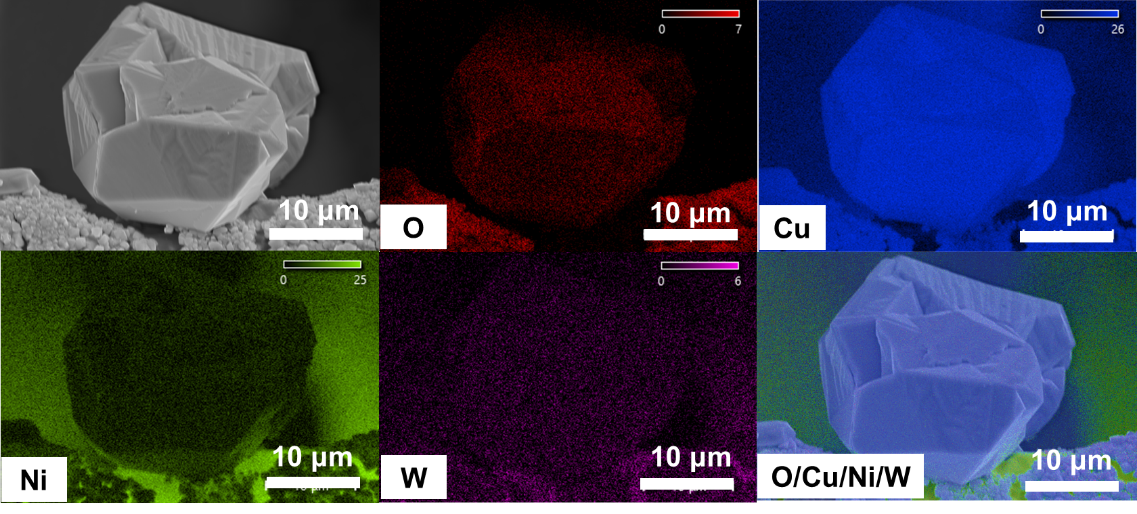

Fig. S6**. SEM-EDX elemental mapping of bulk **E3**.

**
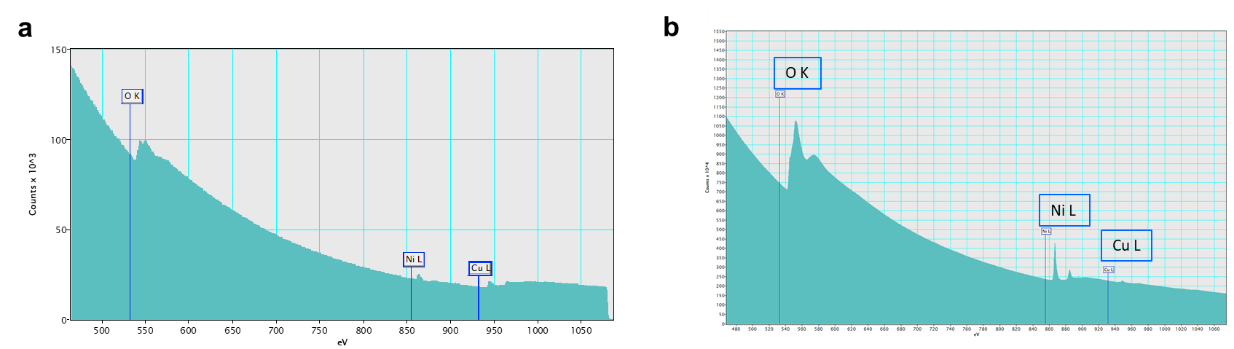
**

**Fig. S7.** EELS mapping analysis of (a) Cu-rich (**Fig. 1e**) and (b) Ni-rich particle (**Fig.** **1f**).

**
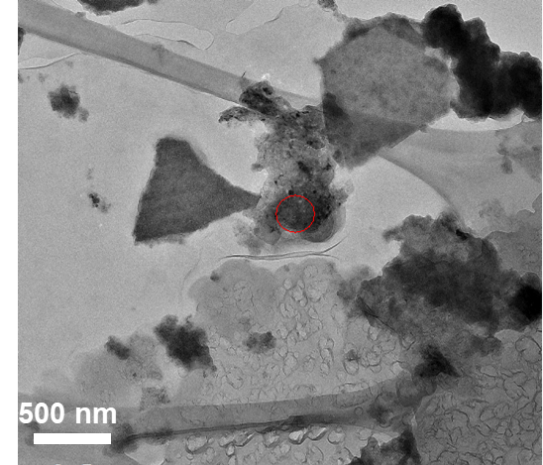
**

**Fig. S8.** Corresponding area of the SAED of Cu-rich particle in **Fig. 1g.**

**
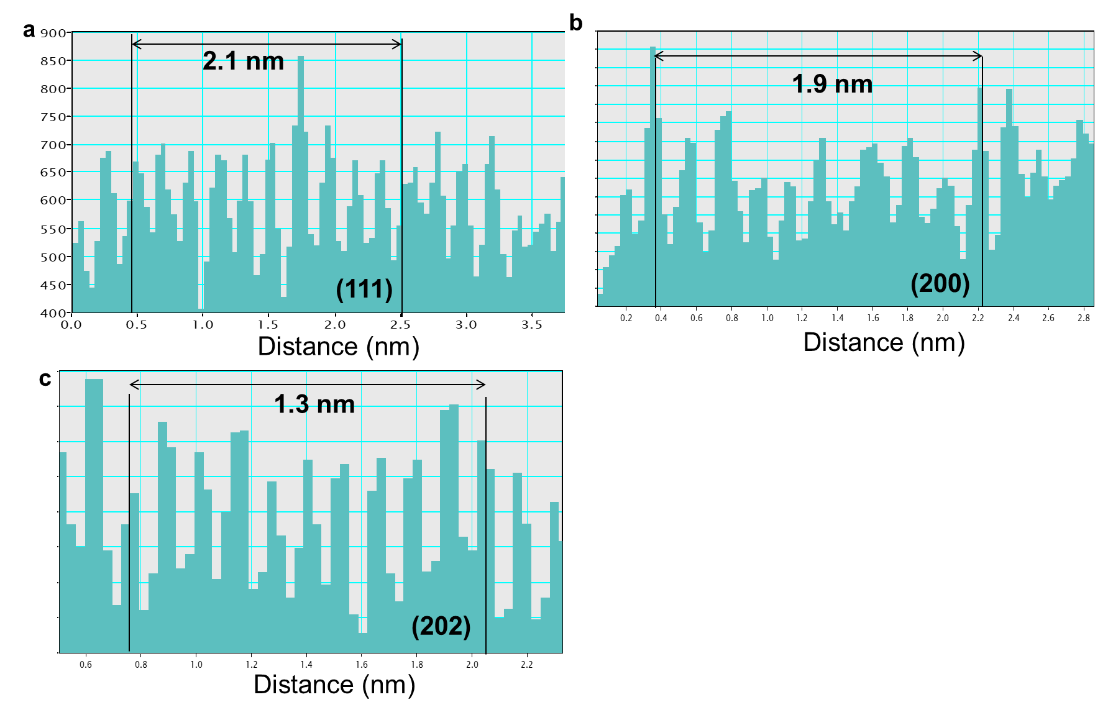
**

**Fig. S9.** Corresponding line intensity profiles of (a) (111), (b) (200) and (c) (202) planes of Cu in **Figs. 1h** and **1i.**

**
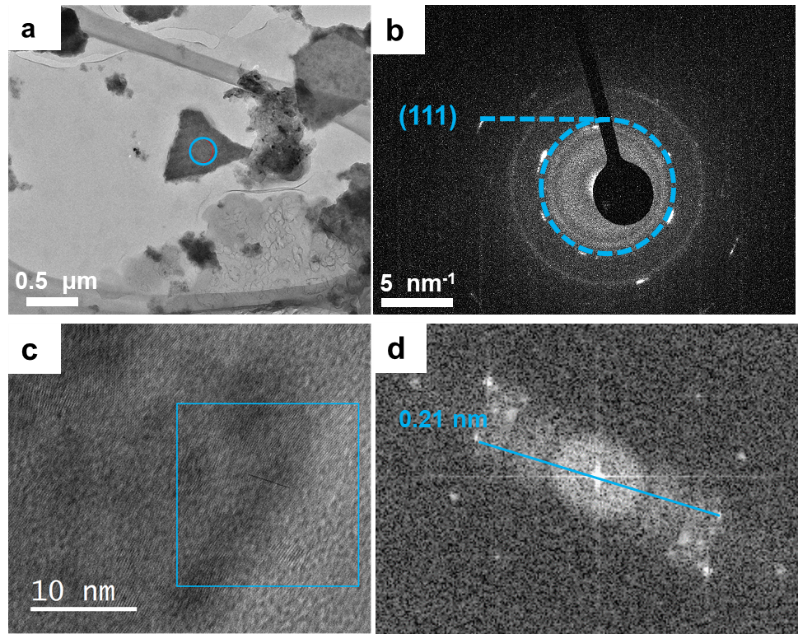
**

**Fig. S10.** (a) TEM image, (b) SAED, (c) high resolution image and (d) corresponding FFT image of Ni-rich particle shown in **Fig. 1d**.

**
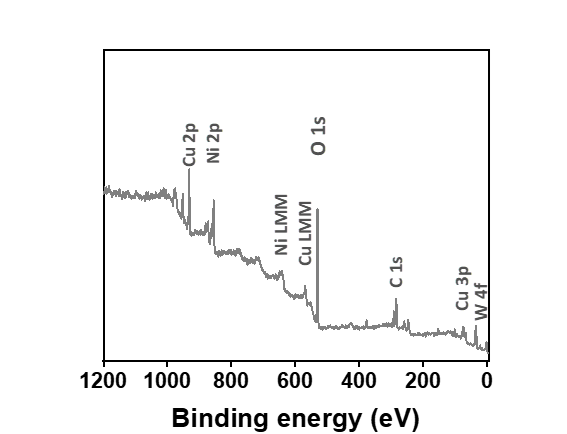
**

**Fig. S11.** XPS survey spectrum of **E3**.


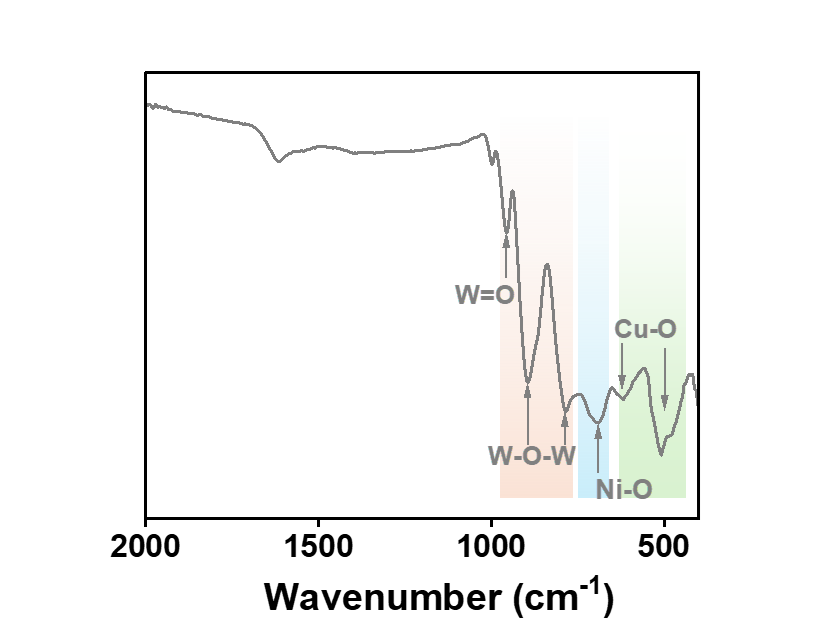


**Fig. S12.** ATR-FTIR spectra of catalyst powder removed from **E3**.

**
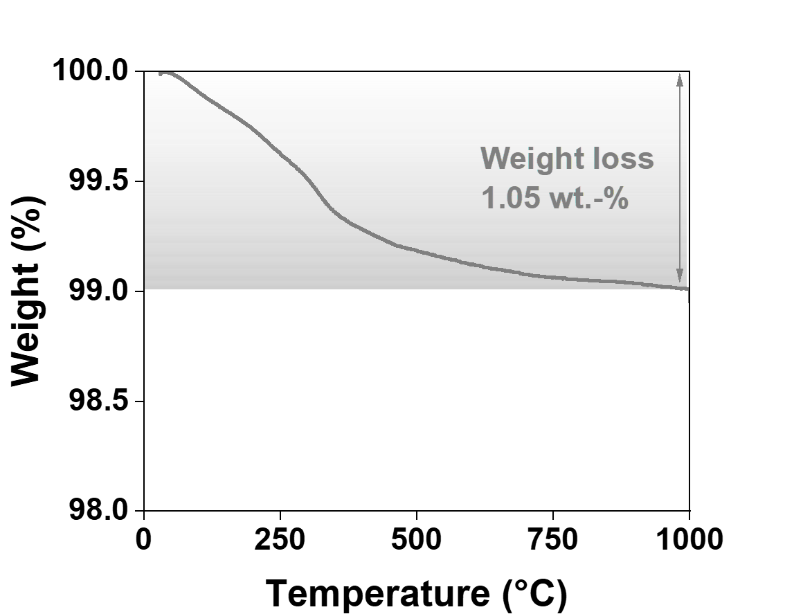
**

**Fig. S13.** Thermogravimetric analysis of catalyst powder removed from **E3**. Weight loss of 1.05 wt.% was observed between 30 to 1000 °C due to the loss of lattice water.

**Table S2.** Metal contents in the composite catalyst peeled off from **E3** based on ICP-OES analyses.

|  | **Cu** | **Ni** | **W** |
| --- | --- | --- | --- |
| **Mass ratio** | **2.48** | **2.58** | **1** |
| **Atomic ratio** | **7.16** | **8.09** | **1** |

**
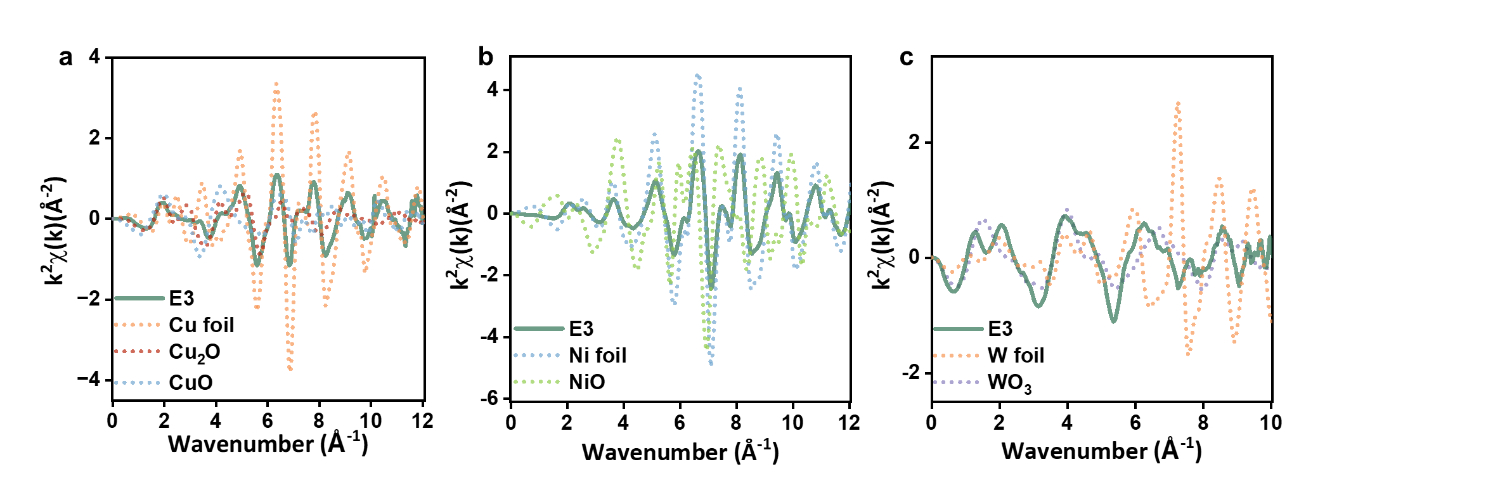
**

**Fig. S14.** k^2^-weighted EXAFS oscillations at the (a) Cu, (b) Ni, and (c) W edges.

**
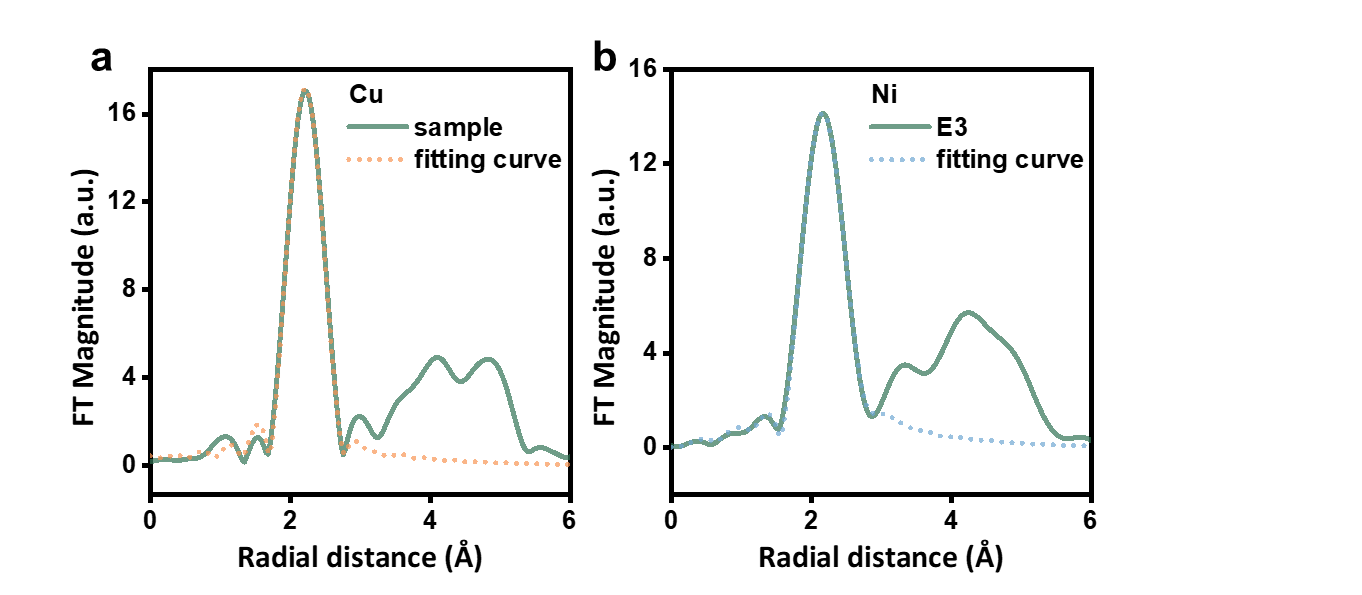
**

**Fig. S15.** EXAFS spectra of **E3** at the (a) Cu and (b) Ni K-edges with fitting curve.

**Table S3.** Cu K-edge EXAFS curve fitting parameters.

| S0^2^ : 0.9 | | | | | |
| --- | --- | --- | --- | --- | --- |
| Shell | CN | R(Å) | Debye-Waller factor (Δσ^2^) | Δ E_0_ | R-factor |
| Cu-O | 0.054 ± 0.011 | 1.89 ± 0.018 | 0.00022 ± 0.000044 | 0.251 | 1.8*10^-2^ |
| Cu-Ni | 1.8 ± 0.36 | 2.52 ± 0.025 | 0.00259 ± 0.000518 | 21.812 |  |
| Cu-Cu | 8.64 ± 1.73 | 2.58 ± 0.025 | 0.00952 ± 0.0019 | 5.246 |  |

**
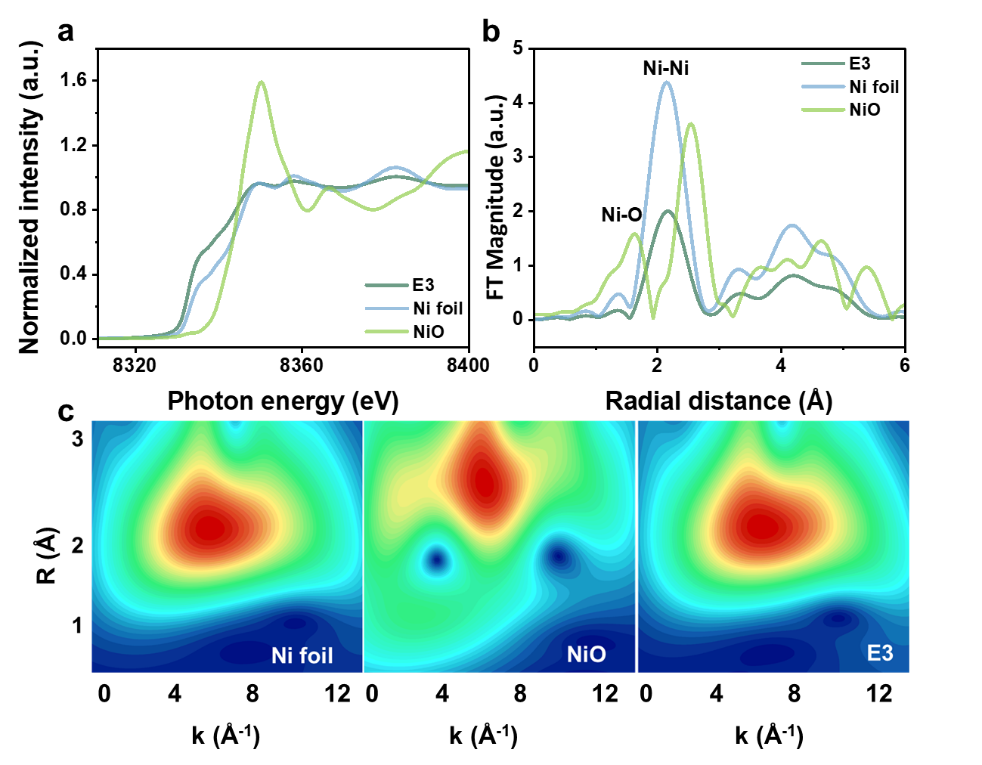
**

**Fig. S16.** Ex-situ XAS characterization of **E3**. (a) Normalized XANES spectra, (b) corresponding k^2^-weighted Fourier-transformed EXAFS spectra, and (c) WT EXAFS of the Ni K-edge for **E3**.

**
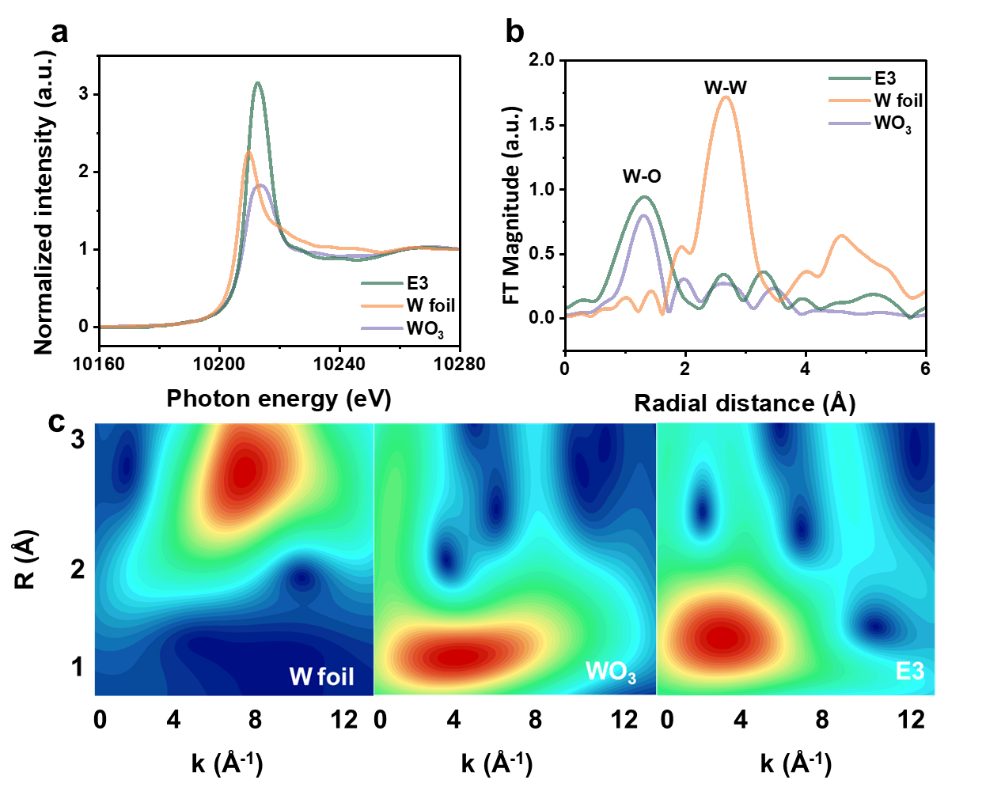
**

**Fig. S17.** Ex-situ XAS characterization of **E3**. (a) Normalized XANES spectra, (b) corresponding k^2^-weighted Fourier-transformed EXAFS spectra, and (c) WT EXAFS of the W L_3_-edge for **E3**.

**Table S4.** Ni K-edge EXAFS curve fitting parameters.

| S0^2^ : 0.85 | | | | | |
| --- | --- | --- | --- | --- | --- |
| Shell | CN | R(Å) | Debye-Waller factor (Δσ^2^) | Δ E_0_ | R-factor |
| Ni-Ni | 1.7 ± 0.34 | 2.51 ± 0.025 | 0.002 ± 0.0004 | 20.723 | 4.8*10^-3^ |
| Ni-Cu | 8.16 ± 1.632 | 2.52 ± 0.025 | 0.0153 ± 0.0030 | 2.596 |  |

**
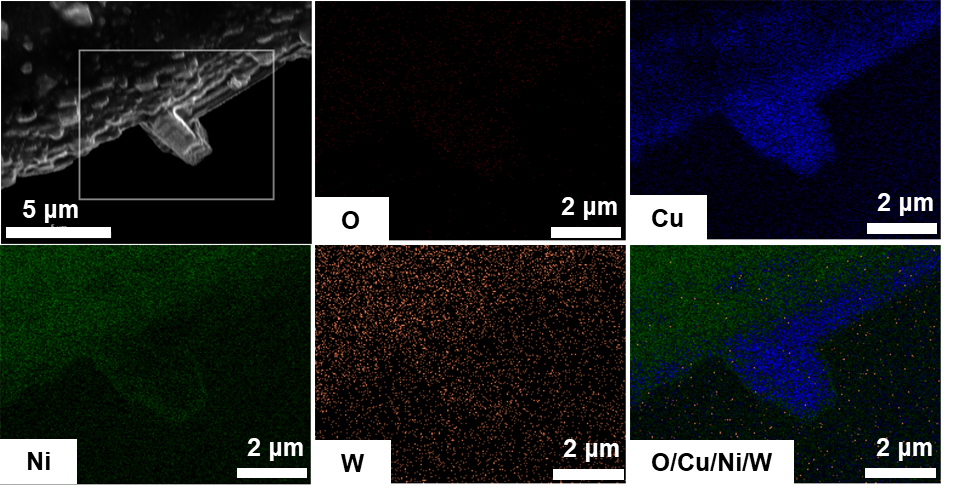
**

**Fig. S18**. SEM-EDX elemental mapping of bulk **E1**.

**
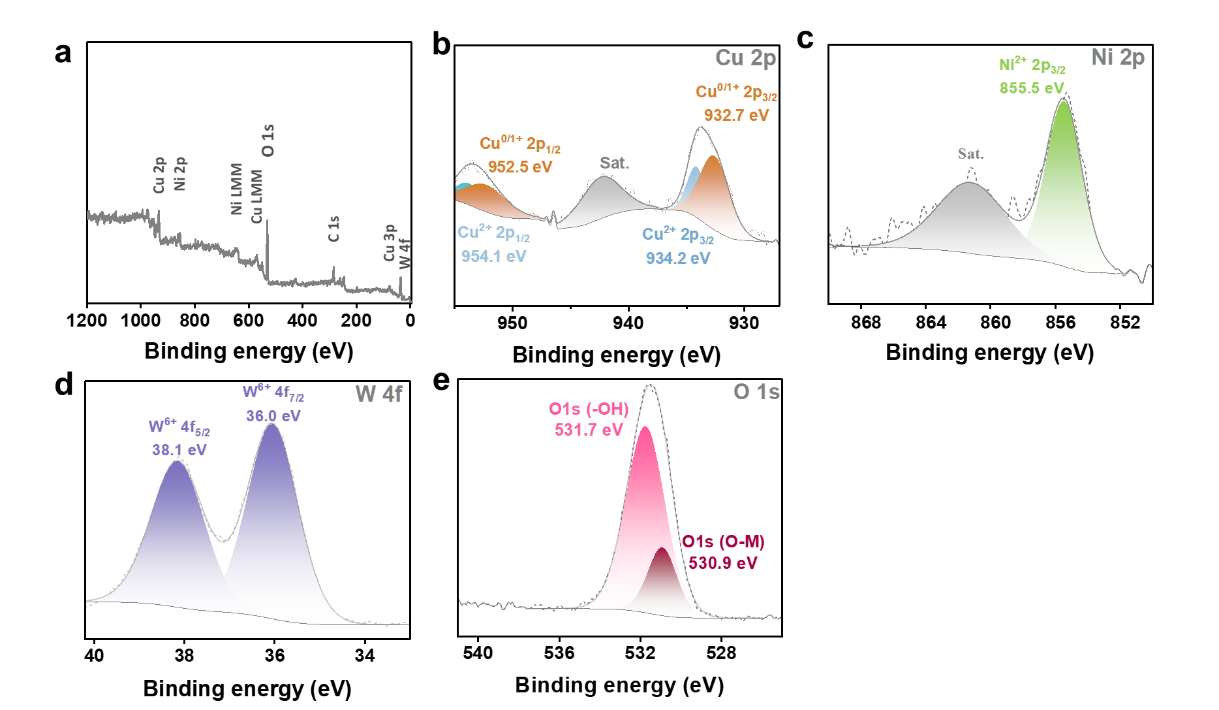
**

**Fig. S19**. (a) Survey spectrum and (b-e) high-resolution XPS spectra of bulk **E1**.

**
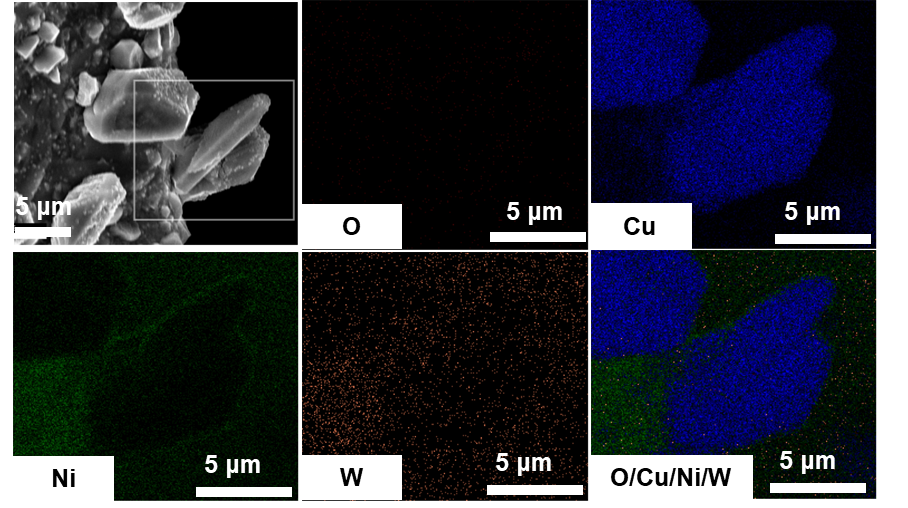
**

**Fig. S20**. SEM-EDX elemental mapping of bulk **E2**.


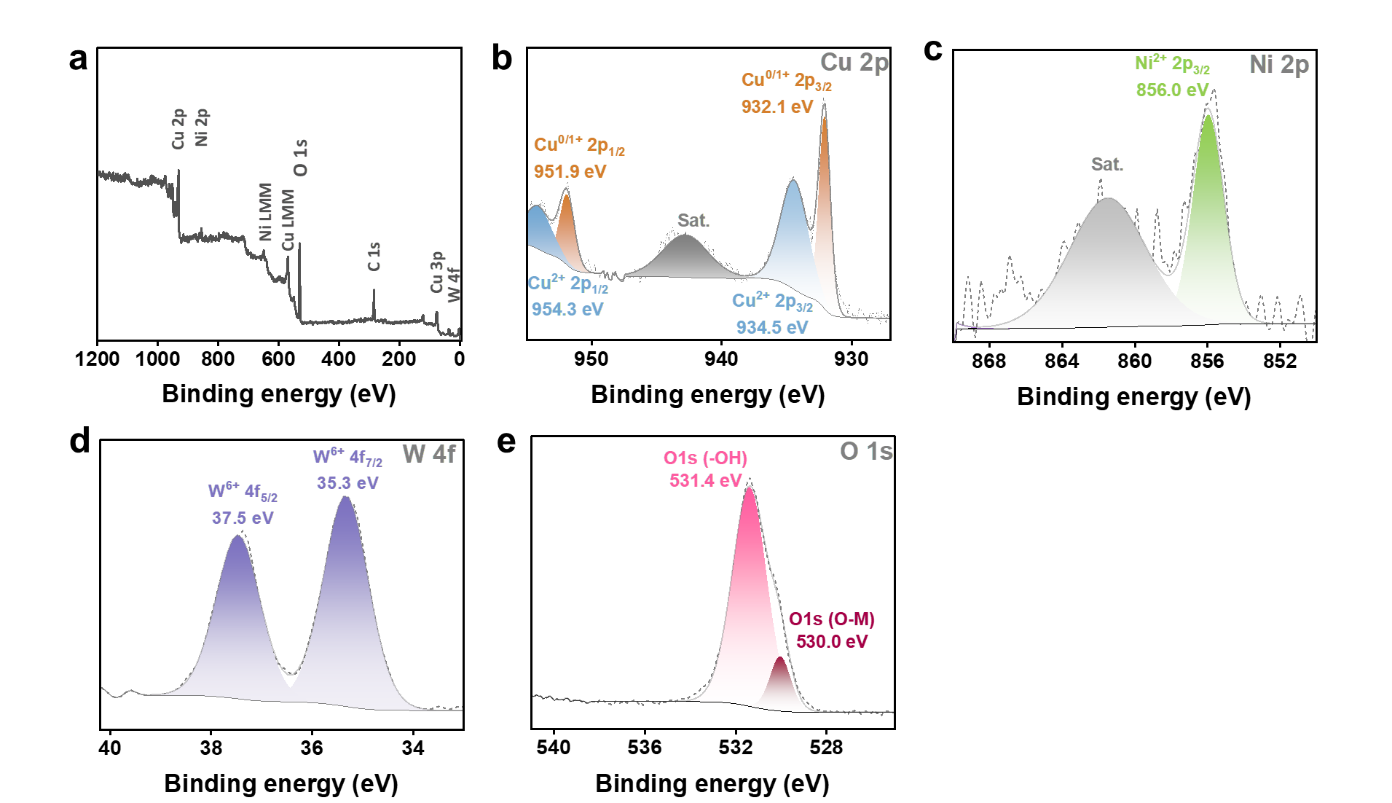


**Fig. S21**. (a) Survey spectrum and (b-e) high-resolution XPS spectra of bulk **E2**.

**
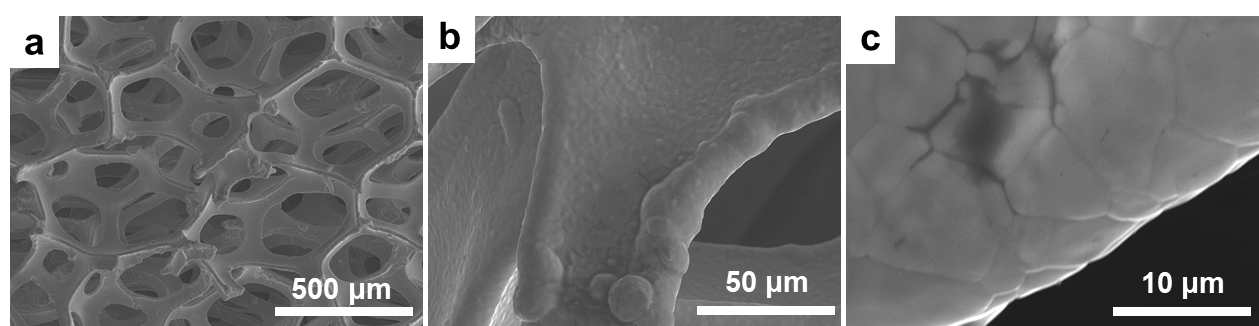
**

**Fig. S22**. SEM images of bulk **E4** at different magnifications.


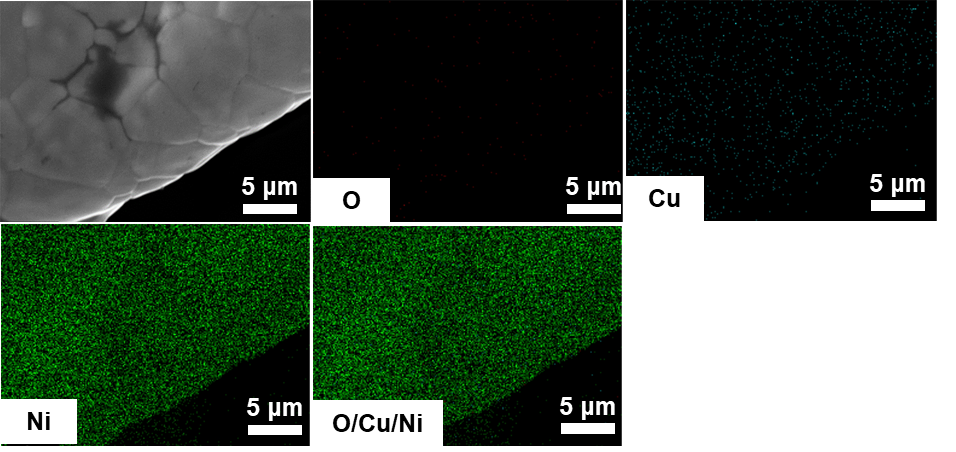


**Fig. S23**. SEM-EDX elemental mapping of bulk **E4**.


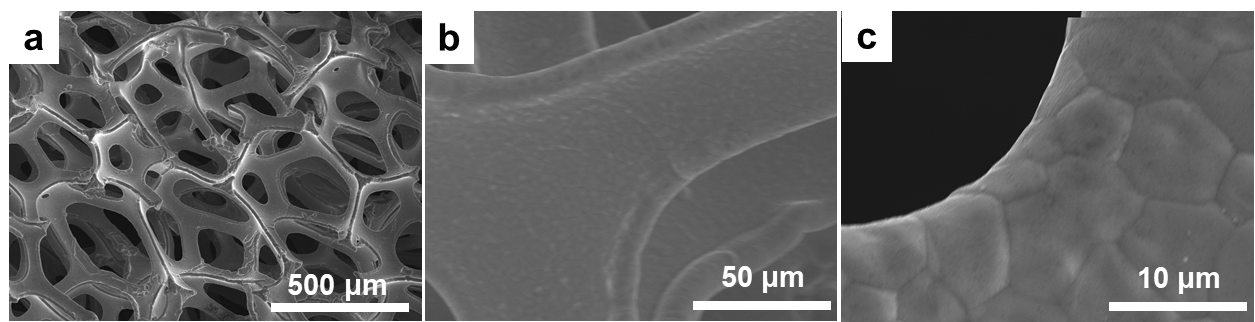


**Fig. S24**. SEM images of bulk **E5** at different magnifications.


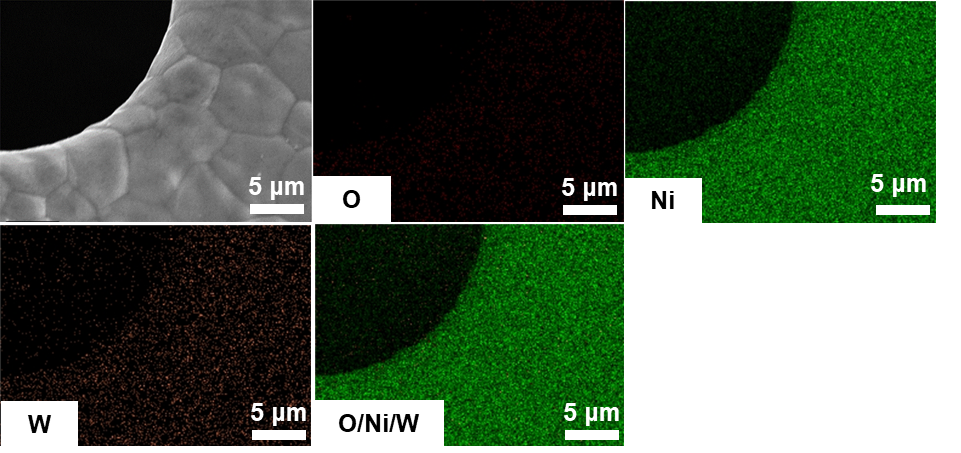


**Fig. S25**. SEM-EDX elemental mapping of bulk **E5**.


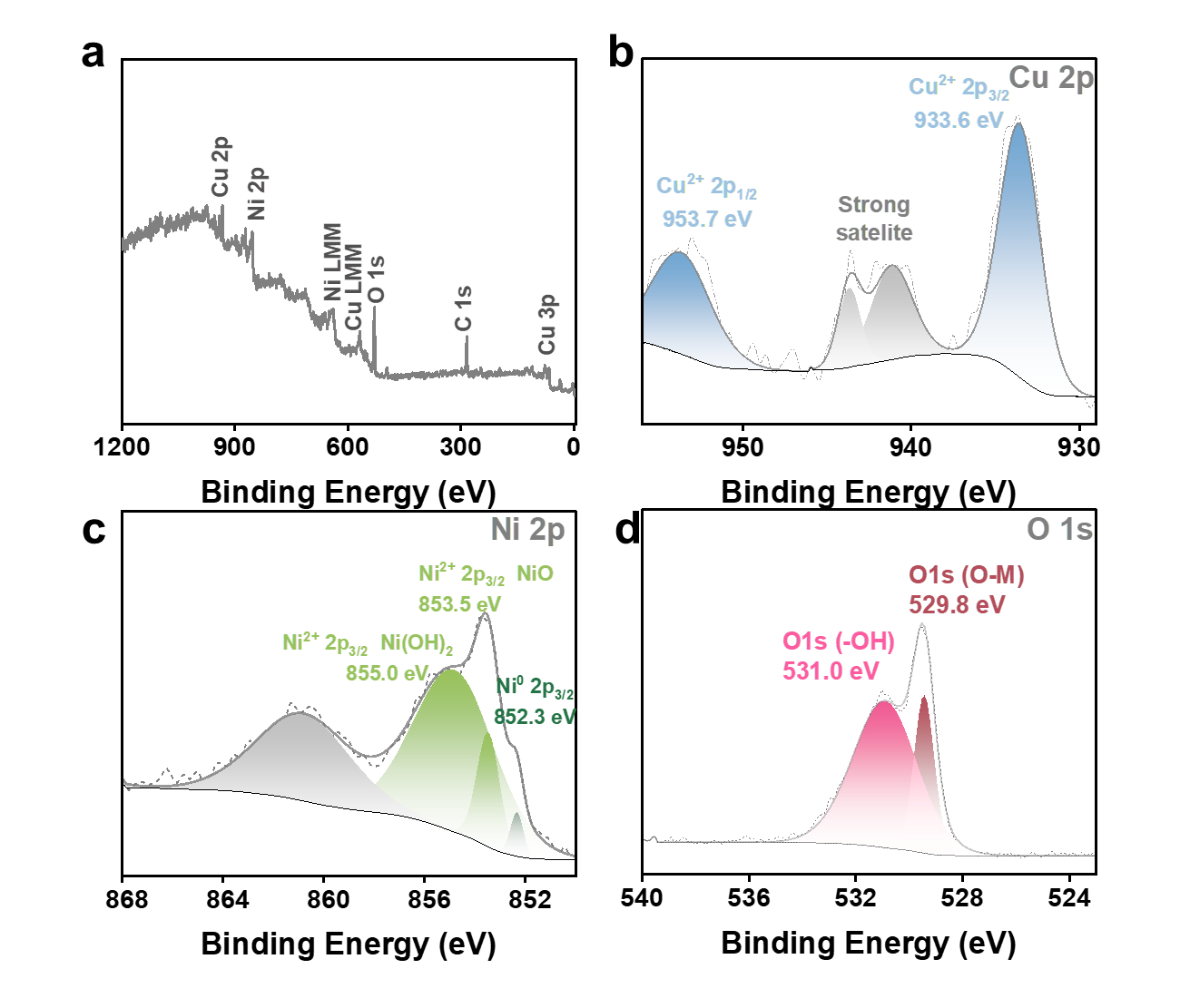


**Fig. S26**. (a) Survey spectrum and (b-d) high-resolution XPS spectra of bulk **E4**.


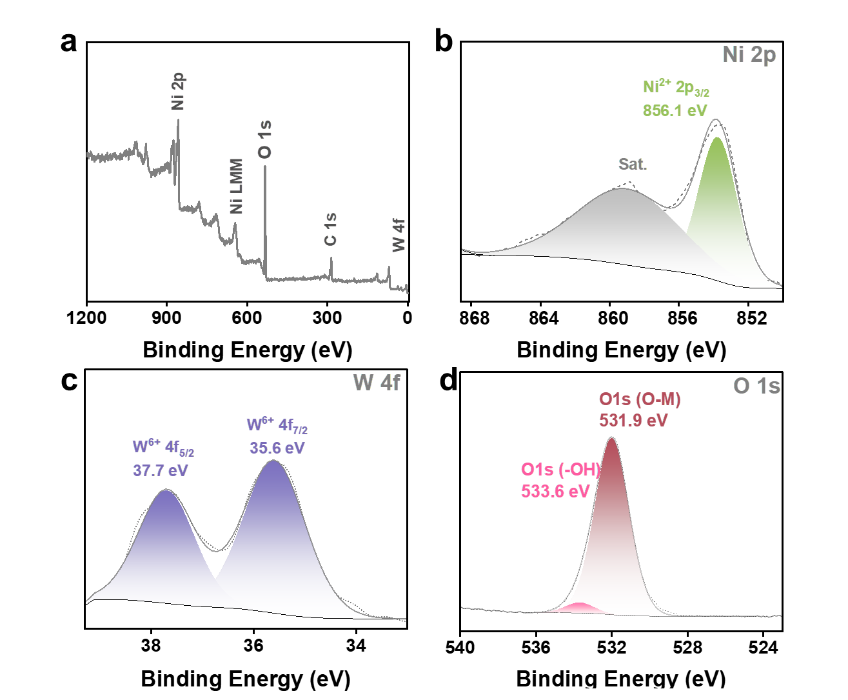


**Fig. S27**. (a) Survey spectrum and (b-d) high-resolution XPS spectra of bulk **E5**.


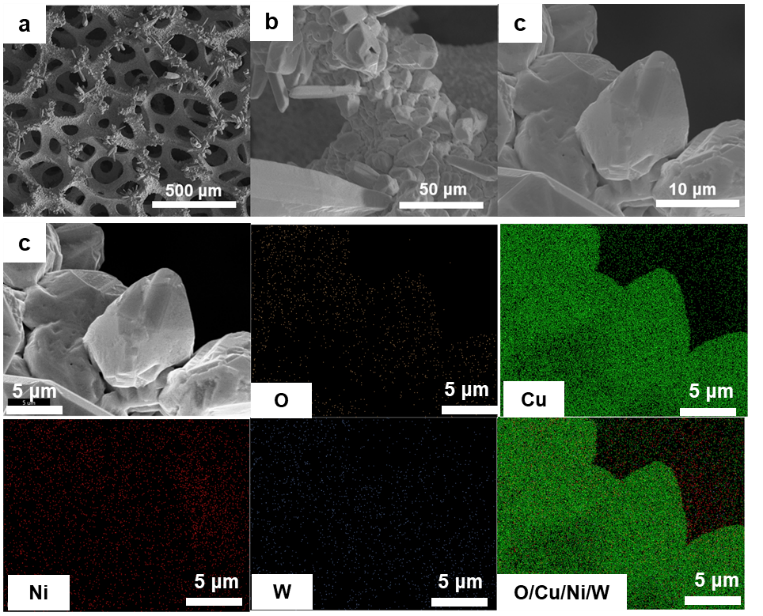


**Fig. S28.** SEM-EDX elemental mapping of bulk electrode prepared with a Cu^2+^ to [SiW_11_O_39_]^8-^ ratio of 2:1 in the precursor solution.

**
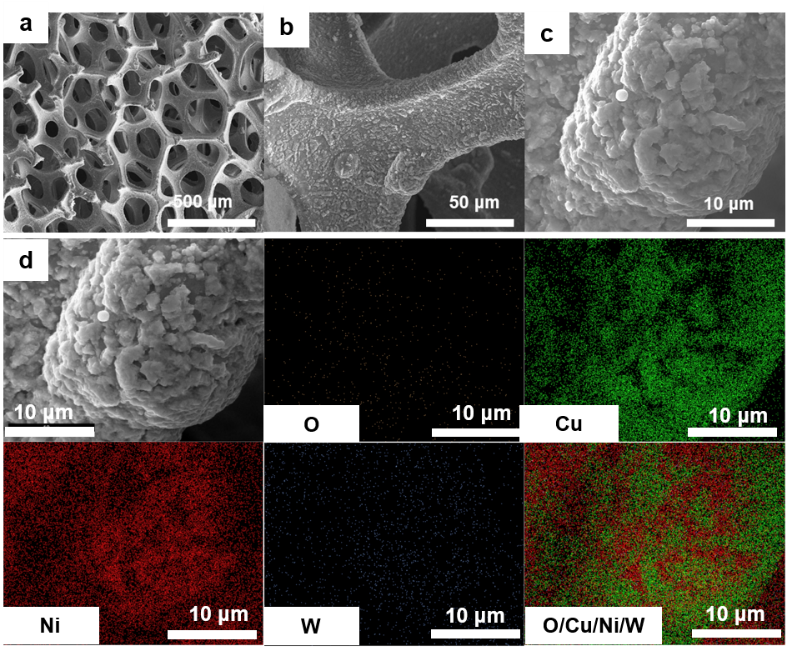
**

**Fig. S29.** SEM-EDX elemental mapping of bulk electrode prepared with a Cu^2+^ to [SiW_11_O_39_]^8-^ ratio of 1:2 in the precursor solution.

**Table S5.** Metal contents in the composite catalyst peeled off from the bulk electrode prepared with a Cu^2+^ to [SiW_11_O_39_]^8-^ ratio of **2:1** and **1:2** in the precursor solution, based on ICP-OES analyses.

|  |  | **Cu** | **Ni** | **W** |
| --- | --- | --- | --- | --- |
| **2:1** | **Mass ratio** | **29.7** | **5.75** | **1** |
|  | **Atomic ratio** | **86** | **18** | **1** |
| **1:2** | **Mass ratio** | **6.86** | **1.13** | **1** |
|  | **Atomic ratio** | **19.9** | **3.55** | **1** |

**5. Electrochemical studies**

**
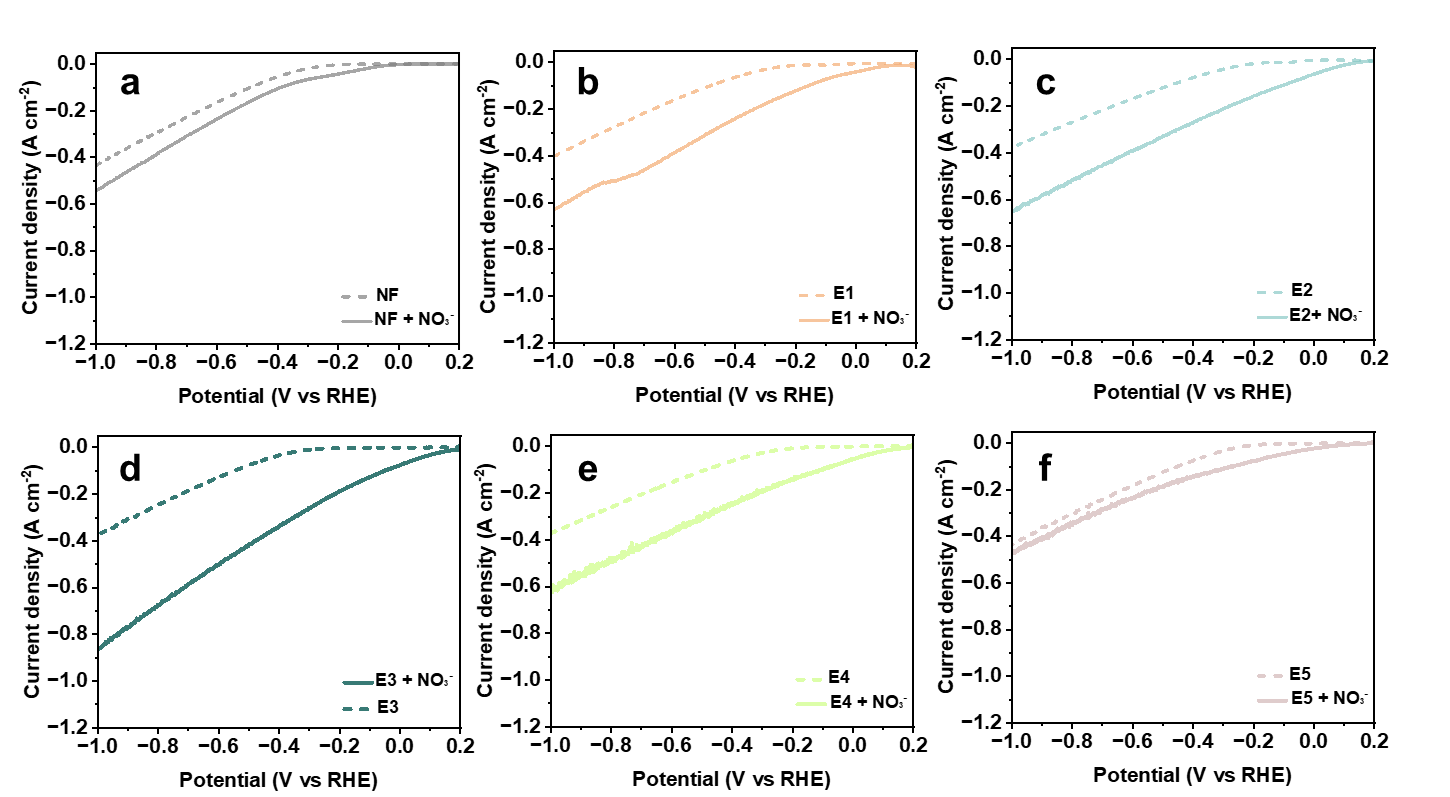
**

**Fig. S30**. LSV comparison of (a) **NF**, (b) **E1**, (c) **E2**, (d) **E3**, (e) **E4** and (f) **E5** for HER (dashed line) and NO_3_^-^RR (solid line).


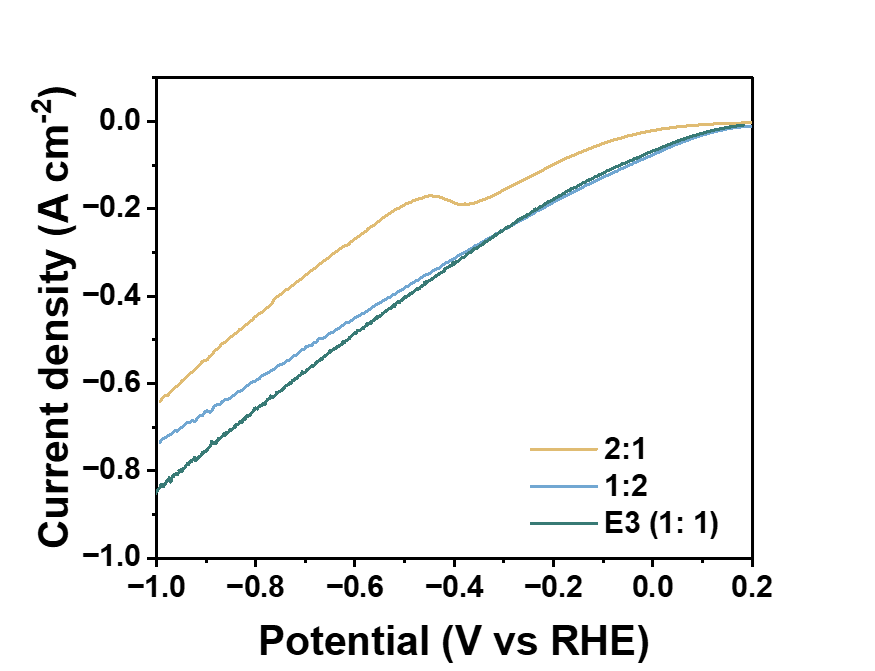


**Fig. S31**. LSV comparison of electrodes prepared with different Cu^2+^ to [SiW_11_O_39_]^8-^ ratios in the precursor solution.

**
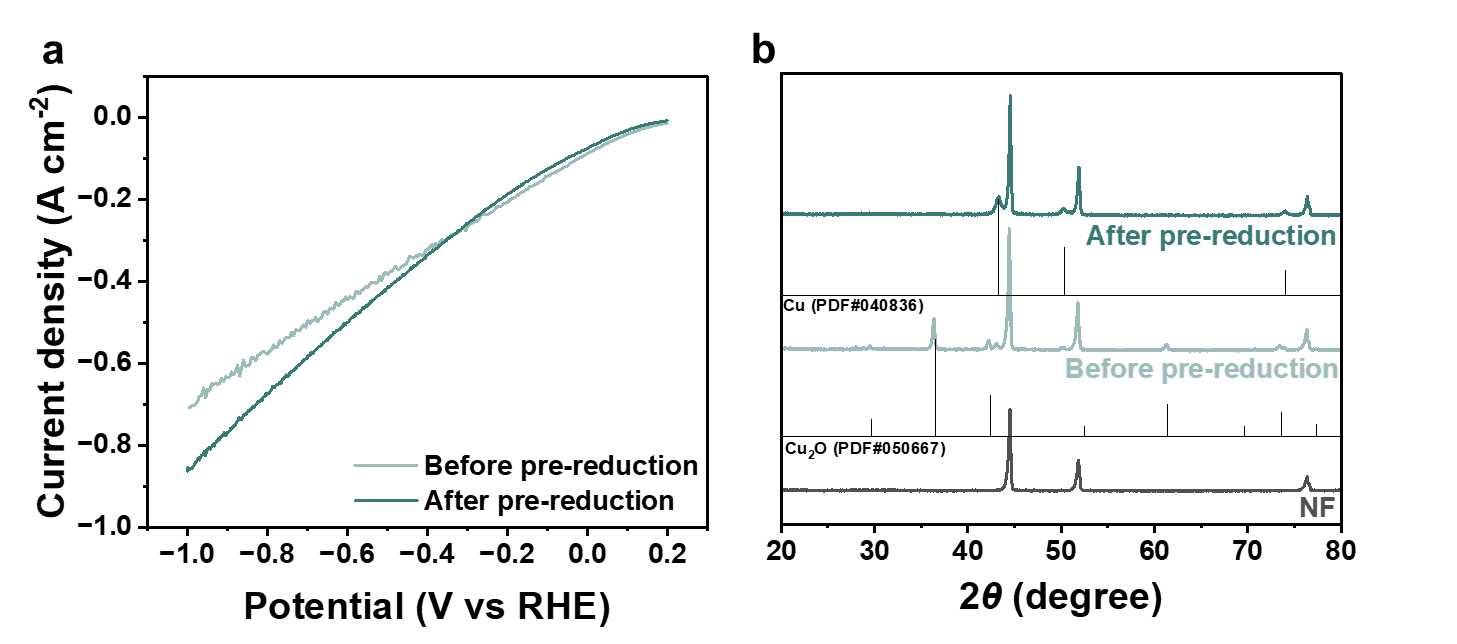
**

**Fig. S32**. (a) LSV and (b) XRD comparison before and after pre-reduction process for bulk **E3**.


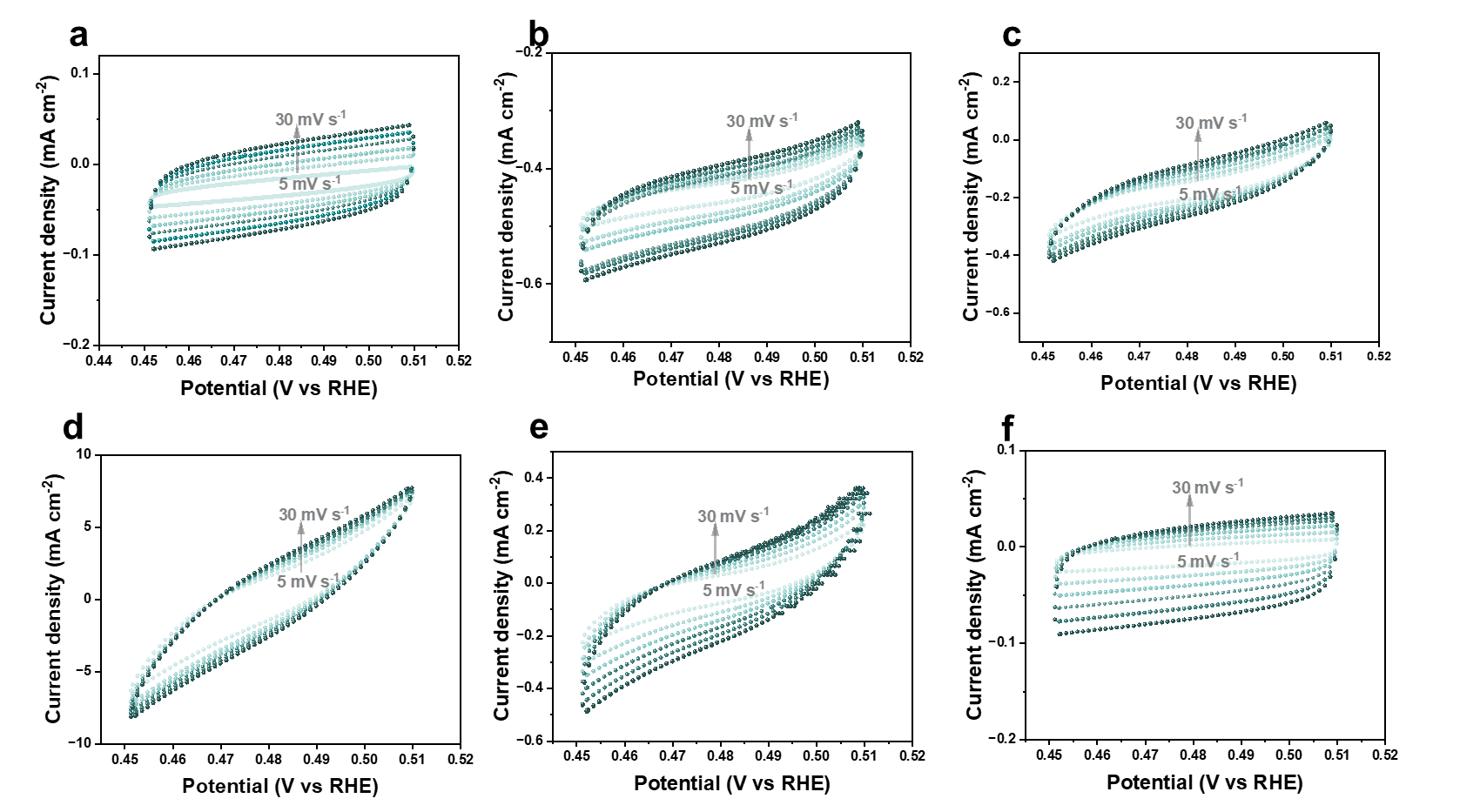


**Fig. S33.** Electrochemical capacitance measurements to determine the ECSA of (a) **NF**, (b) **E1**, (c) **E2**, (d) **E3**, (e) **E4** and (f) **E5**.


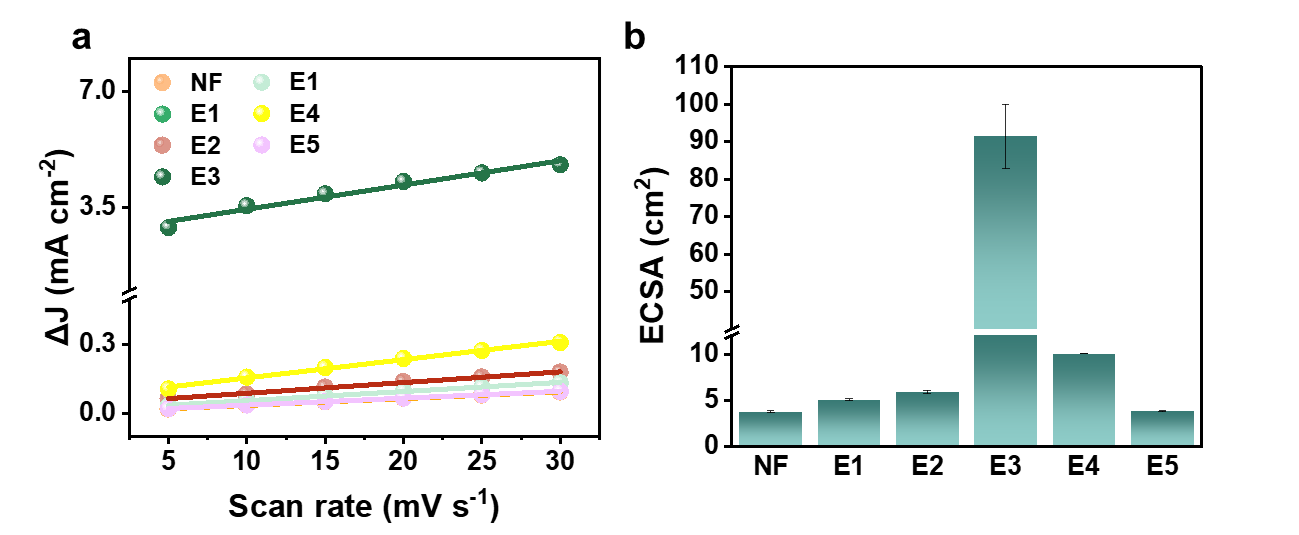


**Fig. S34.** ECSA study of **NF**, **E1** - **E5**, showing the corresponding (a) linear fits and (b) ECSA values.

**
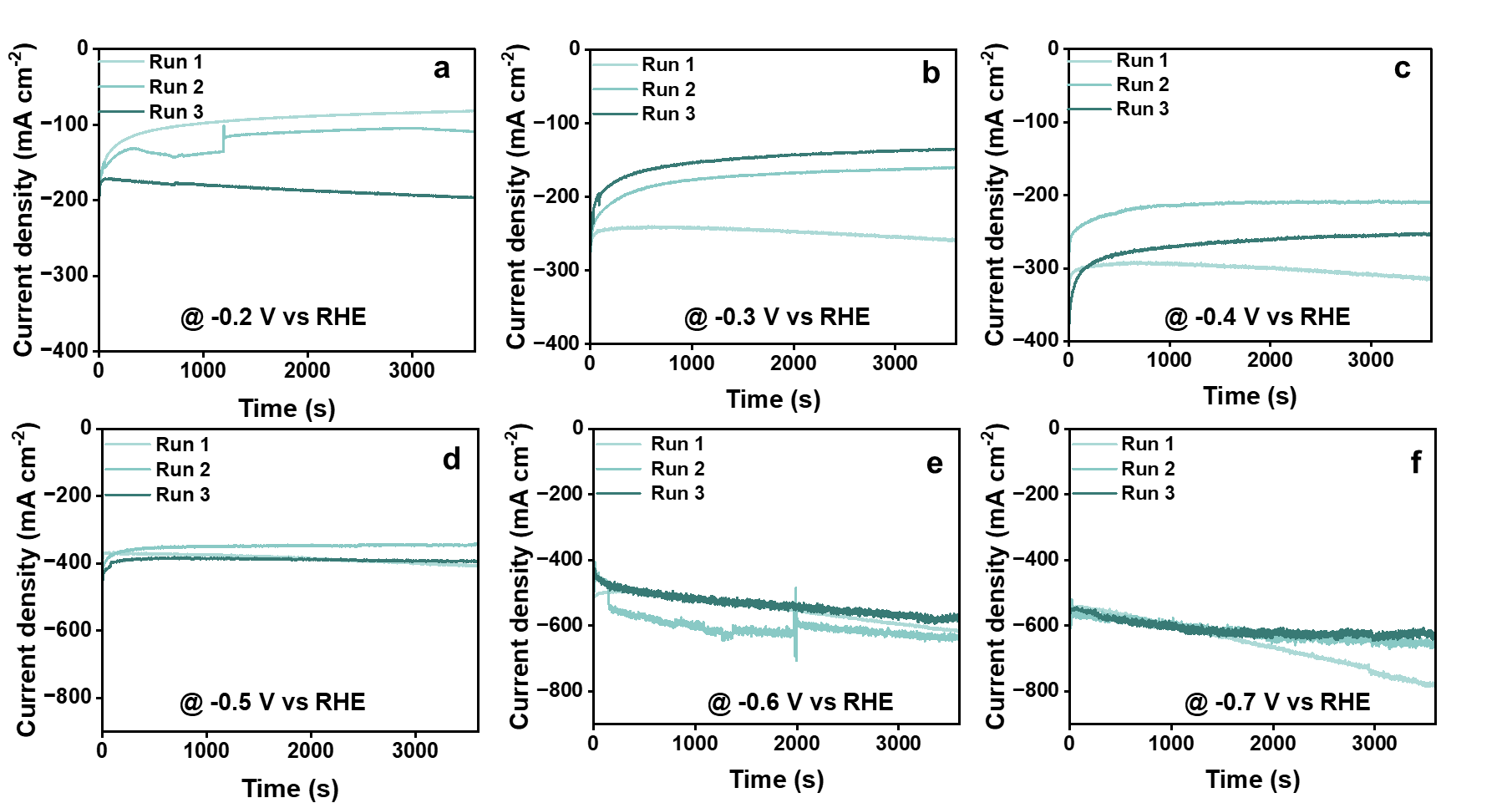
**

**Fig. S35.** CA measurements of **E3** for NO_3_^-^RR at various (static) potentials as indicated. Triplicate runs were performed for each potential.

**
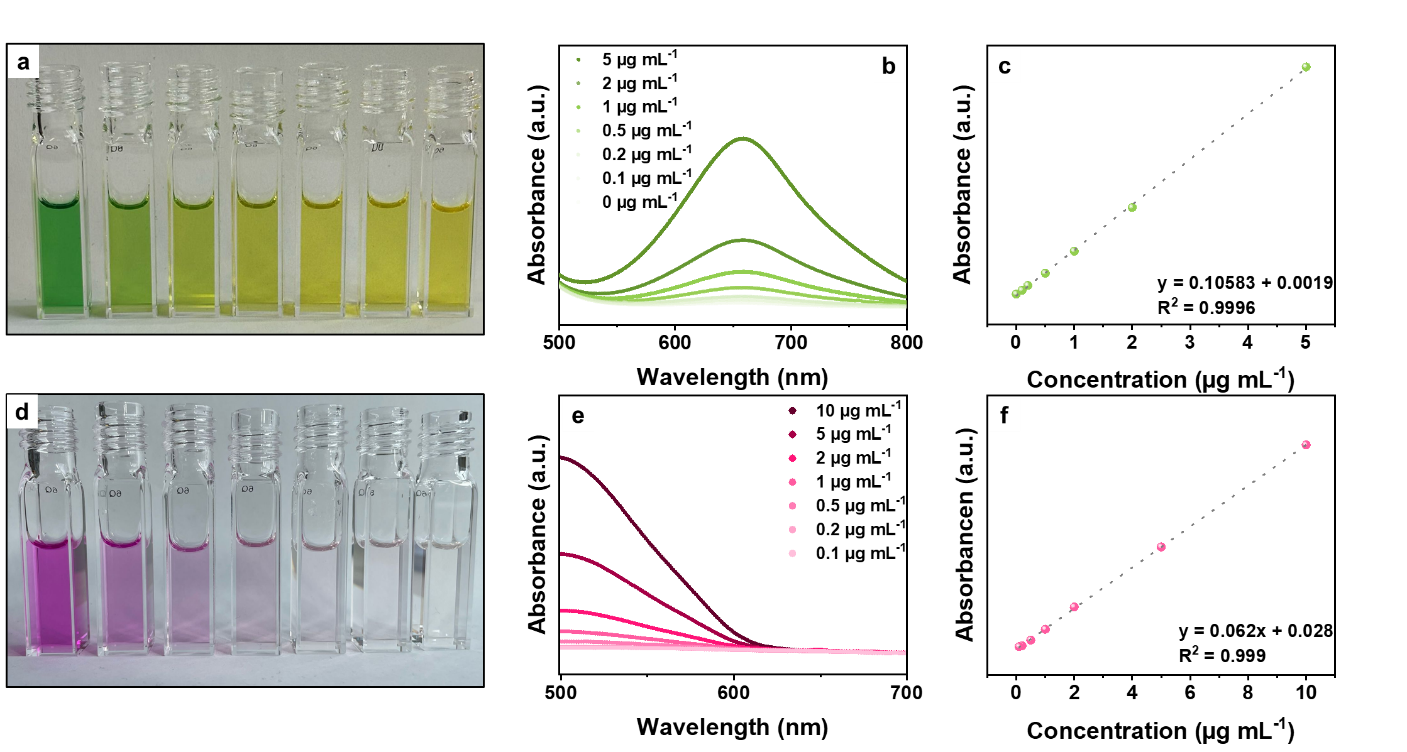
**

**Fig. S36.** (a) Gradient concentration solutions, (b) UV-Vis curves and (c) calibration curves for determining ammonia. (d) Gradient concentration solutions, (e) UV-Vis curves and (f) calibration curves for determining nitrite.

**
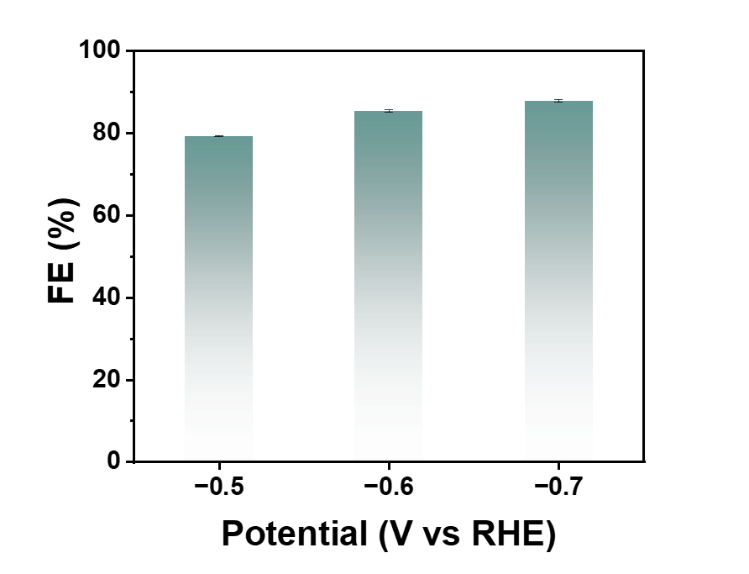
**

**Fig. S37.** NH_3_ FE of **E3** under static -0.5, -0.6, and -0.7 V vs RHE (for 1 h CA tests), quantified by three times UV-Vis spectroscopy using the same electrolyte sample.

**
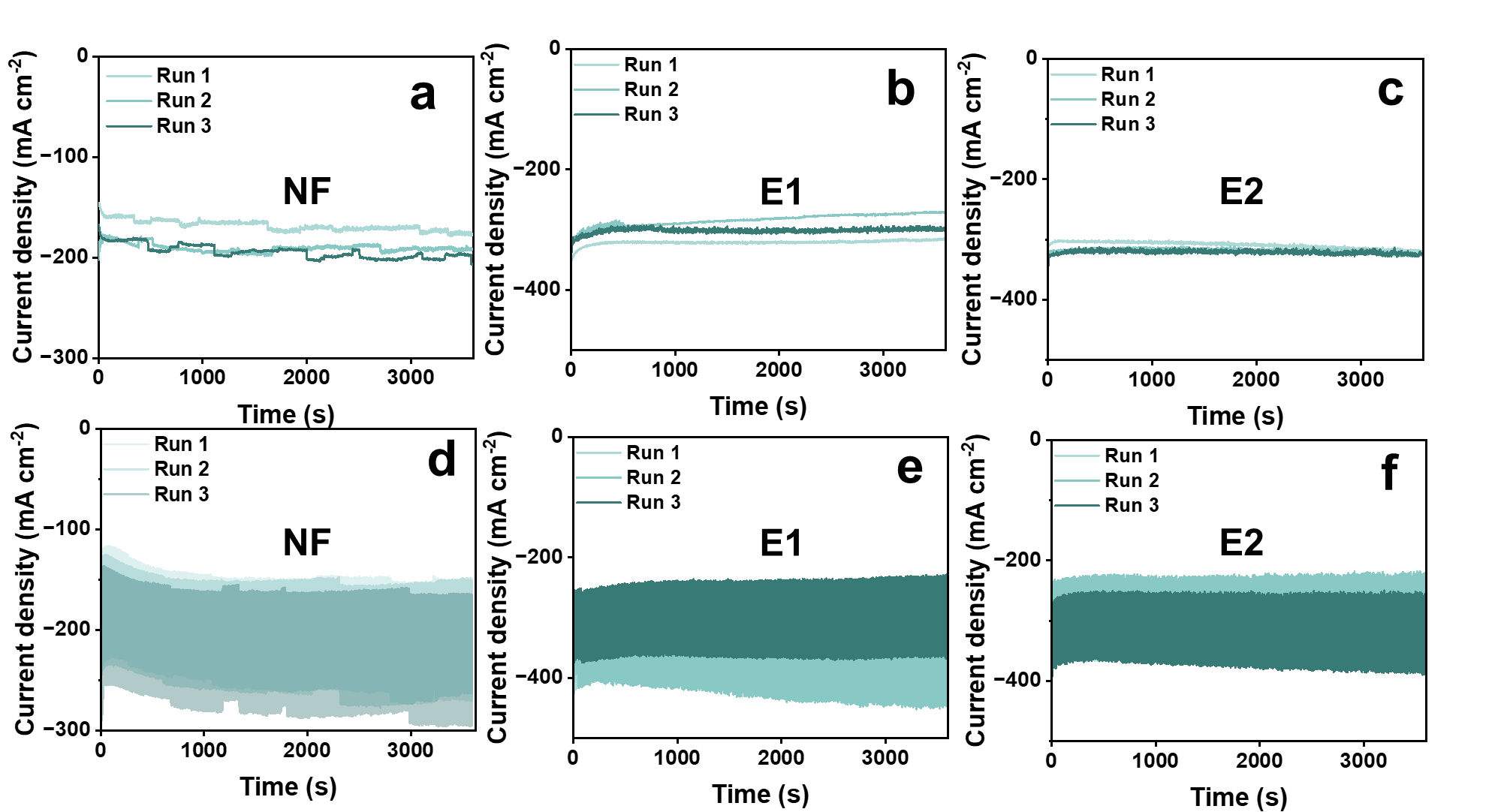
**

**Fig. S38**. CA measurements of **NF**, **E1** and **E2** for NO_3_^-^RR at (a-c) static (-0.6 V vs RHE) and (d-f) pulsed potential (-0.5 V vs RHE for 1s followed by -0.7 V vs RHE for 2s, **Fig. 3c**). Triplicate runs were performed for each experiment.


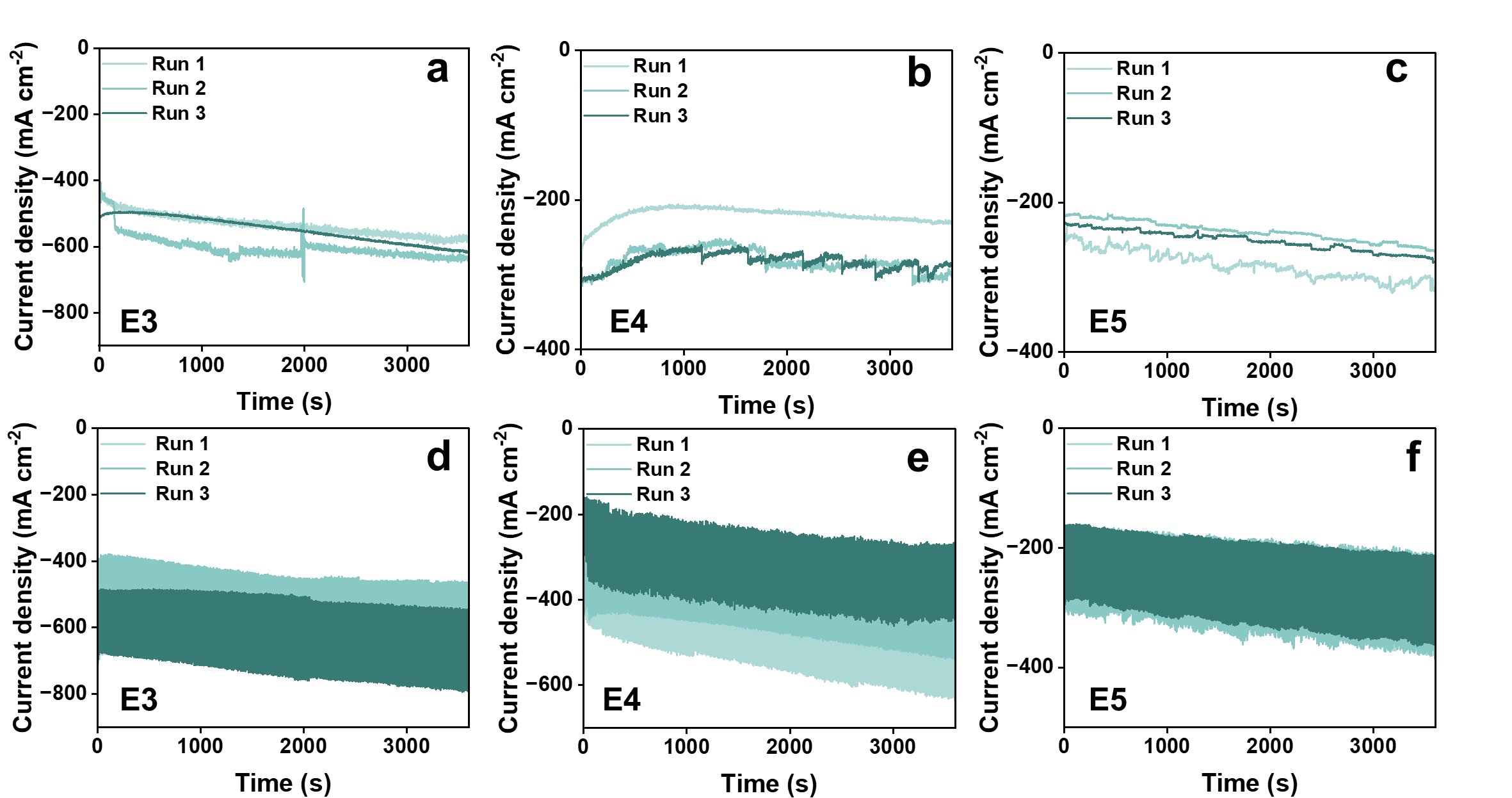


**Fig.** **S39**.CA measurements of **E3**, **E4** and **E5** for NO_3_^-^RR at (a-c) static (-0.6 V vs RHE) and (d-f) pulsed potential (-0.5 V vs RHE for 1s followed by -0.7 V vs RHE for 2s, **Fig. 3c**). Triplicate runs were performed for each experiment.


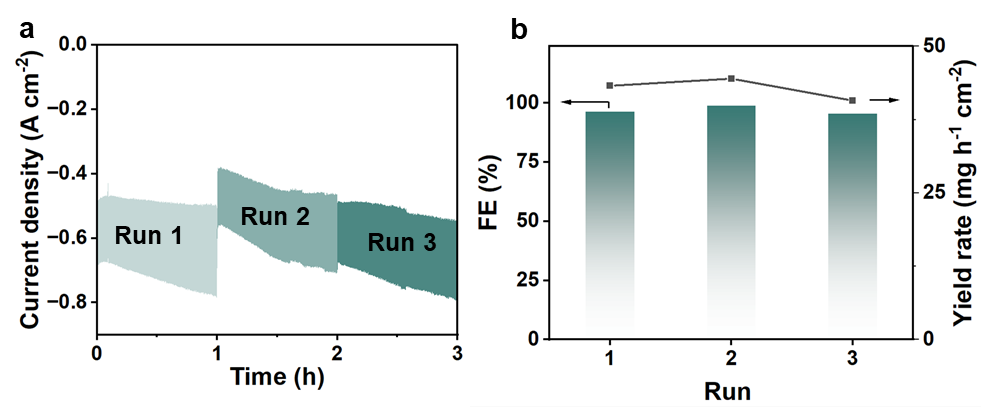


**Fig. S40**. (a) Triplicate CA of **E3** at pulsed potential (-0.5 V vs RHE for 1s followed by -0.7 V vs RHE for 2s, **Fig. 3c**), (b) corresponding NH_3_ FE and yield rate.


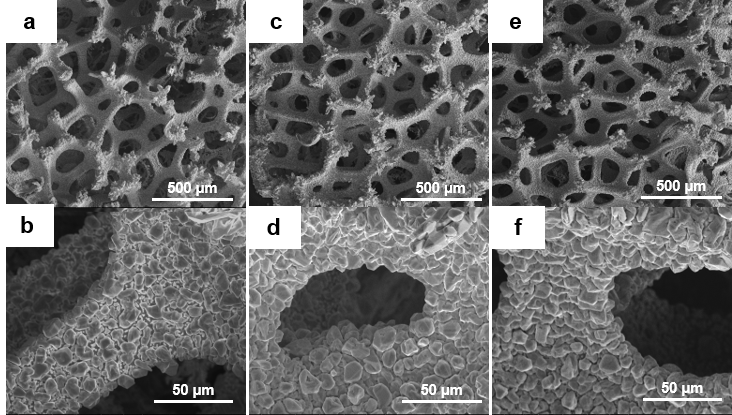


**Fig. S41**. SEM images of bulk **E3** over (a, b) Run1, (c, d) Run 2 and (e, f) Run 3.


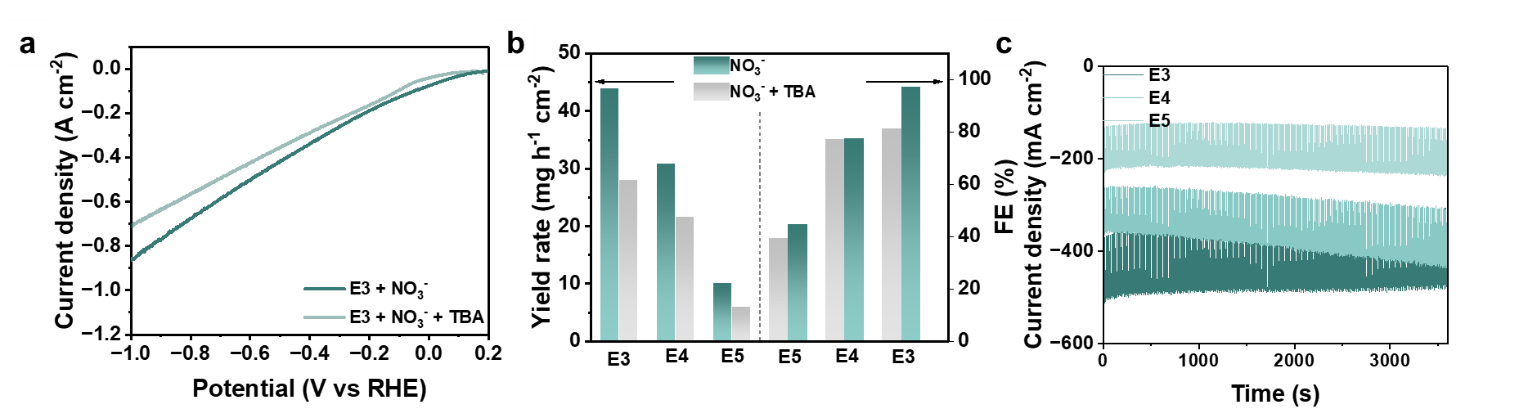


**Fig. S42.** (a) LSV, (b) FE and yield rate, (c) CA data (pulsed potential: -0.5 V vs RHE for 1s followed by -0.7 V vs RHE for 2s, **Fig. 3c**) comparison of **E3** with and without TBA (0.5 M) in the electrolyte.


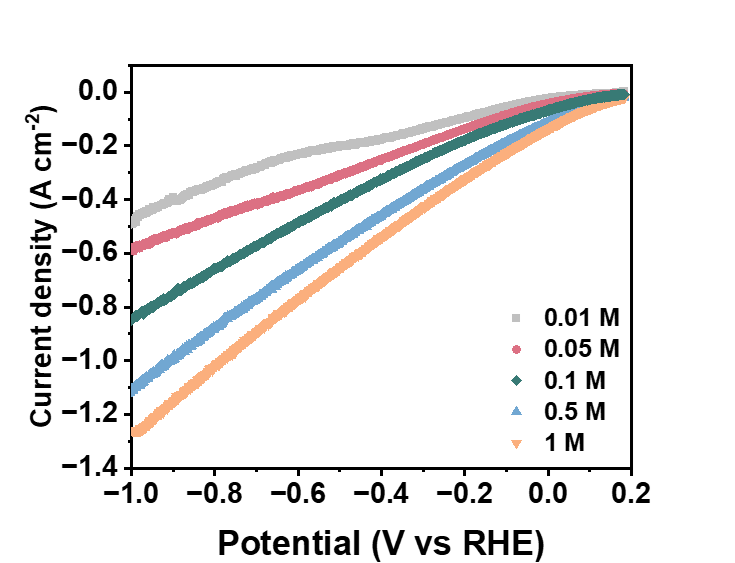


**Fig. S43**. LSV curves of **E3** in various NO_3_^-^ concentrations as indicated.


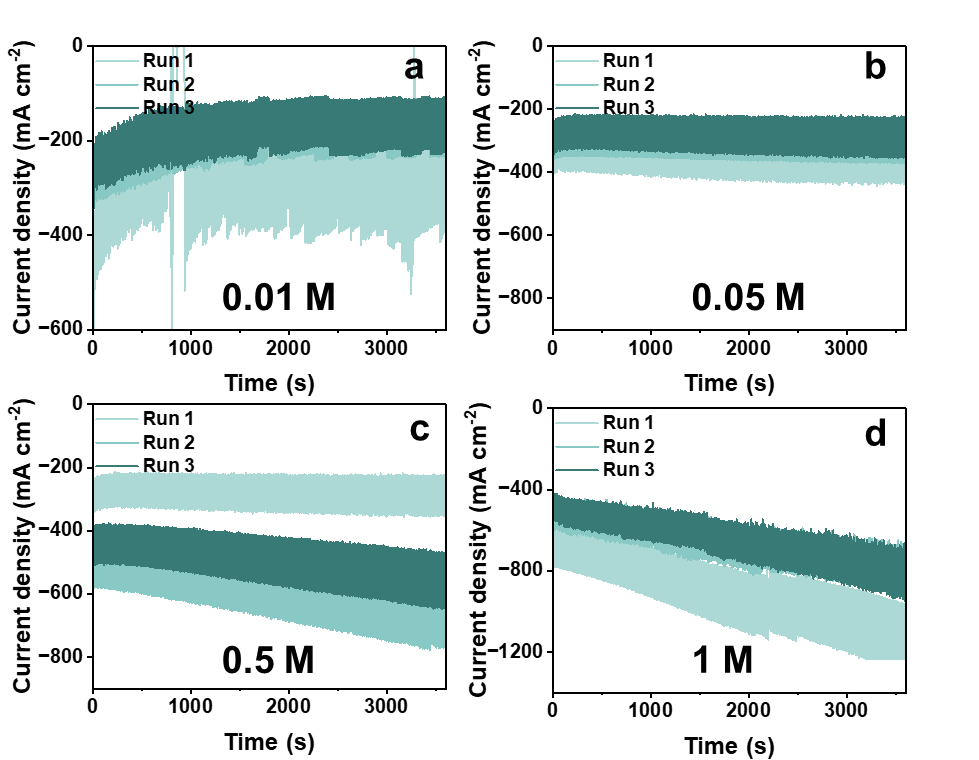


**Fig. S44**. CA measurements (pulsed potential: -0.5 V vs RHE for 1s followed by -0.7 V vs RHE for 2s, **Fig. 3c**) of **E3** for NO_3_^-^RR at various NO_3_^-^ concentrations as indicated. Triplicate runs were performed.


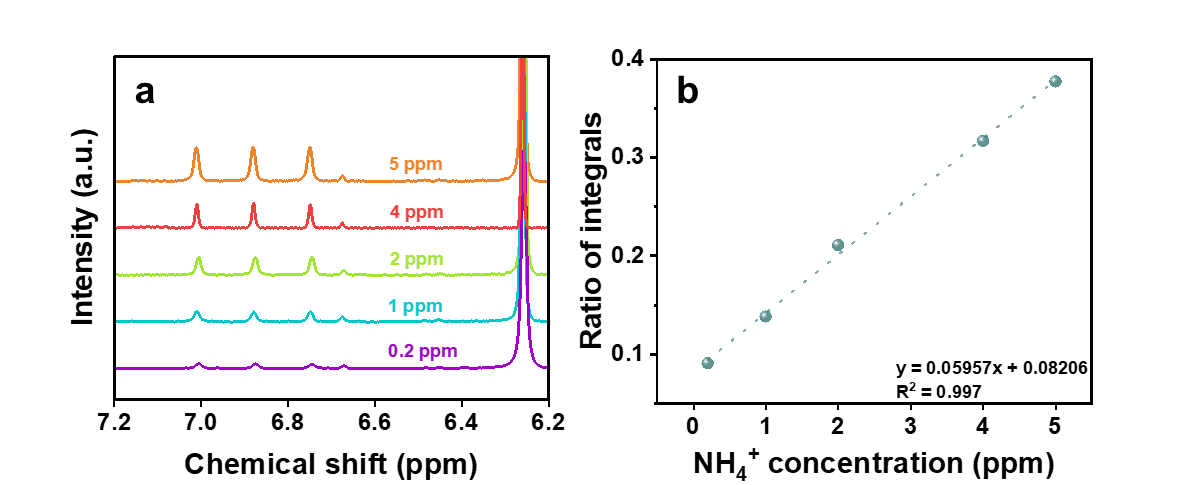


**Fig. S45**. (a) ^1^H NMR spectra and (b) calibration curves for determining ammonia product.


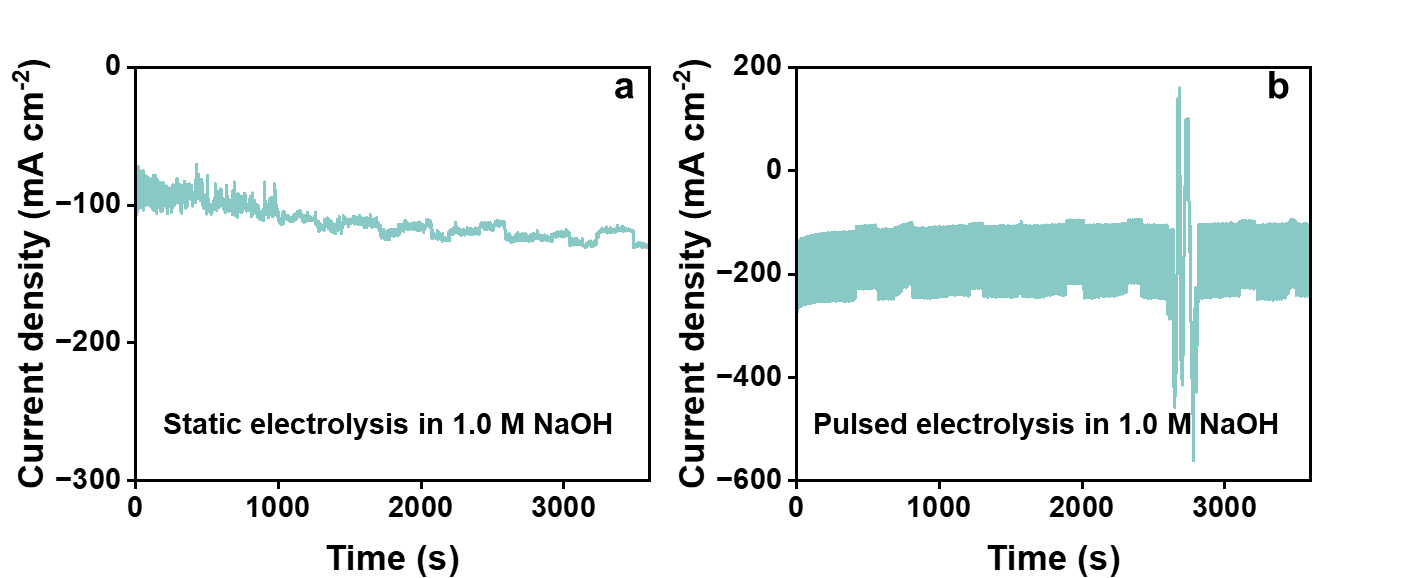


**Fig. S46**. CA measurements of **E3** at (a) static (-0.6 V vs RHE) and (b) pulsed potential (-0.5 V vs RHE for 1s followed by -0.7 V vs RHE for 2s, **Fig. 3c**) in 1 M NaOH electrolyte.


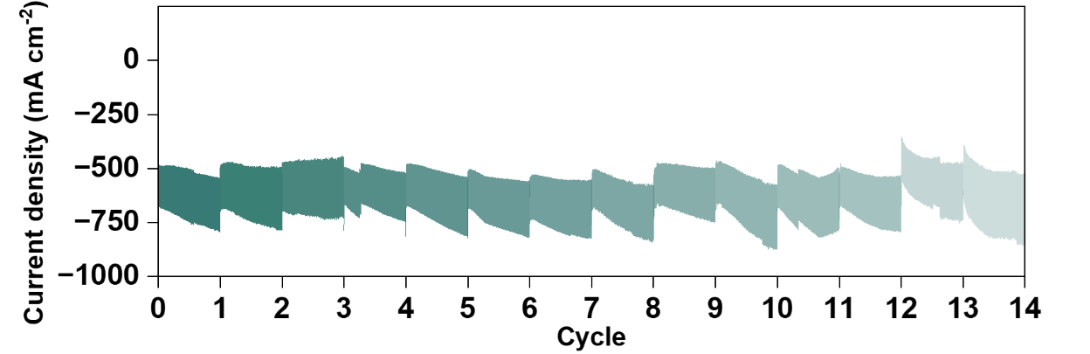


**Fig. S47**. Consecutive CA tests of **E3** under pulsed potential (-0.5 V vs RHE for 1s followed by -0.7 V vs RHE for 2s, **Fig. 3c**) for 14 cycles.


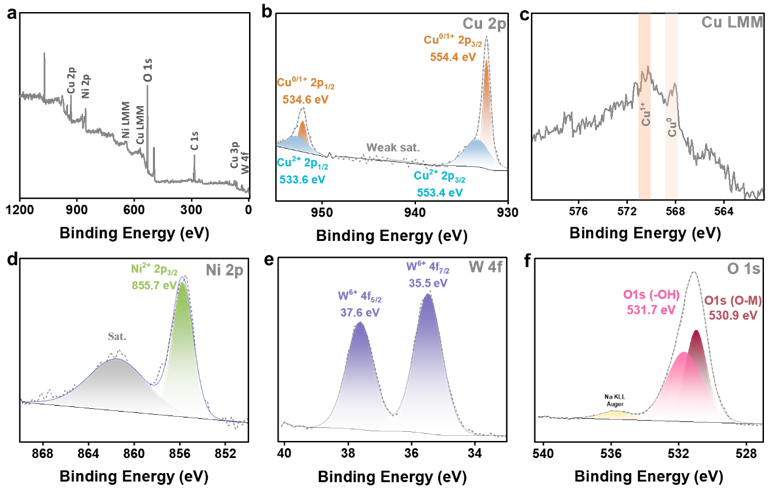


**Fig. S48.** (a) Survey spectrum and (b-f) high-resolution XPS spectra of bulk **E3** post NO_3_^-^RR (for 1 h CA).


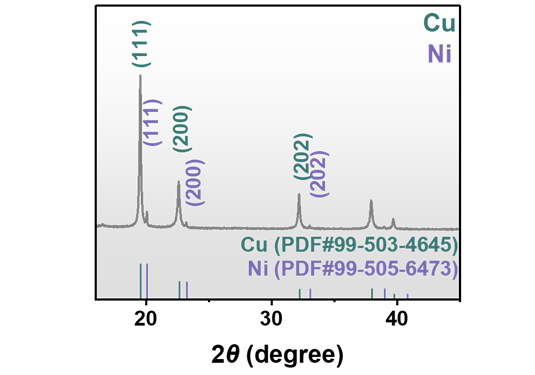


**Fig. S49.** pXRD data of catalyst recovered from **E3** after 14 cycles test. Recovery of the pure catalysts from the **NF** electrode was achieved by prolonged sonication in ethanol.


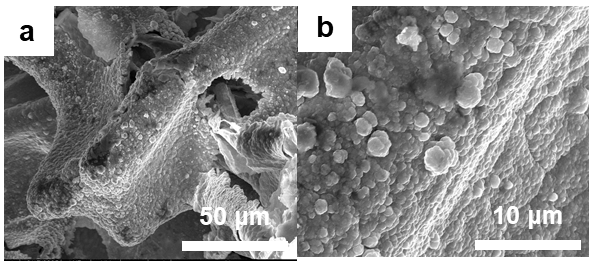


**Fig. S50.** SEM data of bulk **E3** after 14 cycles test.

**Table S6.** Cu and Ni content in the electrolyte over 14 cycles test based on ICP-OES analyses.

| **Cycle** | **Cu**  **(µg mL^-1^)** | **Ni**  **(µg mL^-1^)** |
| --- | --- | --- |
| **1** | **4.38** | **0** |
| **7** | **1.76** | **0** |
| **14** | **0.062** | **0** |

**
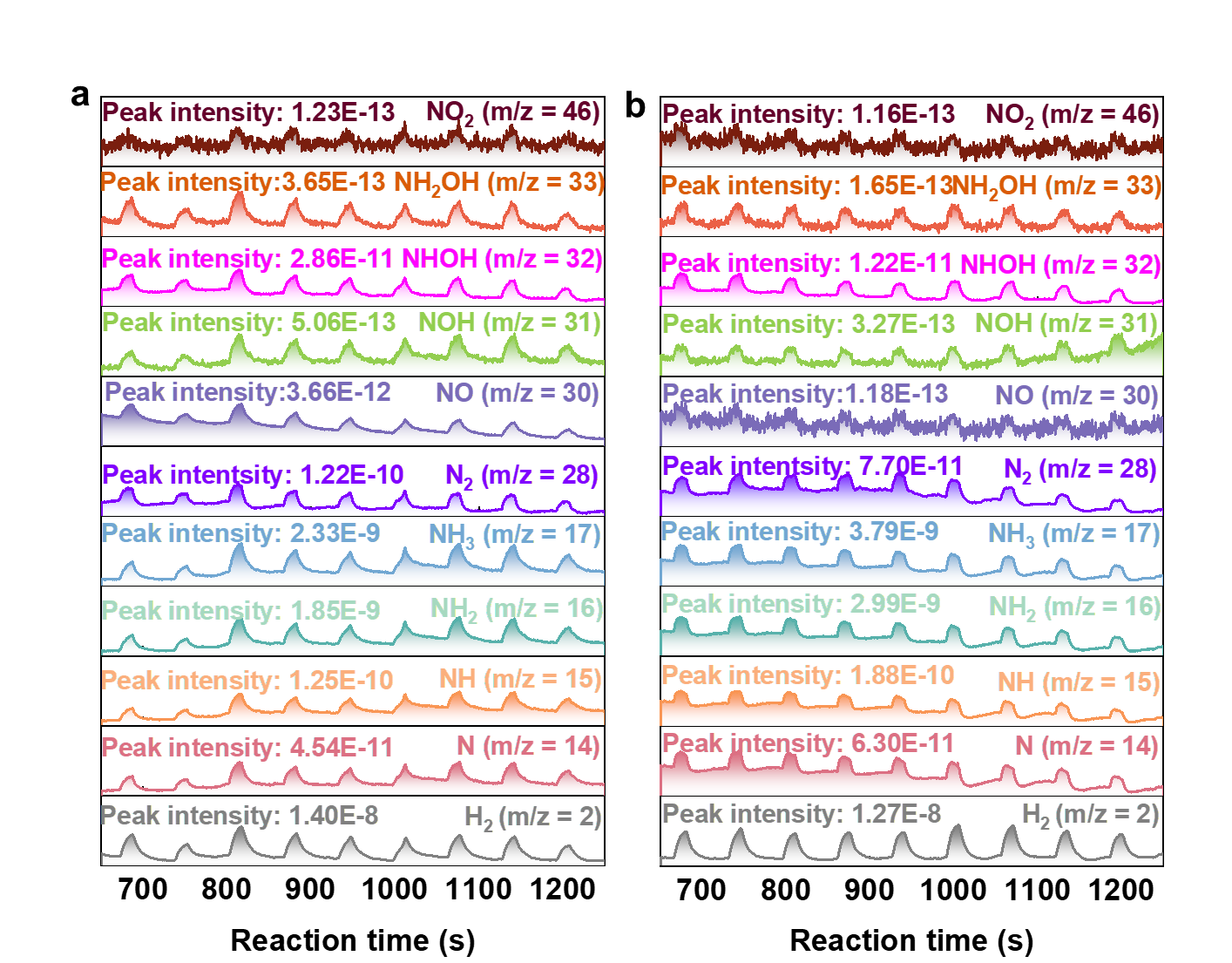
**

**Fig. S51.** DEMS data of (a) **E4** and (b) **E5** under pulsed (-0.5 V vs RHE for 1s followed by -0.7 V vs RHE for 2s) electrolysis.

**Table S7.** Comparison of catalytic performance of **E3** and recently reported Cu-, Ni-, and WO_3_-based NO_3_^-^RR electrocatalysts under alkaline media.

| **Electrocatalyst** | **Yield rate**  **(mg h^−1^ cm^−2^)** | **Faradic efficiency (%)** | **Alkaline electrolyte**  **concentration (M)** | **NO_3_^-^**  **concentration (M)** | **Applied**  **potential**  **(V vs RHE)** | **Data source (Year)** |
| --- | --- | --- | --- | --- | --- | --- |
| **Cu-Cu_2_O/Ni-NiO/WO_3_@NF** | **43.87** | **97.2** | **1** | **0.1** | **Pulsed:**  **-0.5 - -0.7** | **This work** |
| Ru-Tta-Dfp COF | 1.16 | 93.93 | 1 | 0.1 | -0.4 | *ACS Energy Lett.* (2024)^[15]^ |
| Cu_2_O | 19.72 | 97.64 | 1 | 0.029 | -0.5 | *Energy Environ. Sci*. (2023)^[16]^ |
| Cu-NiCo/NF | 12.48 | 97.9 | 1 | 0.1 | -0.15 | *Angew. Chem. Int. Ed*. (2024)^[17]^ |
| Cu@CoCu LDH/CC | 10.10 | 96.2 | 1 | 0.1 | -0.3 | *ACS Catal*. (2024)^[18]^ |
| Co@CF | 7.51 | 95.16 | 0.1 | 0.1 | -0.8 | *J. Mater. Chem. A* (2025)^[19]^ |
| P-Cu/Co(OH)_2_ | 42.63 | 97.04 | 1 | 0.1 | −0.4 | *Adv. Mater*. (2024)^[20]^ |
| Cu/Cu*_x_*O/GDY | 25.4 | 99.8 | 1 | 0.1 | -0.8 | *Adv. Mater*. (2024)^[21]^ |
| Cu-CoO/Co(OH)_2_ | 7.65 | 94.7 | 1 | 0.1 | -0.5 | *Adv. Energy Mater*. (2024)^[22]^ |
| BCN@Cu | 7.84 | 88.9 | 0.1 | 0.1 | -0.6 | *J. Mater. Chem. A* (2021)^[23]^ |
| Cu-RD-KOH |  | 96.5 | 0.1 | 0.005 | -0.3 | *Angew. Chem. Int. Ed*. (2023)^[24]^ |
| Cu nanosheets | 23.97 | 88 | 1 | 0.2 | -0.59 | *Angew. Chem. Int. Ed*. (2023)^[25]^ |
| Cu_50_Co_50_ | 81.6 | 100 | 1 | 0.1 | -0.2 | *Nat. Commun*. (2022)^[26]^ |
| CuO NWAs@Co_3_O_4_ | 32.55 | 99.17 | 1 | 0.1 | −0.23 | *Green Energy Environ*. (2023)^[27]^ |
| Cu-Fe_3_O_4_-5 | 7.04 | ~100 | 0.1 | 0.1 | -0.6 | *Nano Lett*. (2023)^[28]^ |
| MP-Cu | 1.292 | 99.8 | 1 | 0.05 | -0.3 | *Adv. Funct. Mater*. (2023)^[29]^ |
| Cu@NF | 4.284 | 95.05 | 1 | 0.014 | -0.23 | *J. Power Sources* (2021)^[30]^ |
| Ni-MOF-Ru-5 | 22.27 | 91.5 | 1 | 1 | −0.6 | *ACS Catal*. (2024) ^[31]^ |
| Ni_1_Cu SAAO | - | ~80 | 1 | 0.1 | +0.1 | *J. Am. Chem. Soc*. (2024)^[32]^ |
| B-Cu_2_O/Cu/CP | 2.44 | 92.74 | 0.1 | 0.016 | -0.5 | *Adv. Funct. Mater*. (2024)^[33]^ |
| Cu_x_Ru_y_ | 8.33 | >95 | 0.1 | 0.5 | -0.4 | *ACS Catal*. (2024) ^[34]^ |
| Co@Cu | 6.99 | 97.67 | 0.5 | 0.014 | -0.2 | *Chem Catal*. (2025) ^[35]^ |
| Cu_5_/Mo_0.6_-WO_3_ | 26.25 | 98.6 | 0.1 | 0.1 | -0.7 | *Adv. Funct. Mater*. (2025)^[36]^ |
| NF/Ni_3_N-Cu | 20.25 | 98.7 | 1 | 0.1 | -0.3 | *Angew. Chem. Int. Ed*. (2025)^[37]^ |
| Fe_80_Ni_20_ | 3.99 | 97.28 | 0.1 | 0.01 | -0.638 | *Angew. Chem. Int. Ed*. (2025)^[38]^ |
| CuPd/CuO@NF | 6.98 | 97.8 | 1 | 0.0071 | -0.23 | *Adv. Funct. Mater*. (2025)^[39]^ |
| Cr-Cu_2_O@CF | 11.15 | 91.6 | 1 | 0.1 | -0.175 | *Angew. Chem. Int. Ed*. (2024)^[40]^ |

**Table S8.** Comparison of catalytic performance of **E3** and recently reported NO_3_^-^RR electrocatalysts by pulsed electrolysis under alkaline media.

| **Electrocatalyst** | **Pulse profile** | | **Static potential (V vs RHE)** | **Performance under pulsed electrolysis** | | **Performance under static potential** | | **Alkaline electrolyte**  **concentration (M)** | **NO_3_^-^**  **concentration (M)** | **Cycles (under pulsed conditions)** | **Data source (Year)** |
| --- | --- | --- | --- | --- | --- | --- | --- | --- | --- | --- | --- |
|  | E_low_ (V vs RHE)  / t_ca_ (s) | E_high_ (V vs RHE)  / t_an_ (s) |  |  |  |  |  |  |  |  |  |
|  |  |  |  | FE (%) | YR (mg h^−1^ cm^−2^) | FE (%) | YR (mg h^−1^ cm^−2^) |  |  |  |  |
| **Cu-Cu_2_O/Ni-NiO/WO_3_@NF** | **-0.7 / 2** | **-0.5 / 1** | **-0.6** | **97.1** | **43.87** | **87** | **37.36** | **1** | **0.1** | **14** | **This work** |
| Co_3_O_4_/CoOOH from Co-Ni | -0.9 / 5 | 1.2 / 5 | -0.9 | 92.6 | 25.52 | 94 | 11.54 | 1 | 0.1 | N.A. | *Environ. Sci. Technol*. (2024)^[41]^ |
| Co-based catalysts | -0.5 / 5 | 0.4 - 1.4 / 5 | -0.5 | 91.7 | 23.8 | 52.5 | 3.05 | 1 | 0.1 | N.A. | *Nano Lett.* (2024)^[42]^ |
| Cu_2_O@Pd | -0.48 / 8 | 0.12 / 5 | -0.53 | 81.2 | 1.08 | 55.34 | 0.72 | 0.1 | 0.01 | N.A. | *J. Hazard. Mater*. (2024) ^[43]^ |
| (Co_0.83_Ni_0.16_)_2_Fe LDOs | -0.42 / 20 | 1.24 / 0.2 | -0.42 | 97.8 | 50.4 | 90 | 41.42 | 1 | 0.1 | N.A. | *Angew. Chem. Int. Ed*. (2024)^[44]^ |
| Cu_x_Ru_y_ | -0.2 / 9.5 | 0.6 / 1 | -0.4 | 94.94 | 2.40 | - | 0.68 | 0.1 | 0.01 - 0.5 | N.A. | *ACS Catal*. (2024)^[45]^ |
| Cu@Co/NC | -0.3 / 2 | 0.1 / 1.5 | - | 98.32 | 12.75 | - | - | 0.1 | 0.01 | 12 | *Angew. Chem. Int. Ed*. (2025)^[46]^ |
| CuNW/Cu foam | -0.6 - -0.7 / 20 | 0.3 / 1 | -0.7 | 90 | - | 25.09 | - | 1 | 0.01 | 5 | *Adv. Sci.* (2025)^[47]^ |

**6. Theoretical studies**


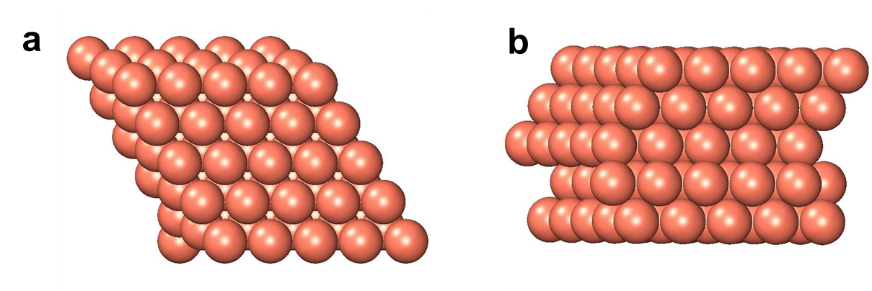


**Fig. S52.** (a) Top view and (b) side view of Cu (111) surface model.


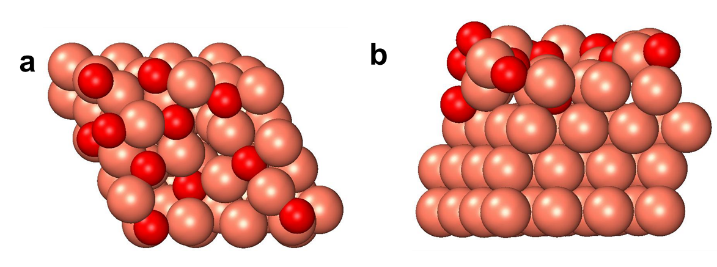


**Fig. S53.** (a) Top view and (b) side view of Cu-Cu_2_O surface model.


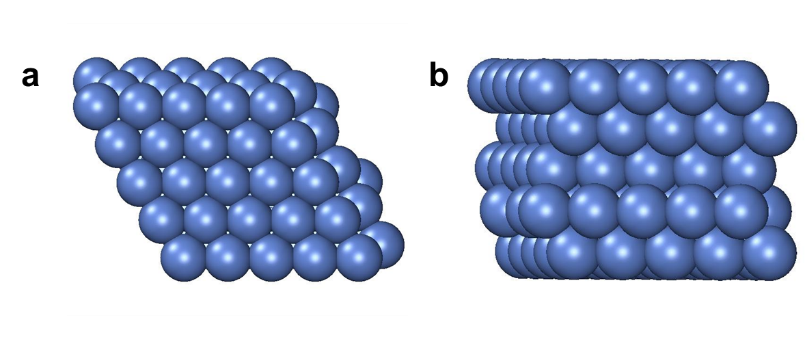


**Fig. S54.** (a) Top view and (b) side view of Ni (111) surface model.


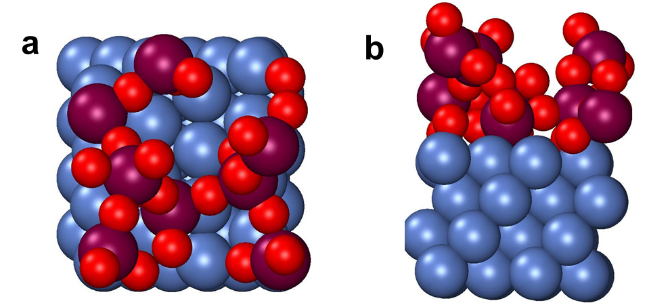


**Fig. S55.** (a) Top view and (b) side view of Ni-WO_3_ surface model.


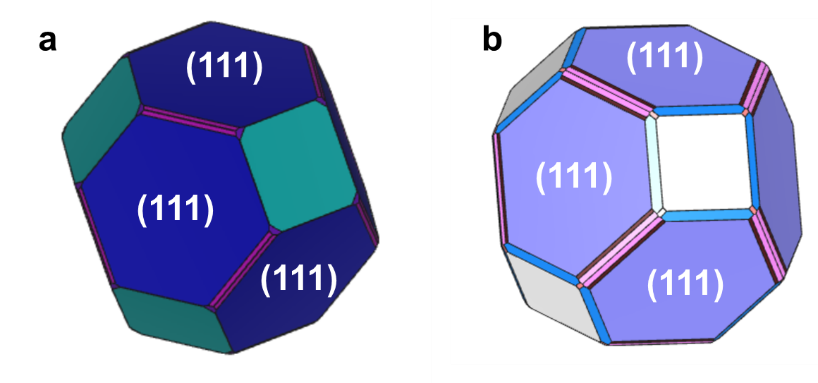


**Fig. S56.** Wulff constructions of (a) Cu and (b) Ni crystal.

**7. Analytical section of adaptive electrode for glycerol valorization**

**
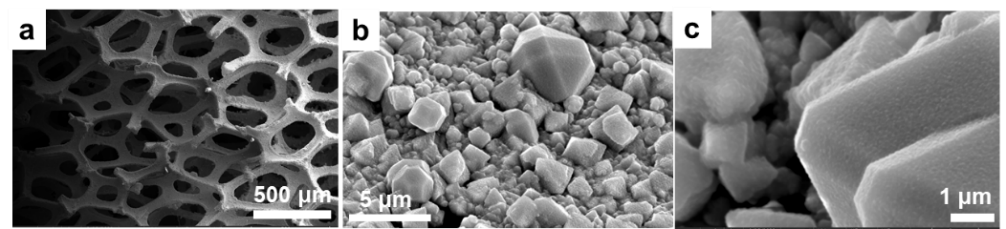
**

**Fig. S57.** SEM images of bulk **E6** at different magnifications.


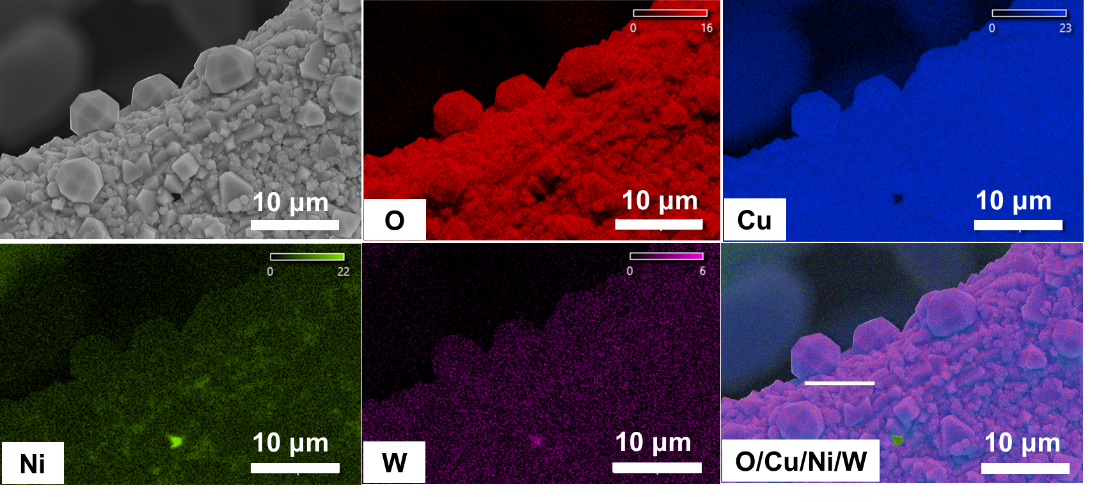


**Fig. S58.** SEM image and corresponding SEM-EDX mapping of bulk **E6**.

**
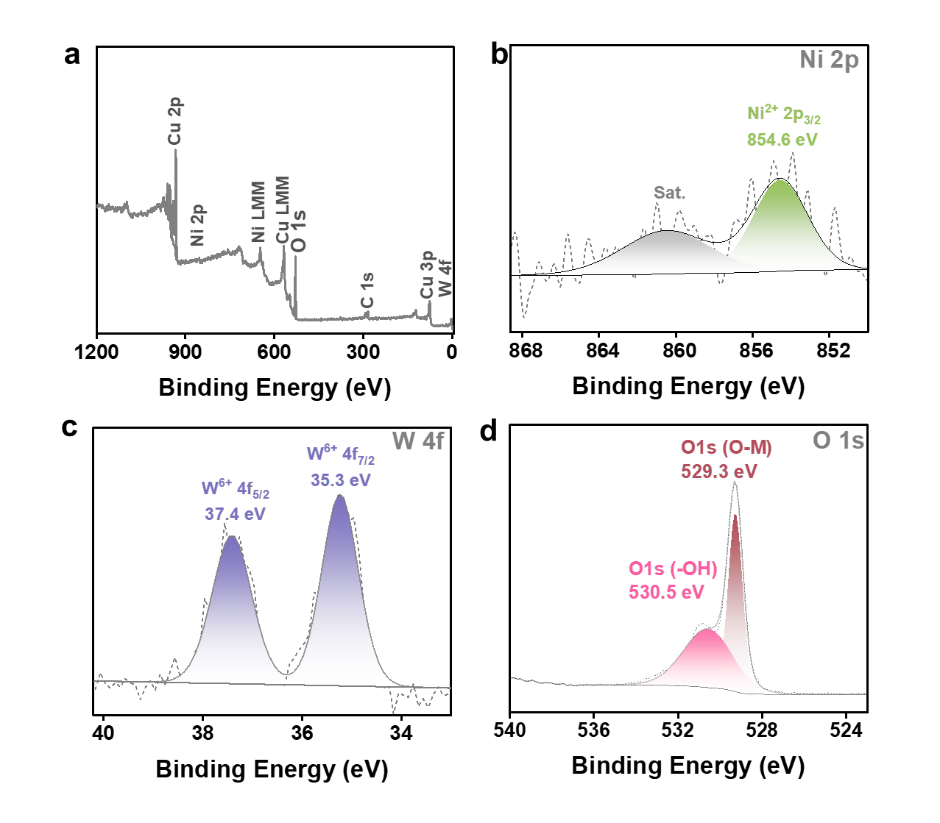
**

**Fig. S59.** (a) Survey spectrum and (b-d) high-resolution XPS spectra of bulk **E6**.

**
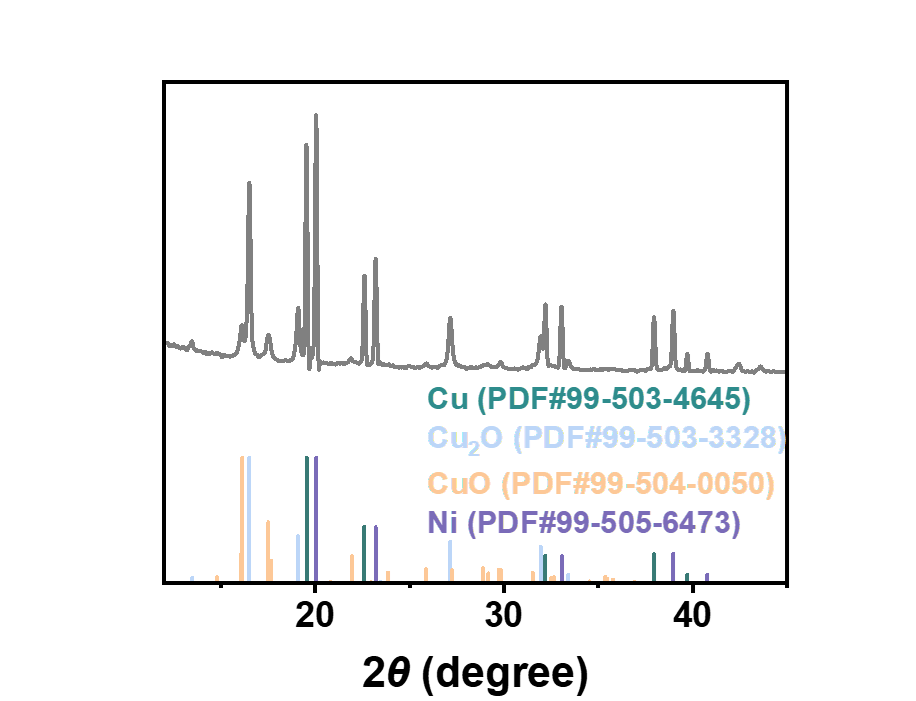
**

**Fig. S60.** pXRD data of catalyst recovered from **E6**. Recovery of the pure catalysts from the electrode was achieved by prolonged sonication in ethanol.


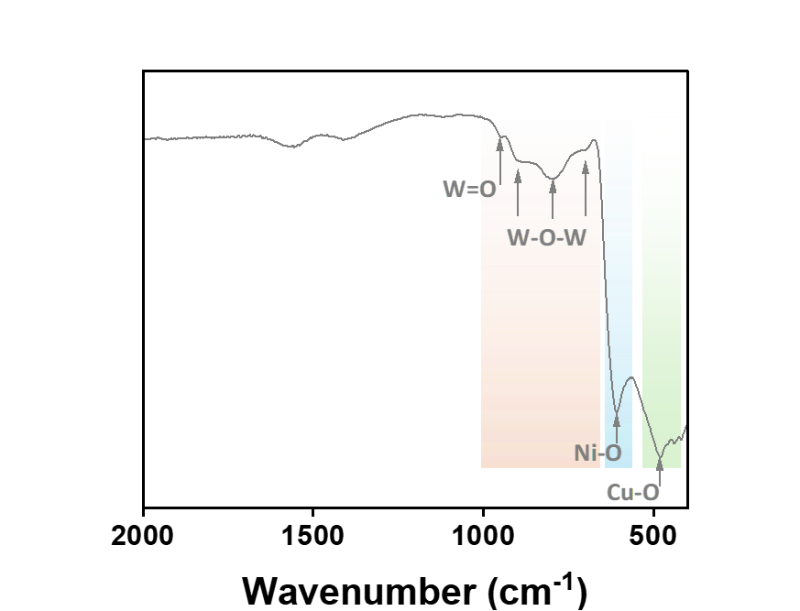


**Fig. S61.** ATR-FTIR spectra of catalyst powder removed from **E6**.


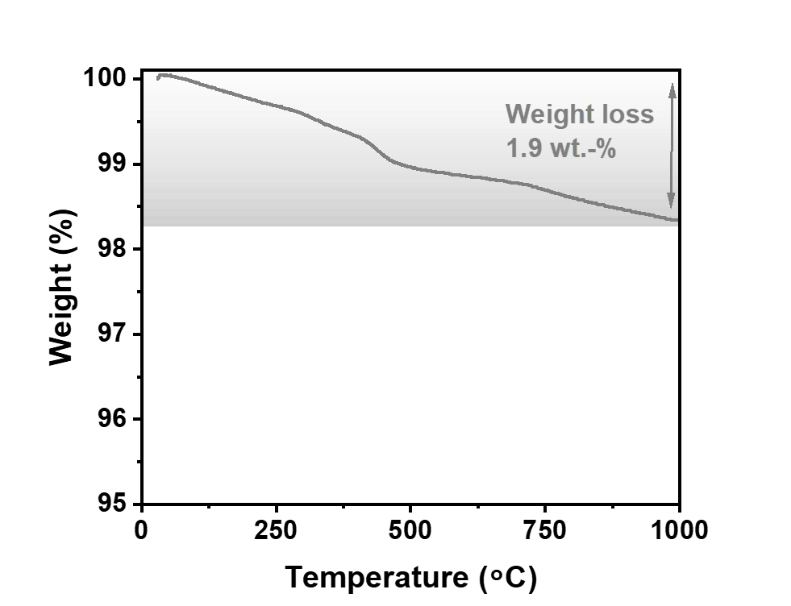


**Fig. S62.** Thermogravimetric analysis of catalyst powder removed from **E6**. Weight loss of 1.9 wt.% was observed between 30 to 1000 °C due to the loss of lattice water.

**Table S9.** Metal contents in the composite catalyst peeled off from **E6** based on ICP-OES analyses.

|  | **Cu** | **Ni** | **W** |
| --- | --- | --- | --- |
| **Mass ratio** | **1.38** | **5.23** | **1** |
| **Atomic ratio** | **4.19** | **17.18** | **1** |

**
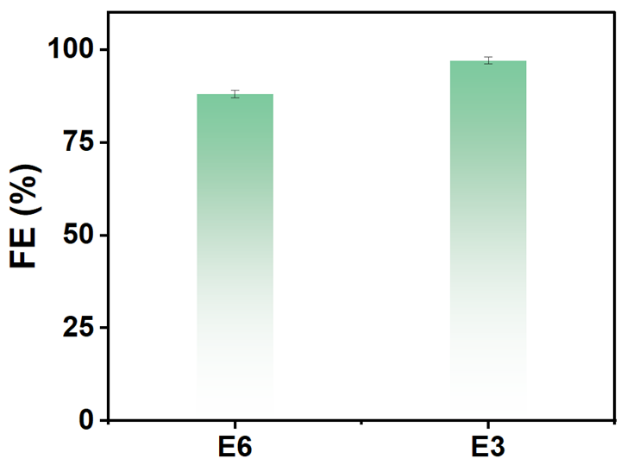
**

**Fig. S63**.NO_3_^-^RR performance comparison between **E6** and **E3**.


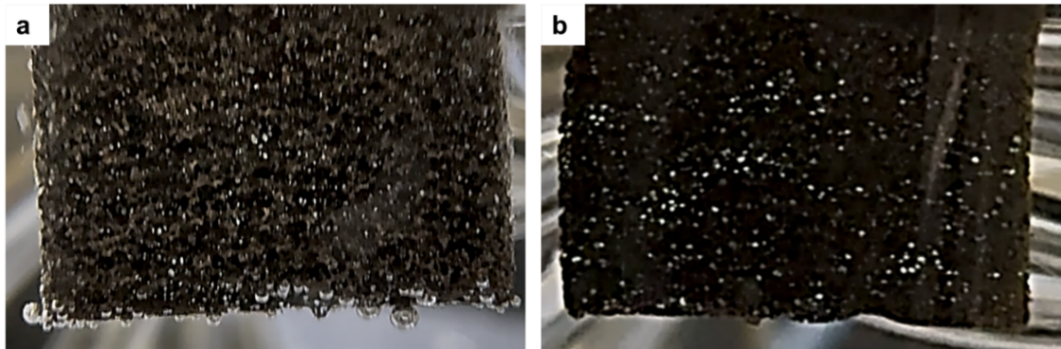


**Fig. S64**. **E6** anode employed in (a) NO_3_^-^RRIIOER and (b) NO_3_^-^RRIIGOR configurations under a cell voltage of 1.9 V.


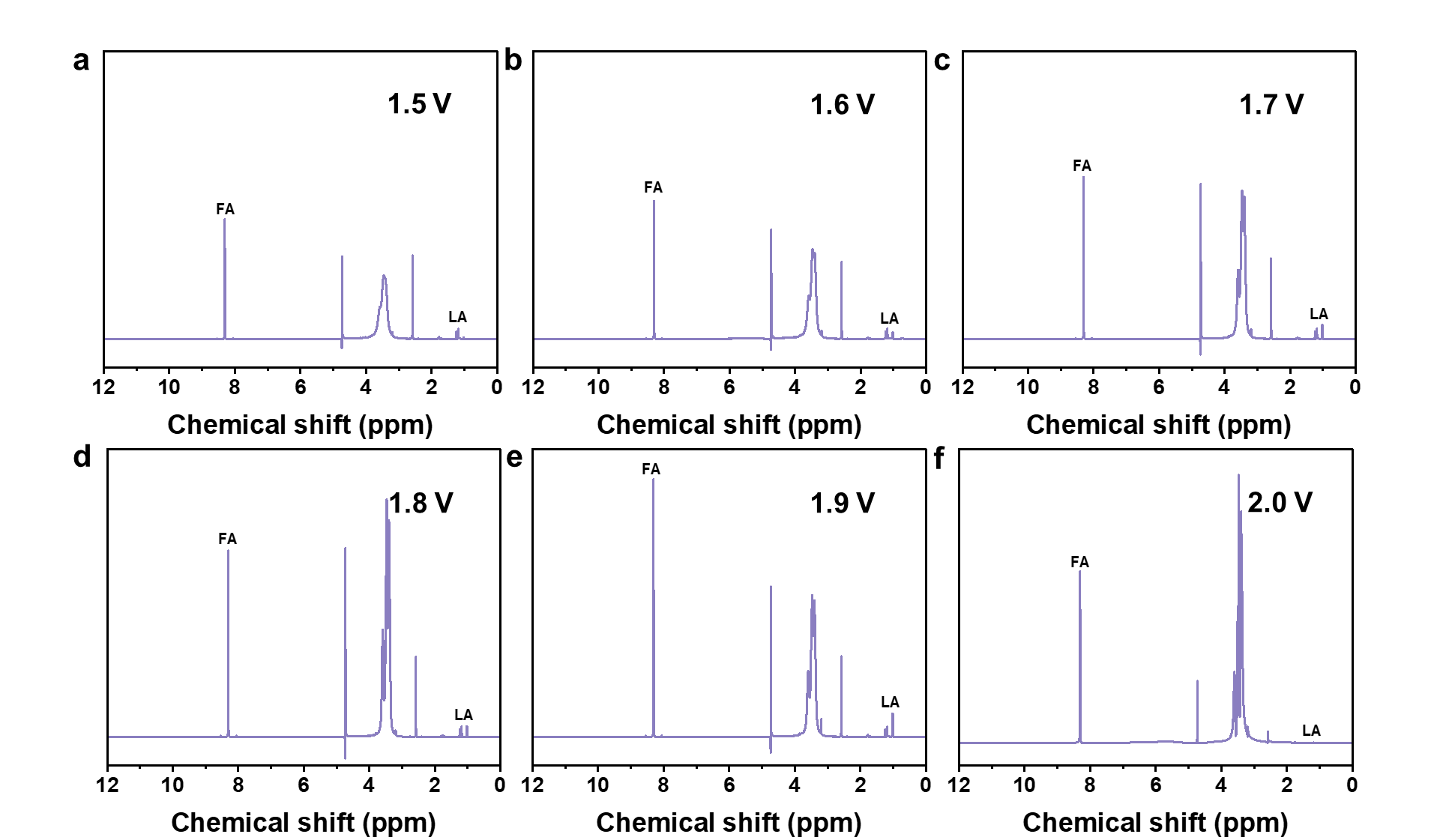


**Fig. S65.** ^1^H NMR spectra of the anodic electrolyte after GOR in the NO_3_^-^RRIIGOR configuration over a suite of cell potentials (1.5 V - 2.0 V). (FA: formic acid, LA: lactic acid)


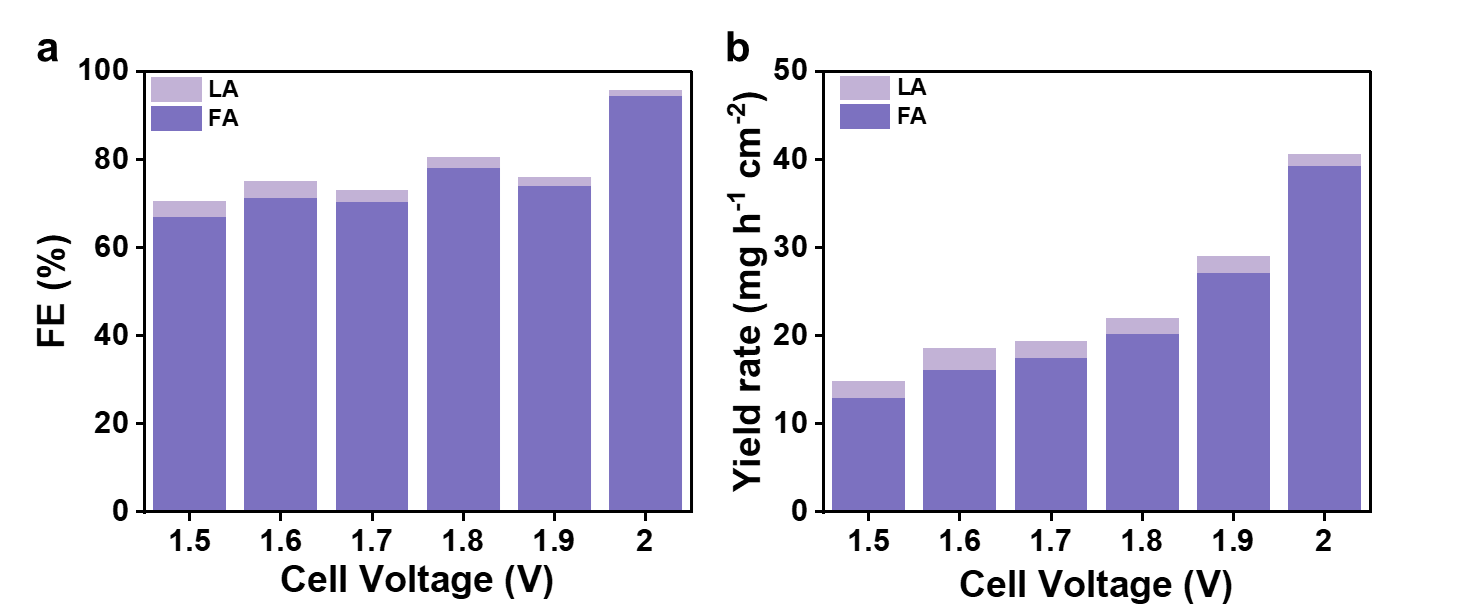


**Fig. S66.** (a) FE and (b) yield rate of FA and LA from the anodic electrolyte after GOR in the NO_3_^-^RRIIGOR configuration over a suite of cell potentials (1.5 V - 2.0 V). (FA: formic acid, LA: lactic acid)


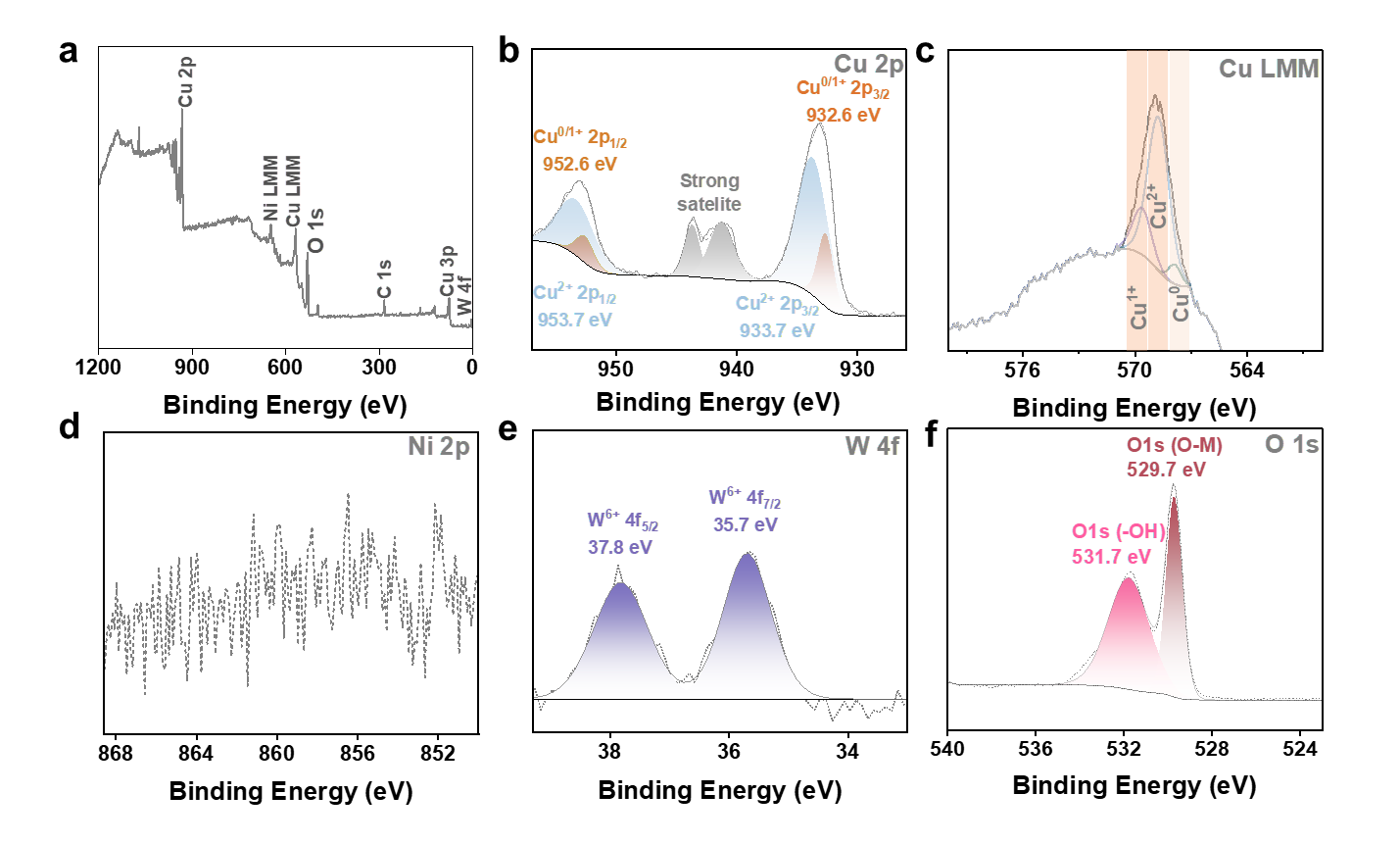


**Fig. S67**. (a) Survey spectrum and (b-f) high-resolution XPS spectra of bulk **E6** after GOR in the NO_3_^-^RRIIGOR configuration at a cell potential of 1.9 V.


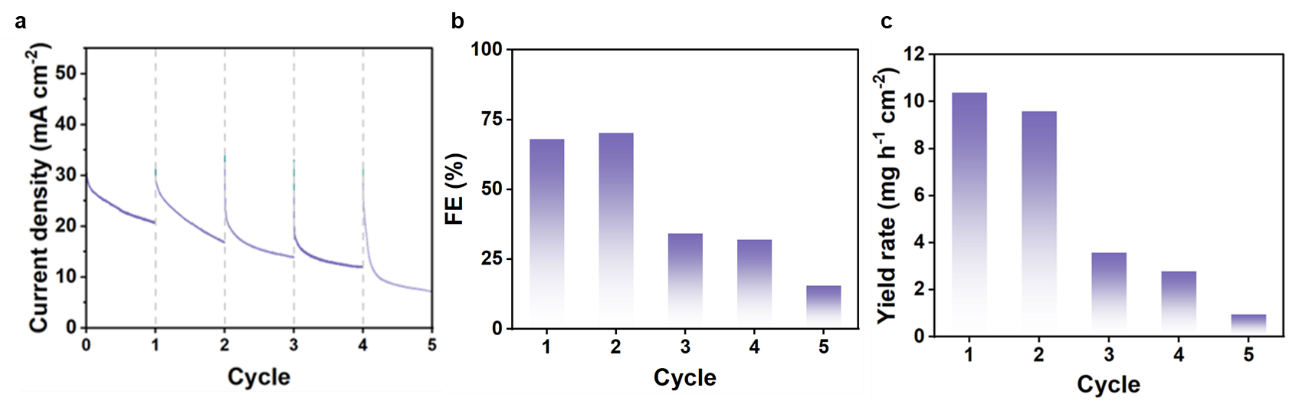


**Fig. S68**. (a) GOR cycling test in the NO_3_^-^RRIIGOR configuration at a cell potential of 1.5 V, (b) formic acid FE and (c) yield rate.

**8. References**

[1] X. Liu, T. Xie, Z. Cai, Z. Li, L. Zhang, X. Fan, D. Zhao, S. Sun, Y. Luo, Q. Liu, X. Sun, *J. Electroanal. Chem.* **2023**, *933*, 117295.

[2] K. Chandra Majhi, H. Chen, A. Batool, Q. Zhu, Y. Jin, S. Liu, P. H. L. Sit, J. Chun-Ho Lam, *Angew. Chem Int. Ed.* **2025**, *64*, e202500167.

[3] G. Kresse, J. Furthmüller, *Phys. Rev. B* **1996**, *54*, 11169.

[4] J. P. Perdew, K. Burke, M. Ernzerhof, *Phys. Rev. Lett.* **1996**, *77*, 3865.

[5] W. Kohn, L. J. Sham, *Phys. Rev* **1965**, *140*, A1133

[6] K. Mathew, R. Sundararaman, K. Letchworth-Weaver, T. A. Arias, R. G. Hennig, *J. Chem. Phys.* **2014**, *140*, 84106.

[7] X. Wang, B. Liu, S. Ma, Y. Zhang, L. Wang, G. Zhu, W. Huang, S. Wang, *Nat. Commun.* **2024**, *15*, 2600.

[8] K. Nishio, T. Miyazaki, H. Nakamura, *Phys. Rev. Lett.* **2013**, *111*, 155502.

[9] R. Tran, Z. Xu, B. Radhakrishnan, D. Winston, W. Sun, K. A. Persson, S. P. Ong, *Sci. Data* **2016**, *3*, 160080.

[10] E. O. Oseghe, F. Guba, A. Misra, R. Gong, R. Liu, S. R. Waldvogel, D. Ziegenbalg, C. Streb, D. Gao, *Device* **2023**, *1*, 100020.

[11] A. Misra, I. Franco Castillo, D. P. Müller, C. González, S. Eyssautier-Chuine, A. Ziegler, J. M. de la Fuente, S. G. Mitchell, C. Streb, *Angew. Chem. Int. Ed.* **2018**, *57*, 14926.

[12] C. Nickel, D. L. Troglauer, Z. Dallos, D. Abid, K. Sowa, M. O. Cichocka, U. Kolb, B. Mashtakov, B. F. Mohazzab, S. Han, L. Prädel, L. Ci, D. Li, X. Lin, M. Hua, R. Liu, D. Gao, *Angew. Chem. Int. Ed.* **2025**, e202424074.

[13] X. Chen, Y. Cheng, B. Zhang, J. Zhou, S. He, *Nat. Commun.* **2024**, *15*, 6278.

[14] J. Kibsgaard, T. F. Jaramillo, *Angew. Chem. Int. Ed.* **2014**, *53*, 14433.

[15] A. Chaturvedi, S. Gaber, S. Kaur, K. C. Ranjeesh, T. C. Nagaiah, D. Shetty, *ACS Energy Lett.* **2024**, *9*, 2484.

[16] L. Xiao, W. Dai, S. Mou, X. Wang, Q. Cheng, F. Dong, *Energy Environ. Sci.* **2023**, *16*, 2696.

[17] C. Li, H. Li, B. Zhang, H. Li, Y. Wang, X. Wang, P. Das, Y. Li, X. Wu, Y. Li, Y. Cui, J. Xiao, Z.-S. Wu, *Angew. Chem. Int. Ed.* **2024**, *63*, e202411542.

[18] J. Wu, X. Cheng, Y. Tong, Z. Yu, C. Lin, N. Zhang, L. Chen, P. Chen, *ACS Catal.* **2024**, *14*, 18095.

[19] X. Cheng, Z. Xie, S. Zha, Q. Xu, S. Ci, Z. Wen, *J. Mater. Chem. A* **2025**, *13*, 13286.

[20] Q. Yan, R. Zhao, L. Yu, Z. Zhao, L. Liu, J. Xi, *Adv. Mater.* **2024**, *36*, 2408680.

[21] X. Feng, J. Liu, Y. Kong, Z. Zhang, Z. Zhang, S. Li, L. Tong, X. Gao, J. Zhang, *Adv. Mater.* **2024**, *36*, 2405660.

[22] Y. Zhang, T. Gao, F. Zhang, X. Qu, Y. Luo, P. Zhang, J. Liang, Y. Song, F. Fang, F. Wang, D. Sun, Y. Liu, *Adv. Energy Mater.* **2024**, *14*, 2401834.

[23] X. Zhao, G. Hu, F. Tan, S. Zhang, X. Wang, X. Hu, A. V Kuklin, G. V Baryshnikov, H. Ågren, X. Zhou, H. Zhang, *J. Mater. Chem. A* **2021**, *9*, 23675.

[24] H. Jiang, G.-F. Chen, O. Savateev, J. Xue, L.-X. Ding, Z. Liang, M. Antonietti, H. Wang, *Angew. Chem. Int. Ed.* **2023**, *62*, e202218717.

[25] Y. Fu, S. Wang, Y. Wang, P. Wei, J. Shao, T. Liu, G. Wang, X. Bao, *Angew. Chem. Int. Ed.* **2023**, *62*, e202303327.

[26] J.-Y. Fang, Q.-Z. Zheng, Y.-Y. Lou, K.-M. Zhao, S.-N. Hu, G. Li, O. Akdim, X.-Y. Huang, S.-G. Sun, *Nat. Commun.* **2022**, *13*, 7899.

[27] H. Liu, J. Li, F. Du, L. Yang, S. Huang, J. Gao, C. Li, C. Guo, *Green Energy Environ.* **2023**, *8*, 1619.

[28] J. Wang, Y. Wang, C. Cai, Y. Liu, D. Wu, M. Wang, M. Li, X. Wei, M. Shao, M. Gu, *Nano Lett.* **2023**, *23*, 1897.

[29] W. Wen, P. Yan, W. Sun, Y. Zhou, X.-Y. Yu, *Adv. Funct. Mater.* **2023**, *33*, 2212236.

[30] J. Li, J. Gao, T. Feng, H. Zhang, D. Liu, C. Zhang, S. Huang, C. Wang, F. Du, C. Li, C. Guo, *J. Power Sources* **2021**, *511*, 230463.

[31] Y. Yao, X. Wei, H. Zhou, K. Wei, B. Kui, F. Wu, L. Chen, W. Wang, F. Dai, P. Gao, N. Wang, W. Ye, *ACS Catal.* **2024**, *14*, 16205.

[32] K. Liu, H. Li, M. Xie, P. Wang, Z. Jin, Y. Liu, M. Zhou, P. Li, G. Yu, *J. Am. Chem. Soc.* **2024**, *146*, 7779.

[33] W. Liu, J. Chen, Y. Wei, Y. He, Y. Huang, M. Wei, Y. Yu, N. Yang, W. Zhang, L. Zhang, F. Saleem, F. Huo, *Adv. Funct. Mater.* **2024**, *34*, 2408732.

[34] R. Boppella, M. Ahmadi, B. M. Arndt, D. R. Lustig, M. Nazemi, *ACS Catal.* **2024**, *14*, 18223.

[35] W. Zhong, X. Xiang, P. Chen, J. Su, Z. Gong, X. Liu, S. Zhao, N. Zhang, C. Feng, Z. Zhang, Y. Chen, Z. Lin, *Chem Catal.* **2025**, *5*, 101182.

[36] Y. Dai, S. Li, X. Li, K. Liu, Y. Guo, H. Li, B. Jiang, *Adv. Funct. Mater.* **2025**, *35*, 2420282.

[37] X. Ouyang, W. Qiao, Y. Yang, B. Xi, Y. Yu, Y. Wu, J. Fang, P. Li, S. Xiong, *Angew. Chem. Int. Ed.* **2025**, *64*, e202422585.

[38] K. Chandra Majhi, H. Chen, A. Batool, Q. Zhu, Y. Jin, S. Liu, P. H.-L. Sit, J. Chun-Ho Lam, *Angew. Chem. Int. Ed.* **2025**, *64*, e202500167.

[39] J. Li, L. Liu, S. Huang, H. Wang, Y. Tang, C. Zhang, F. Du, R. Ma, C. Li, C. Guo, *Adv. Funct. Mater.* **2025**, *35*, 2501527.

[40] K. Zhang, B. Li, F. Guo, N. Graham, W. He, W. Yu, *Angew. Chem. Int. Ed.* **2024**, *63*, e202411796.

[41] Y. Bu, W. Yu, Q. Yang, W. Zhang, Q. Sun, W. Wu, P. Cui, C. Wang, G. Gao, *Environ. Sci. Technol.* **2024**, *58*, 12708.

[42] Y. Bu, W. Yu, W. Zhang, C. Wang, J. Ding, G. Gao, *Nano Lett.* **2024**, *24*, 2812.

[43] F. Dou, F. Guo, B. Li, K. Zhang, N. Graham, W. Yu, *J. Hazard. Mater.* **2024**, *472*, 134522.

[44] Q. Yang, Y. Bu, S. Pu, L. Chu, W. Huang, X. Zhu, C. Liu, G. Fang, P. Cui, D. Zhou, Y. Wang, *Angew. Chem. Int. Ed.* **2024**, *63*, e202400428.

[45] R. Boppella, M. Ahmadi, B. M. Arndt, D. R. Lustig, M. Nazemi, *ACS Catal.* **2024**, *14*, 18223.

[46] Z. Li, Z. Shi, Y. Ou, L. Zhong, C. Yan, C. Zhang, K. Song, H. Liu, D. Liu, P. Song, C. Yin, Z. Qi, L. Song, C. Lv, *Angew. Chem. Int. Ed.* **2025**, *64*, e202510287.

[47] K. Zhang, G. Liu, Q. Wang, X. Huo, X. Zou, M. Tang, X. Zhang, L. An, *Adv. Sci.* **2025**, *12*, e07720.

**9. Author contributions**

C.N. and D.T. contributed equally to the manuscript. C.N., D.T. and D.G. conceived the conceptual design of the project. C.N. and D.T. carried out the electrocatalyst synthesis. C.N., D.T., B.M., K.S., I.L., C.C, B.M., T.S. participated in material characterization and data curation. C.N. and D.T. performed the electrocatalytic studies. C.C. conducted XAS experiments and data analyses. T.B. carried out electrochemical online DEMS tests. X.L. performed the DFT calculation. D.L., B. H., R.L., X.L. and D.G. acquired funding and provided project administration. The draft was written and edited by C. N. and D. T.. The manuscript was reviewed through the contributions of the other authors. All authors have approved the submission of the final version.
